# Synthesis and Biological Evaluation of Water‐Soluble Esterase‐Activated CO‐Releasing Molecules Targeting Mitochondria

**DOI:** 10.1002/chem.202201670

**Published:** 2022-07-14

**Authors:** Lars Hemmersbach, Yannick Schreiner, Xinmiao Zhang, Finn Dicke, Leon Hünemeyer, Jörg‐Martin Neudörfl, Thomas Fleming, Benito Yard, Hans‐Günther Schmalz

**Affiliations:** ^1^ Department of Chemistry Universität zu Köln Greinstrasse 4 50939 Köln Germany; ^2^ Vth Medical Department Medical Faculty Mannheim University of Heidelberg Theodor-Kutzer-Ufer 1–3 68167 Mannheim Germany; ^3^ Department of Internal Medicine I and Clinical Chemistry University Hospital of Heidelberg 69120 Heidelberg Germany; ^4^ German Center for Diabetes Research (DZD) 85764 Neuherberg Germany

**Keywords:** carbon monoxide, esterases, inflammation, iron carbonyl complexes, prodrugs

## Abstract

Due to the beneficial effects of carbon monoxide as a cell‐protective and anti‐inflammatory agent, CO‐releasing molecules (CORMs) offer some promising potential applications in medicine. In this context, we synthesized a set of acyloxy‐cyclohexadiene‐Fe(CO)_3_ complexes, all displaying a *N*‐methyl‐pyridinium triflate moiety in the ester side chain, as mitochondria‐targeting esterase‐triggered CORM prodrugs. Whereas the compounds in which the acyloxy substituent is attached to the 2‐position of the diene‐Fe(CO)_3_ unit (A series) spontaneously release CO upon dissolution in phosphate buffer, which remarkably is partly suppressed in the presence of porcine liver esterase (PLE), the 1‐substituted isomers (B series) show the expected PLE‐induced release of CO (up to 3 equiv.). The biological activity of Mito‐CORMs **2**/**3**‐**B** and their isophorone‐derived analogs **2/3**‐**A**’, which also displayed PLE‐induced CO release, was assessed by using human umbilical vein endothelial cells (HUVEC). Whereas Mito‐CORMs **2/3**‐**B** were not cytotoxic up to 500 μM (MTT assay), Mito‐CORMs **2**/**3**‐**A**’ caused significant toxicity at concentrations above 50 μM. The anti‐inflammatory potential of both Mito‐CORM variants was demonstrated by concentration‐dependent down‐regulation of the pro‐inflammatory markers VCAM‐1, ICAM‐1 and CXCL1 as well as induction of HO‐1 in TNFα‐stimulated human umbilical vein endothelial cells (HUVECs; western blotting and qPCR). Energy phenotyping by seahorse real‐time cell metabolic analysis, revealed opposing shifts of metabolic potentials in cells treated either with Mito‐CORMs **2/3**‐**B** (increased mitochondrial respiration and glycolytic activity) or Mito‐CORMs **2/3**‐**A**’ (suppressed mitochondrial respiration and increased glycolytic activity). Thus, the Mito‐CORMs represent valuable tools for the safe and targeted delivery of CO to mitochondria as a subcellular compartment to induce positive anti‐inflammatory effects with only minor shifts in cellular energy metabolism. Also, due to their water solubility, these compounds provide a promising starting point for further pharmacological studies.

## Graphical abstract

**An ionic moiety makes the difference**. A pyridinium salt in the ester side chain not only improves the water solubility of acyloxydiene‐Fe(CO)_3_ complexes but also directs these prodrugs to mitochondria, where they are triggered by an esterase to release carbon monoxide as an anti‐inflammatory and cytoprotective agent directly effecting the metabolic state of the cells.

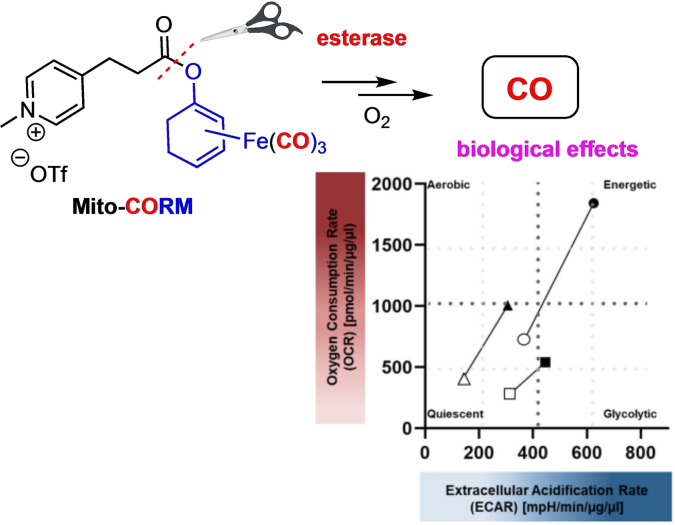

## Introduction

**Figure 1:**
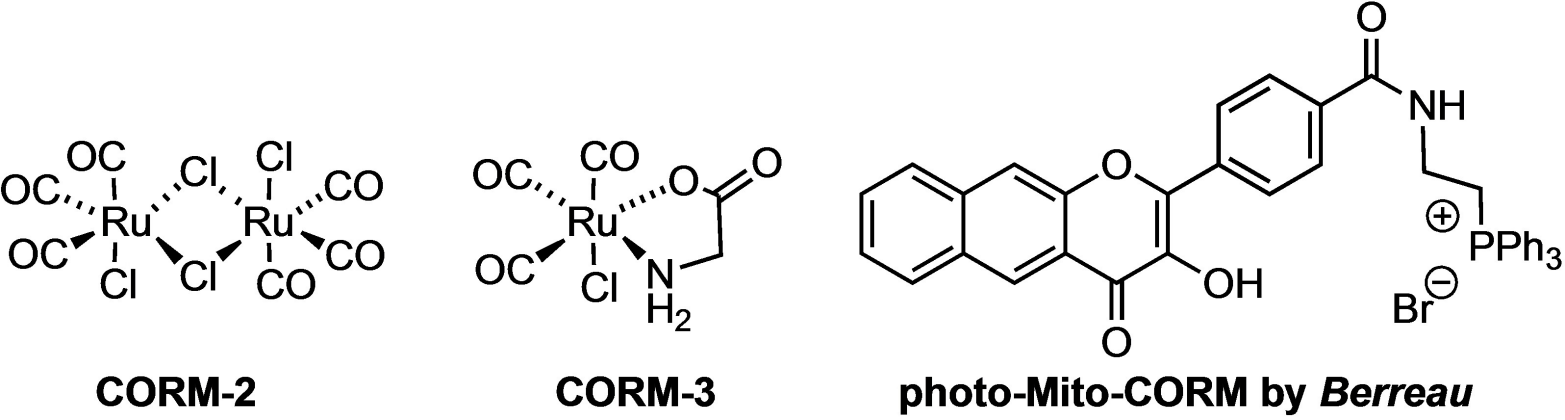
Structures of **CORM**‐**2**, **CORM**‐**3** and the first Mito‐CORM.[^4^, ^12^]

Carbon monoxide, often considered merely a toxic gas, has been identified in recent decades as an important biological signaling molecule (gasotransmitter),^[1]^ which at low concentrations exhibits pronounced and beneficial physiological effects, such as anti‐inflammatory or cytoprotective activities.^[2]^ Because the administration of gaseous CO carries a high risk of poisoning, the search for alternative means of controlled (targeted) CO release is an important research task. In this context, several research groups have focused on the development of CO‐releasing molecules (CORMs) to selectively introduce CO into an organism.^[3]^ While the first CORMs, such as **CORM**‐**2** and **CORM**‐**3** (Figure 1), were designed to release CO spontaneously by ligand exchange from metal carbonyl complexes,^[4]^ several other types of CORMs with different release mechanisms were developed and successfully tested in biological experiments.^[3]^ In particular, light is frequently used to cause a controlled release of CO from so‐called photo‐CORMs.^[5]^ Recently, Wang and co‐workers developed organic CO prodrugs based on decarbonylation chemistry, some of which also allow triggered CO release, for example, by esterases, pH change, or oxidation (ROS).^[6]^

**Scheme 1:**
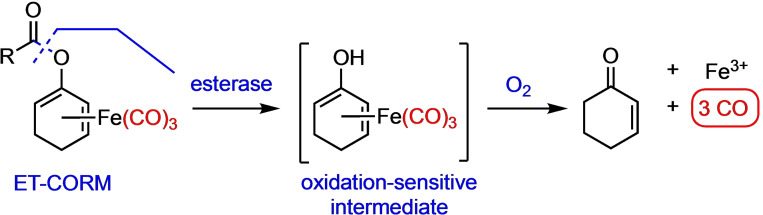
General mechanism of esterase‐triggered CO release from acyloxy‐diene‐Fe(CO)_3_ complexes (ET‐CORMs).^[7b]^

In the course of our own research program, we have developed acyloxy‐diene‐Fe(CO)_3_ complexes as enzyme‐triggered CO‐releasing molecules (ET‐CORMs). These molecules are typically activated by esterase‐induced cleavage of the ester function to generate an oxidation‐sensitive dienol‐iron carbonyl complex that yields up to three equivalents of CO under physiological (oxidative) conditions in addition to Fe^3+^ ions and cyclohexenone (Scheme 1).^[7]^

The mechanism(s) of the cellular effects of CO are yet not fully understood, however, a major contribution results from the affinity of CO to the transition metal centers of metalloproteins such as hemoglobin or cytochrome c oxidase (mitochondrial complex IV).[^1c^, ^8^] Accordingly, mitochondria appear to play a key role in CO action, as inhibition of cytochrome c oxidase causes oxidative phosphorylation and inhibition of apoptosis pathways, which might contribute to the reported cytoprotective effect of CO.[^1c^, ^8b^, ^9^] Early investigations of the effect of CO on mitochondria were performed using either gaseous CO or first generation CORMs, such as **CORM**‐**2** and **CORM**‐**3**.[^9b^, ^10^] However, as reported by various research groups, the amounts of CO released from **CORM**‐**2** or **CORM**‐**3** are small and undefined, and some effects of these compounds may even be caused by the ruthenium rather than the CO.[^7g^, ^11^] Therefore, alternative compounds need to be tested to determine the action of CO on mitochondria in a meaningful concentration‐dependent manner. In 2018, Otterbein and Wang reported a cleverly designed molecular system that enables the targeted delivery of carbon monoxide to mitochondria through enrichment‐triggered prodrug activation.^[12]^ In the same year, the groups of Berreau and Benninghoff were the first to synthesize and study a mitochondrion‐specific photo‐CORM (Figure 1).^[13]^ The latter authors concluded that an intra‐mitochondrial release of even very small amounts (10 μM) of CO is as equally effective as the release of equal amounts of CO in the cytosol.^[13]^ A micellar system designed for the enhancement of ferroptosis by mitochondria‐targeting co‐release of CO and other active agents has also been reported.^[14]^

In a recent study, we demonstrated that the anti‐inflammatory properties of CORMs depend on the amount of CO released rather then intra‐ or extra‐cellular production,^[7g]^ in accordance to the results of Berreau.^[15]^ Given the promising properties of our iron‐based ET‐CORMs, we were now interested in whether such compounds could also be used to study CO release in specific organelles compared with normal intracellular CO release. For the design of ET‐CORMs that selectively release CO into mitochondria, we considered two types of lipophilic cationic moieties (alkyl triphenyl phosphonium salts,^[16]^ and pyridinium or pyrimidinium salts)^[17]^ which are known to render potential drugs mitochondria‐selective agents. Noteworthy, such Mito‐CORMs should not only ensure localized CO release, but also exhibit an increased water solubility.^[7c]^ We here report the synthesis and characterization of a series of such Mito‐CORMs which indeed proved to be water‐soluble and exhibited some interesting CO‐releasing properties and biological activities.

## Results and Discussion

### Synthesis and chemical characterization of Mito‐CORMs

**Scheme 2:**
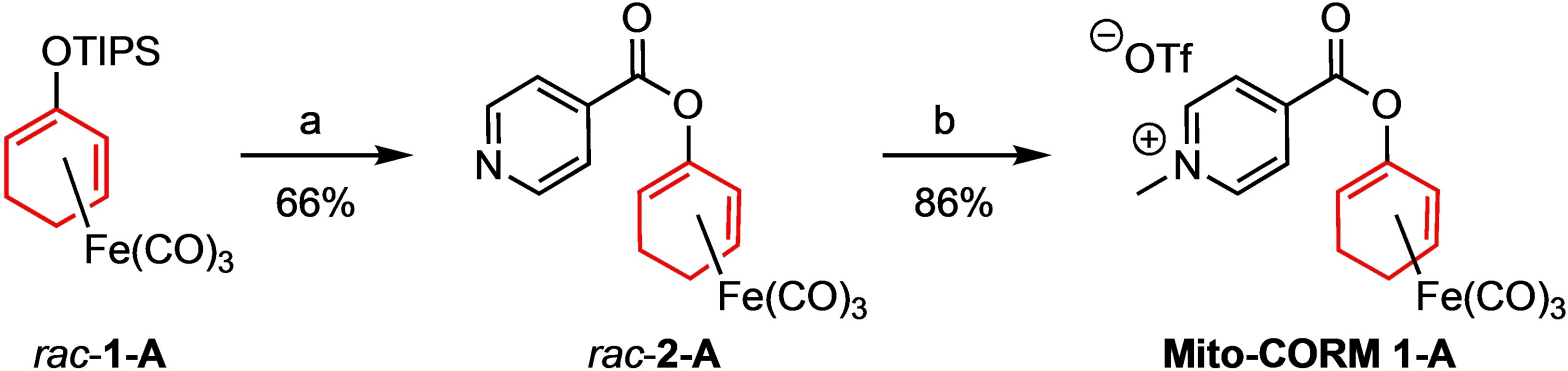
Synthesis of Mito‐CORM **1**‐**A**. a) TBAF, pyridine, CH_2_Cl_2_, RT, 30 min then addition of EDC ⋅ HCl, DMAP and isonicotinic acid, RT, 2 h; b) MeOTf, Et_2_O, RT, 30 min. TBAF: tetrabutylammonium fluoride; EDC: 1‐ethyl‐3‐(3‐dimethylaminopropyl)‐carbodiimide; DMAP: *N,N*‐4‐dimethyl‐aminopyridine; MeOTf: methyl triflate.

**Figure 2:**
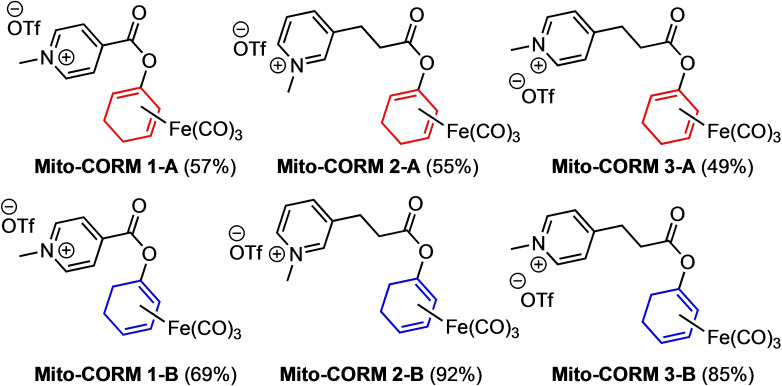
Structures of synthesized Mito‐CORMs derived from cyclohex‐2‐enone (yields over two steps in brackets).

The synthesis of a first class of Mito‐CORMs started from the silyloxydiene‐Fe(CO)_3_ complex *rac*‐**1**‐**A**, which was prepared from 2‐cyclohexenone as previously reported.^[7b]^ After fluoride‐induced desilylation of *rac*‐**1**‐**A**, esterification of the resulting dienol complex with isonicotinic acid afforded the ester *rac*‐**2**‐**A**. Subsequent N‐methylation with methyl triflate gave Mito‐CORM **1**‐**A** in good yield (Scheme 2). Following the same general protocol, a total of six new Mito‐CORMs were synthesized (Figure 2). It is worth mentioning that all complexes were obtained in racemic form and the compounds of the series A and B differ in the position of the oxy‐substituent at the diene‐Fe(CO)_3_ moiety.

In addition to Mito‐CORMs **1**‐**A** and **1**‐**B**, derived from isonicotinic acid, we also prepared Mito‐CORMs **2**‐**A/B** and **3**‐**A/B** which were derived from 3‐ and 4‐pyridine propionic acid, respectively (Figure 2). By varying the length of the linker between the ester and the pyridinium moiety, we expected different chemical and biological properties. For example, it was expected that compounds with a longer linker would exhibit higher stability to hydrolysis, an issue which we became aware of during our experiments (see below). Furthermore, the higher lipophilicity of these compounds would also lead to increased cell permeability. It is noteworthy that all six new Mito‐CORMs were found to have high water solubility (e. g., 10 mg/mL for Mito‐CORM **2**‐**B**), a desired property facilitating biological experiments.

**Figure 3:**
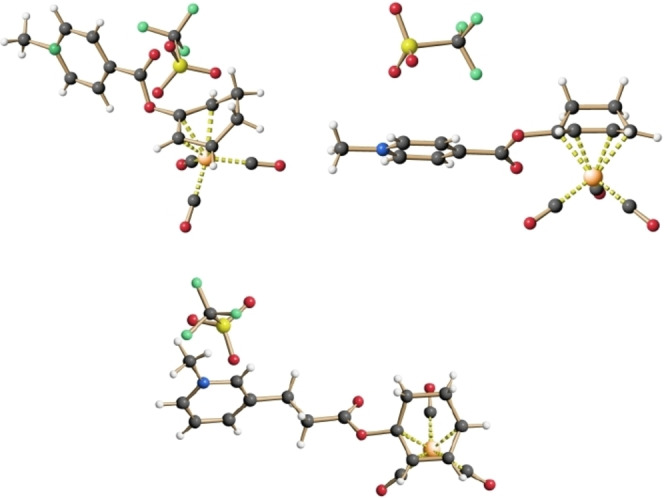
Structure of Mito‐CORMs **1**‐**A** (top left), **1**‐**B** (top right) and **2**‐**B** (bottom) in the crystalline state.

While all of the six new Mito‐CORMs showed the expected spectroscopic data, three of them could be additionally characterized by X‐ray crystallography as unambiguously proof of their structure (Figure 3).

**Figure 4:**
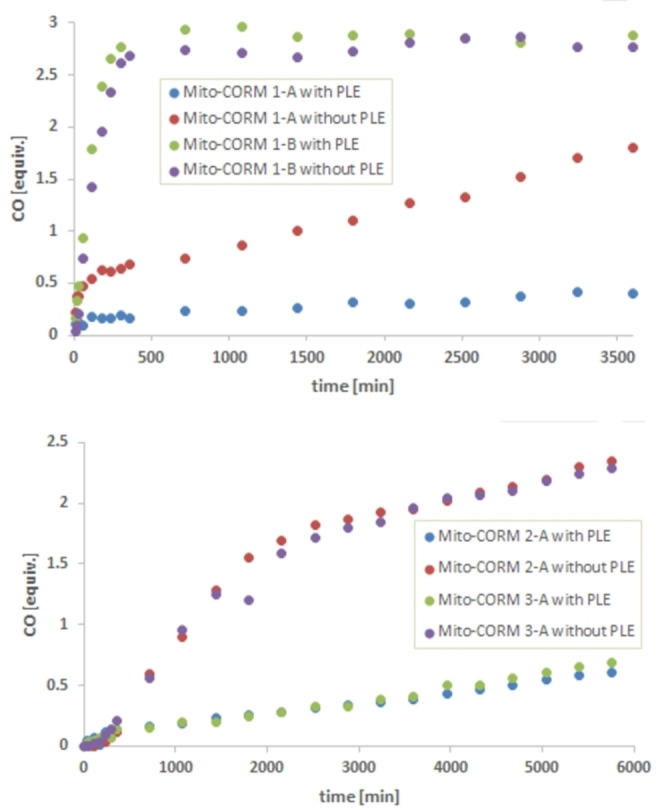
In‐vitro CO release from Mito‐CORMs **1**‐**A/B** (top) and Mito‐CORMs **2/3**‐**A** (bottom) in phosphate buffer (0.1 M; pH 7.4)/DMSO=5 : 1 as detected by headspace GC.

Next, the Mito‐CORMs prepared so far were analyzed for their CO‐releasing properties. Headspace gas chromatography was used to quantify CO release in vitro using a 5 : 1 mixture of phosphate buffer (0.1 M; pH 7.4) and DMSO as the solvent. Mito‐CORM **1**‐**A** proved to be sensitive towards hydrolysis to liberate 1.8 equiv. of CO after 2.5 days even in the absence of porcine liver esterase (PLE). Noteworthy, the spontaneous CO release from this compound was strongly suppressed in the presence of PLE (Figure 4).

**Figure 5:**
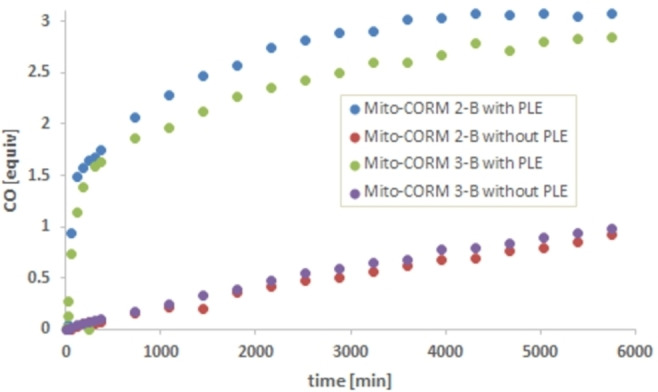
In vitro CO release from Mito‐CORMs **2/3**‐**B** in phosphate buffer (0.1 M; pH 7.4)/DMSO=5 : 1 as detected by headspace GC.

In the case of Mito‐CORM **1**‐**B**, near‐quantitative CO release (3.0 equiv.) was observed after only 10 h, both in the presence and absence of PLE (Figure 4, top). The spontaneous CO release (in the absence of PLE) can be explained by the highly activated ester bond which is correspondingly susceptible to hydrolysis. Interestingly, Mito‐CORMs **2**‐**A** and **3**‐**A** with an extended linker between the ester and the pyridinium moiety also showed a spontaneous CO release (2 equiv. after 2 days), which in turn was strongly suppressed in the presence of PLE (only 0.6 equiv. after 5 days; Figure 4, bottom). In the case of the isomeric compounds (B series), namely Mito‐CORMs **2/3**‐**B**, a significantly higher amount of CO was released in the presence of PLE (ca. 3.0 equiv.) compared to the spontaneous release without PLE (ca. 1.0 equiv.; Figure 5). This desired behavior (esterase‐triggered CO release) makes Mito‐CORMs **2/3**‐**B** suitable candidates for biological testing.

**Scheme 3:**
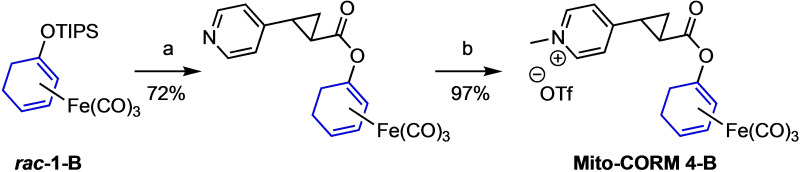
Synthesis of Mito‐CORM **4**‐**B**. a) TBAF, pyridine, CH_2_Cl_2_, RT, 30 min, then addition of EDC ⋅ HCl, DMAP and *rac‐*4‐(2‐carboxycyclopropyl)pyridin‐1‐ium chloride, RT, 21 h; b) MeOTf, Et_2_O, RT, 90 min.

Currently, we have no explanation for this “catalytic protective effect” of PLE, which is only observed for the “inner” Mito‐CORMs (A series). To exclude a radical chain oxidation mechanism, selected CO release experiments were repeated in the presence of radical traps such as 2,6‐di‐*tert*‐butyl‐4‐methylphenol (BHT) or TEMPO, but without significant effect. To probe whether the unexpectedly high spontaneous CO release from Mito‐CORMs **2**‐**A** and **3**‐**A** in the absence of PLE results from an activation of the ester function towards hydrolysis through intramolecular interaction of the ester carbonyl group with the positive charged pyridinium moiety, we synthesized Mito‐CORM **4**‐**B** to ensure an electronic and steric separation of the two functionalities by introducing a *trans*‐cyclopropane unit. As shown in Scheme 3, Mito‐CORM **4**‐**B** was successfully synthesized (as an *ambo* mixture of stereoisomers) employing racemic 4‐(2‐carboxycyclopropyl)pyridin‐1‐ium chloride (for details, see the Supporting Information).

**Figure 6:**
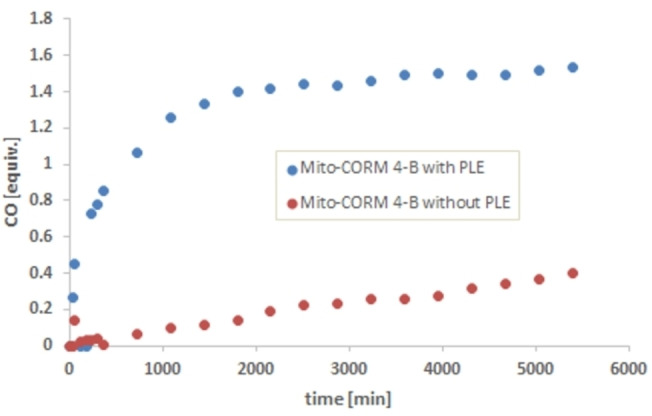
In‐vitro CO release from Mito‐CORM **4**‐**B** in phosphate buffer (0.1 M; pH 7.4)/DMSO=5 : 1 as detected by headspace GC.

CO release experiments then showed that Mito‐CORM **4**‐**B** has a lower tendency to spontaneously release CO compared to Mito‐CORMs **2/3**‐**B** (Figure 6). However, in the presence of PLE, Mito‐CORM **4**‐**B** releases only 1.5 equivalents of CO and still shows a some background CO release (ruling out a possible intramolecular activation of the ester function. Also because of its more laborious synthesis, this compound (mixture) was not included in the biological studies.

**Figure 7:**
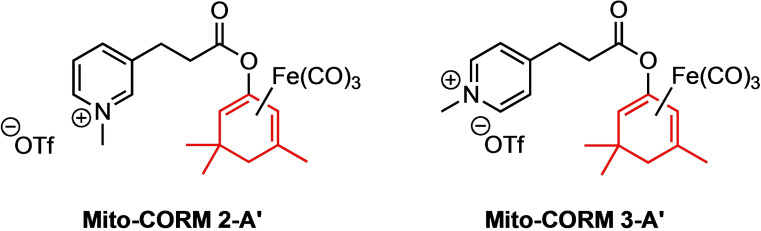
Synthesized Mito‐CORMs **2/3**‐**A**’ derived from isophorone.

We also considered that cyclohex‐2‐enone, which is formed during esterase‐induced activation of the previously discussed Mito‐CORMs (Scheme 1),^[7b]^ might partly be oxidized to phenol, a highly toxic substance^[18]^ that could contribute to the observed toxicity of cyclohexanone‐derived ET‐CORMs. Therefore, we additionally synthesized Mito‐CORMs **2/3**‐**A**’ (Figure 7) derived from isophorone, which cannot be easily oxidized to a phenol and/or other toxic aromatic compounds under physiological conditions. Details of the synthesis are given in the Supporting Information.

**Figure 8:**
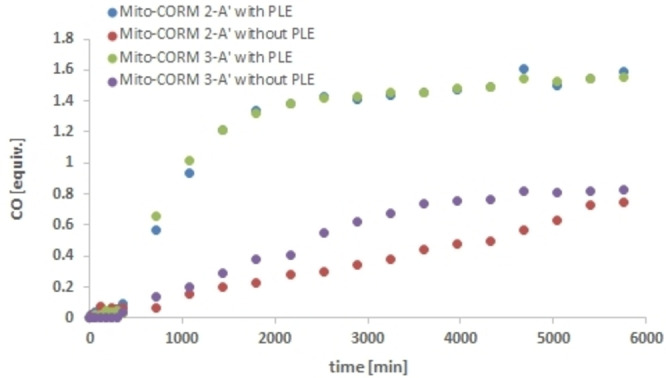
In‐vitro CO release from Mito‐CORM **2/3**‐**A**’ in phosphate buffer (0.1 M; pH 7.4)/DMSO=5 : 1 as detected by headspace GC.

In contrast to the Mito‐CORMs **1/2**‐**A**, the isophorone‐derived Mito‐CORMs **2/3**‐**A**’ exhibited the expected esterase‐induced CO‐releasing properties, liberating more CO (up to 1.5 equiv.) in the presence of PLE than in its absence (Figure 8). It is noteworthy that these compounds formally belong to the class of “inner”‐diene complexes (A series). However, in comparison to Mito‐CORMs **1/2**‐**A** their tendency to spontaneously release CO appears to be decreased due to steric demand of the isophorone core (compare also Figure S64). Therefore, we considered Mito‐CORMs **2/3**‐**A**’ alongside Mito‐CORMs **2/3**‐**B** as suitable candidates for the biological studies.

### Biological Investigations

As relevant biological properties, the cell toxicity, the inhibition of TNFα‐mediated inflammatory markers (VCAM‐1, ICAM‐1 and CXCL1),^[19]^ the induction of heme oxygenase 1 (HO‐1)^[20]^ as well as energy phenotyping^[21]^ of the selected Mito‐CORMs were investigated in human umbilical vein endothelial cells (HUVECs).

**Figure 9:**
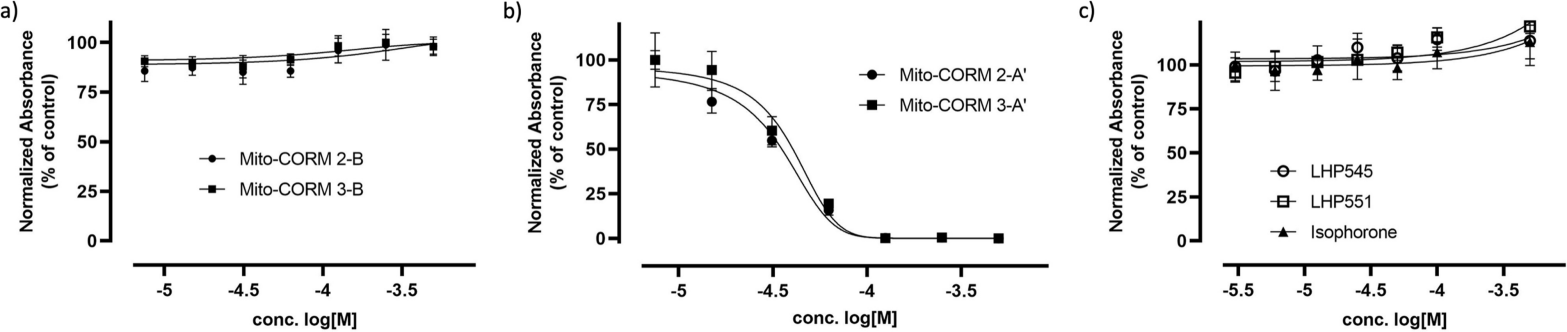
Cell toxicity of Mito‐CORMs. HUVECs were cultured overnight in 96‐well plates and in the presence of different concentrations of a) Mito‐CORMs **2/3**‐**A‘**, b) Mito‐CORMs **2/3**‐**B**. c) As a control, isophorone and the esters LHP545 and LHP551 were also investigated. Toxicity was assessed by MTT. The results of representative experiments (*n*=3) are expressed as normalized absorbance (% of control/untreated).

MTT assays,^[22]^ used as a measure of viability, showed that the cyclohexanone‐derived Mito‐CORMs **2/3**‐**B** did not affect the viability of HUVEC cells cultured overnight in the presence of the compounds at concentrations up to 1 mM (Figure 9a). In contrast, the isophorone‐derived Mito‐CORMs **2/3**‐**A**’, designed to avoid the formation of phenol and therefore thought to be less toxic, displayed significant toxicity at concentrations above 50 μM (Figure 9b). As a control, the hydrolysis product isophorone as well as the methyl esters (LHP545 and LHP551) corresponding to the Mito‐CORMs investigated were found to be non‐toxic (Figure 9c), suggesting that the toxicity is likely mediated by the released CO although more CO is released from Mito‐CORMs **2/3**‐**B** as compared to the **2/3**‐**A**’ variants in the presence of PLE in vitro (3 equiv. vs. 1.5 equiv.).

**Figure 10:**
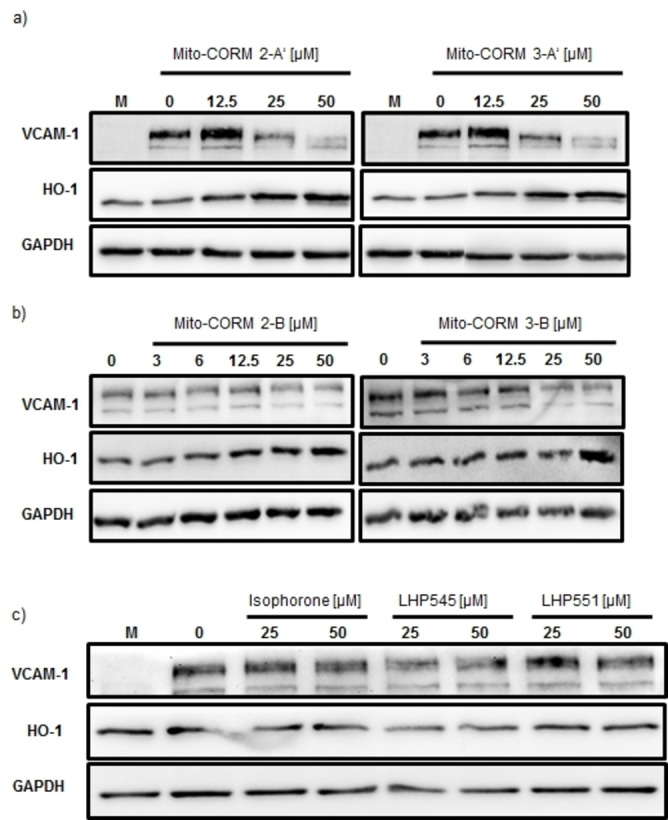
Inhibition of VCAM‐1 expression and induction of HO‐1 by Mito‐CORMs. HUVEC were cultured overnight in six‐well plates and stimulated with TNFα in the presence of different concentrations of a), b) Mito‐CORMs or c) their respective hydrolysis products. HUVEC cultured in normal medium was included to demonstrate upregulation of VCAM‐1 expression by TNFα. GAPDH was used to confirm equal loading of the gel.

**Figure 11:**
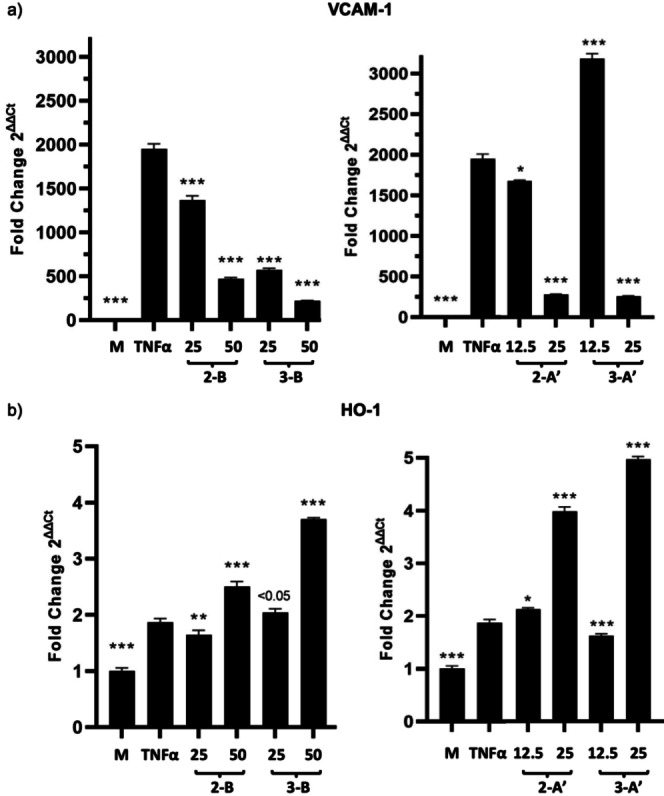
Modulation of VCAM‐1 and HO‐1 mRNA expression by Mito‐CORMs. HUVEC were cultured as described in Figure 10; mRNA expression was assessed by Taqman assays. The results are expressed as mean fold change ± SD with unstimulated HUVEC (normal culture medium) as normalizer. All conditions were tested in triplicate. * *p*<0.01, ** *p*<0.001, *** *p*<0.0001.

We next assessed the anti‐inflammatory properties of Mito‐CORMs by testing their ability to inhibit TNFα‐mediated expression of inflammatory mediators (VCAM‐1, ICAM‐1, CXCL1)^[19]^ and their ability to induce HO‐1,^[20]^ both in a dose‐dependent manner. Similar to what was observed in MTT assays, it was found that the biological effect on inflammatory parameters was more pronounced for Mito‐CORMs **2/3**‐**A**’ as compared to Mito‐CORMs **2/3**‐**B**. This was particularly observed for inhibition of VCAM‐1 expression, while no major difference was observed for HO‐1 between the two types of Mito‐CORMs (Figure 10). In line with the results of the western blot analysis, mRNA expression, assessed by means of quantitative PCR, confirmed the influence of Mito‐CORMs on both VCAM (downregulation) and HO‐1 (upregulation; Figure 11). The anti‐inflammatory effect of Mito‐CORMs was not restricted to VCAM‐1, but was also observed for ICAM‐1 and CXCL1 in the mRNA expression analysis (Table S1 in the Supporting Information).

**Figure 12:**
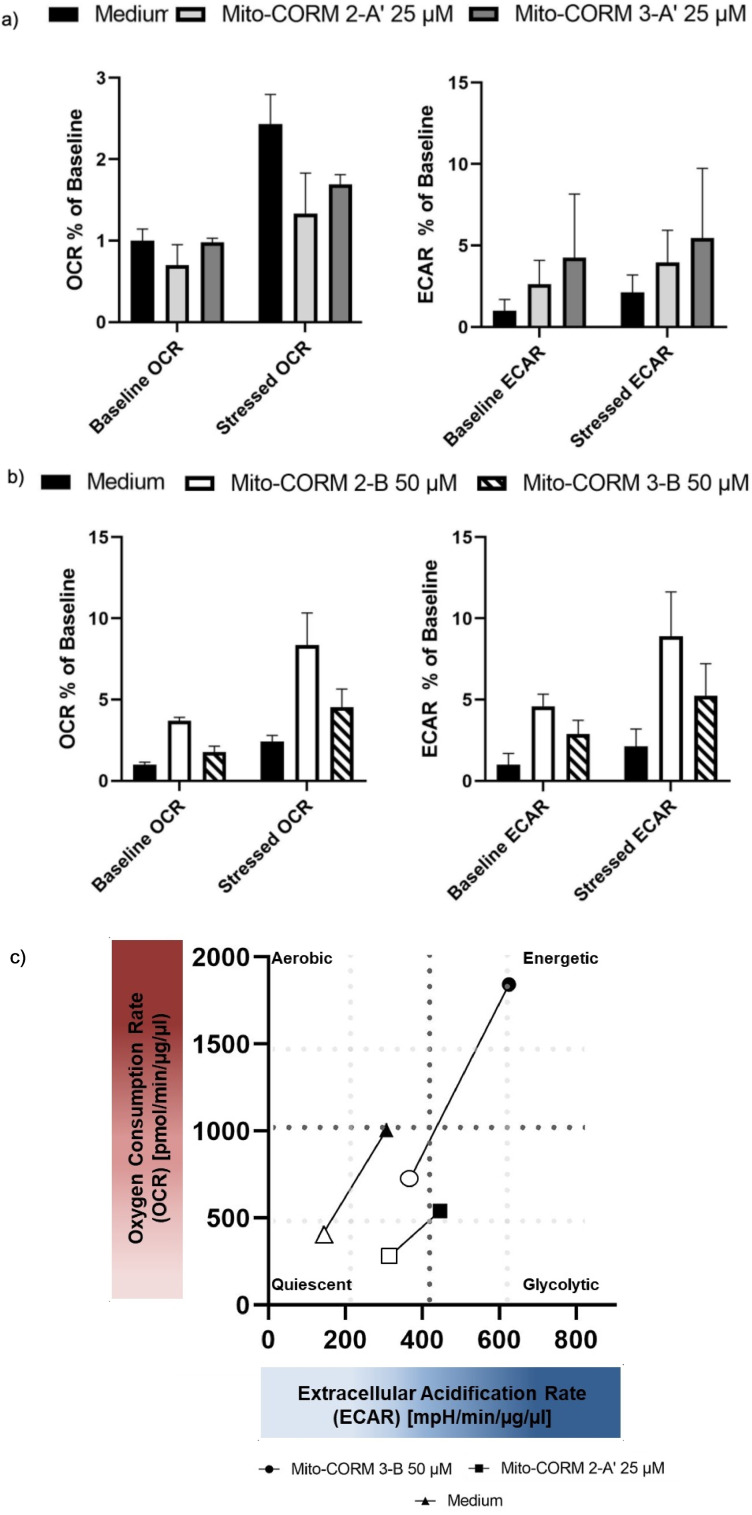
Energy phenotyping of HUVEC exposed to Mito‐CORMs by means of seahorse real‐time cell metabolic analysis. HUVECs were cultured in 96‐well plates overnight in the presence of a) Mito‐CORMs **2/3**‐**A**’ or b) Mito‐CORM **2/3**‐**B**. c) Overview of metabolic effects observed. OCR and ECAR were assessed under basal (open symbols) and stressed (closed symbols) conditions.

To assess whether the two types of Mito‐CORMs affect mitochondrial respiration and glycolysis differently, energy phenotyping was performed using a seahorse real‐time cell metabolic analysis. Using different maximal concentrations in a nontoxic range for Mito‐CORMs **2A**’**/3A**’ and **2B/3B**, oxygen consumption rate (OCR) and extracellular acidification rate (ECAR) were measured as indicators of mitochondrial respiration and glycolysis, respectively, under base‐line conditions and under stress conditions, that is, after addition of oligomycin and FCCP. Although mitochondrial respiration was partly inhibited by Mito‐CORMs **2/3**‐**A**’ under both basal and stressed conditions, glycolysis was increased. For Mito‐CORMs **2/3**‐**B**, mitochondrial respiration and glycolysis were increased in parallel. The metabolic potential of HUVEC treated with Mito‐CORM **3**‐**B** was not significantly different from untreated HUVEC (medium), while the metabolic potential of HUVEC treated with Mito‐CORM **2**‐**A**’ was significantly changed in favor of glycolysis (Figure 12, Table 1).

**Table 1:** Metabolic potential of HUVECs treated with Mito‐CORMs.

Treatment (compound)	*c* [μM]	Metabolic potential^[a]^	Fc^[b]^	*p* value^[c]^
medium		3.73±0.60	1.00±0.16	
MC 2‐A’	12.5	3.84±0.45	1.03±0.12	ns
MC 2‐A’	25.0	1.95±0.52	0.52±0.14	0.02
MC 3‐A’	12.5	3.74±1.25	1.00±0.33	ns
MC 3‐A’	25.0	2.21±0.57	0.59±0.15	0.04
MC 2‐B	25.0	4.45±0.89	1.19±0.24	ns
MC 2‐B	50.0	3.87±0.87	1.04±0.23	ns
MC 3‐B	25.0	3.43±0.36	0.92±0.10	ns
MC 3‐B	50.0	4.41±0.43	1.18±0.11	ns

[a] ▵OCR/▵ECAR. [b] Fold change compared to medium. [c] *P* value compared to medium; ns=not significant.

## Discussion

Most of the CORMs developed during the past 20 years represent transition metal carbonyl complexes (based on Ru, Mn, Fe, etc.) which release their CO load either in a more or less uncontrolled fashion upon dissolution or triggered by light irradiation.^[3]^ In contrast, the ET‐CORMs developed in our laboratory are enzyme‐triggered, and due to their modular architecture facilitate functional fine‐tuning by 1) selecting the enzyme‐cleavable functional group (ester, amide, phosphate), 2) modification of the cyclohexadiene‐Fe(CO)_3_ moiety, and 3) variation of the ester side chain.^[7]^ While we had shown in the past that esterase‐activated ET‐CORMs allow intracellular CO‐release, the present study was performed to address the question whether such compounds can even be rendered mitochondria‐selective in order to deliver CO in this particular subcellular compartment. For this purpose, we designed and synthesized a set of Mito‐CORMs which are characterized by a pyridinium salt unit in the ester side chain. Several studies have demonstrated that such salts rapidly accumulate in mitochondria^[17]^ and, therefore, we have good reason to assume that the compounds used in the present study liberate CO within the mitochondria, even we so far have no proof for this by a direct method such as subcellular CO imaging.

The Mito‐CORMs clearly induce cellular responses resulting from CO release as reflected, for instance, by VCAM inhibition at concentrations ≥25 μM. Thus, their potency on a first glance appears to be comparable to earlier ET‐CORMs.^[7]^ It is noteworthy that the A’‐type Mito‐CORMs (derived from isophorone) display significantly higher toxicity, although the B‐series compounds release almost twice as much CO under noncellular conditions in vitro (3 vs. 1.5 equiv.). To understand this observation, two aspects need to be considered: First, we cannot exclude rapid intracellular CO release from Mito‐CORMs **2/3**‐**A**’ once the molecules enter the cell. Second, the A’ variants might be subject to highly specific mitochondrial targeting that could locally cause rapid accumulation of toxic CO concentrations with subsequent inhibition of mitochondrial cytochrome *c* oxidase, thus explaining the pronounced cytotoxicity of the isophorone derivatives. We also cannot exclude that the combination of CO and hydrolysis products are mediating toxicity and that quantitative and qualitative differences herein explains the higher toxicity of the A’‐type Mito‐CORMS. Nonetheless, in agreement with our earlier studies,[^7d^, ^7e^, ^7g^] we assume the rate of CO liberation in living cells is the pivotal criterion for toxicity. However, another possible explanation would be that Mito‐CORMs **2/3**‐**B** act in the opposite way, which would mean that the B variants release CO more slowly in living cells. This would fit with the observation that these compounds do not inhibit mitochondrial respiration, as was observed with the A′ variants, but rather enhance it. Interestingly, for both Mito‐CORMs **2/3**‐**B** and Mito‐CORMs **2/3**‐**A′**, there were no significant differences between the *meta*‐ or *para*‐substituted pyridinium isomers in terms of toxicity. On the other hand, for both Mito‐CORM variants (A’ and B), our biological data indicate a clear effect on mitochondrial respiration. Whether this is due to direct CO release within mitochondria warrants further studies. Yet our findings that the A’‐ and B‐type Mito‐CORMS have different effects on mitochondrial function are highly interesting and offer means of metabolic reprogramming. Metabolic reprogramming in macrophages has clearly been linked to inflammatory/inflammatory resolution pathways.^[23]^ Likewise, endothelial cells undergo metabolic reprogramming in response to various environmental cues. Hence, the A’‐ and B‐type Mito‐CORMs might counteract/prevent metabolic shifts that otherwise would occur under different pathological conditions. In comparison to other ET‐CORMs, the biological activity of mitochondria targeted CORMs seems to be conserved with respect to their anti‐inflammatory properties (inhibition of VCAM‐1, induction of HO‐1). Yet, it still needs to be addressed if mitochondrial function is more selectively changed by mitochondria targeted CORMs compared to other (ET‐)CORMs.

## Conclusion

In summary, we have designed, synthesized, and characterized a new class of esterase‐triggered CORMs (ET‐CORMs) that carry an ionic moiety in the ester side chain to selectively target mitochondria. Although the anti‐inflammatory properties of the Mito‐CORMs (such as induction of HO‐1 or inhibition of VCAM) are similar to other ET‐CORMs, their water solubility is a major advantage of these new reagents. As Mito‐CORMs allow safe delivery of carbon monoxide to the cell, in vivo experiments would be needed to determine their therapeutic value with respect to inflammatory diseases, possibly after further fine‐tuning of the structures to improve stability, esterase specificity, and tissue selectivity.

## Experimental Section

**Typical procedure: Synthesis of Mito‐CORM 2‐B**: To a solution of TIPS complex *rac*‐**1‐B** (200 mg, 0.51 mmol, 1.0 equiv.) in C$H_{2}{\mathrm{Cl}}_{2}$ (5.0 mL) at room temperature was added a solution of TBAF (1 m in THF, 0.52 mL, 0.52 mmol, 1.0 equiv.), and the mixture was stirred for 10 min at room temperature. Then pyridine (0.14 mL, 1.68 mmol, 3.3 equiv.) was added, and the mixture was stirred for 10 min at room temperature before EDC ⋅ HCl (215 mg, 1.12 mmol, 2.2 equiv.), DMAP (19.0 mg, 0.15 mmol, 0.3 equiv.) and 3‐(3‐pyridinyl)propionic acid (85 mg, 0.28 mmol, 1.1 equiv.) were added successively. The mixture was stirred for 3 h at room temperature. Afterwards, the mixture was diluted with CH_2_Cl_2_ (20 mL) washed with saturated aqueous NaHCO_3_ (5 mL), water (5 mL) and brine (5 mL). The organic phase was dried with MgSO_4_ and the solvent was removed under reduced pressure. The crude product was purified by column chromatography (ultra pure SiO_2_, cHex/EtOAc=1 : 1) to yield 1‐(3‐(3‐pyridyl)propanoyloxy)cyclohexadiene‐Fe(CO)_3_ (175 mg, 0.47 mmol, 93 %) as a yellow oil. To a solution of the obtained complex (126 mg, 0.341 mmol, 1.0 equiv.) in anhydrous Et_2_O (3.5 mL) was added MeOTf (74.7 μL, 0.682 mmol, 2.0 equiv.) dropwise. The mixture was stirred for 30 min at room temperature before the solid product was filtered off and washed with anhydrous Et_2_O (30 mL) to give Mito‐CORM **2‐B** (180 mg, 0.337 mmol, 99 %) as a yellow solid. ^1^H NMR (500 MHz, CDCl_3_): *δ*=8.83 (s, 1H), 8.73 (d, ^3^ *J*=6.0 Hz, 1H), 8.33 (d, ^3^ *J*=8.0 Hz, 1H), 7.90 (dd, ^3^ *J*=8.0, 6.0 Hz, 1H), 5.35 (d, ^3^ *J*=4.4 Hz, 1H), 5.11 (dd, ^3^ *J*=6.5, 4.6 Hz, 1H), 4.44 (s, 3H), 3.16 (t, ^3^ *J*=7.0 Hz, 2H), 3.13–3.09 (m, 1H), 2.83–2.79 (m, 2H), 2.12 (t, ^3^ *J*=11.3 Hz, 1H), 1.84 (td, ^3^ *J*=11.4, 10.8, 3.5 Hz, 1H), 1.73–1.60 (m, 2H); ^13^C NMR (125 MHz, CDCl_3_): *δ*=211.4 (Fe(CO${)}_{3}$ ), 170.1, 145.6, 145.5, 143.4, 142.4, 127.8, 121.0 (d, ^1^ *J*_C‐F_=322 Hz), 103.4, 81.2, 80.0, 61.0, 48.8, 34.0, 27.5, 26.6, 24.1; ^19^F NMR (471 MHz, [D_6_]DMSO): *δ*=−77.8 (s); FTIR (ATR) $\tilde{v}$ [cm^−1^]=2044 (s), 1957 (s), 1754 (m), 1150 (s), 1029 (s), 1005 (m), 637 (s), 610 (s), 560 (s), 516 (s); HRMS (ESI) calcd: [*M*−CF_3_SO_3_]^+^=384.0529 amu, found=384.0526 amu.

**Determination of the CO release by headspace GC**: A ThermoScientific Trace 1300 headspace gas chromatograph equipped with a TriPlus RSH autosampler, a thermal conductivity detector (TCD) and a Shin carbon ST 100/120 column (1.0 mm×2 m; 1/16” OD silico) was used. 10 mL vials closed with gas‐tight silicon/PTFE septa were charged with the respective complex (36 μmol), DMSO (0.2 mL) and phosphate buffer (1 mL, 0.1 M, pH 7.4). After the vials had been equilibrated for 10 min at 37 °C, PLE (15 mg) was added, and the amount of CO accumulating in the gas space was assessed through headspace GC using a previously recorded calibration curve. As a control, samples without addition of esterase were analyzed in the same manner.

Umbilical cords were obtained from healthy women at the Department of Obstetrics, University Medical Center Mannheim, after written informed consent. Isolation was approved by the local ethics committee (Medizinische Ethikkommission II der Medizinischen Fakultät Mannheim Ruprechts‐Karls‐University Heidelberg (approval no: 2015‐518‐MA)). All research was performed in accordance with the Declaration of Helsinki and in accordance with relevant guidelines/regulations.

All further experimental details are provided in the Supporting Information.

## Conflict of interest

The authors declare no conflict of interest.

1

## Supporting information

As a service to our authors and readers, this journal provides supporting information supplied by the authors. Such materials are peer reviewed and may be re‐organized for online delivery, but are not copy‐edited or typeset. Technical support issues arising from supporting information (other than missing files) should be addressed to the authors.

Supporting Information

## Data Availability

The data that support the findings of this study are available in the supplementary material of this article.
