# Supplementary material for: Synthesis and Biological Evaluation of Water‐Soluble Esterase‐Activated CO‐Releasing Molecules Targeting Mitochondria
Source: Chemistry. 2022 Jul 14;28(50):e202201670. doi: 10.1002/chem.202201670 (PMC9543658; doi:10.1002/chem.202201670)
Supplement: Supplementary file 1 — Supporting Information [file CHEM-28-0-s001.pdf]

Chemistry–A European Journal

Supporting Information

Synthesis and Biological Evaluation of Water-Soluble Esterase-Activated CO-Releasing Molecules Targeting Mitochondria

Lars Hemmersbach, Yannick Schreiner, Xinmiao Zhang, Finn Dicke, Leon Hünemeyer, Jörg-Martin Neudörfl, Thomas Fleming, Benito Yard,* and Hans-Günther Schmalz*

Table of Contents

1. Experimental 1

1.1. General Information 1

1.2. Syntheses 2

1.2.1. (1,5-Cyclohexadiene-$Fe(CO)_3$)-1-yl isonicotinate (*rac*-2-A) 2

1.2.2. Mito-CORM 1-A 3

1.2.3. (1,3-Cyclohexadiene-$Fe(CO)_3$)-1-yl isonicotinate (*rac*-2-B) 5

1.2.4. Mito-CORM 1-B 6

1.2.5. (1,5-Cyclohexadiene-$Fe(CO)_3$)-1-yl-3-pyridine propionate (*rac*-6-A) 8

1.2.6. Mito-CORM 2-A 9

1.2.7. (1,3-Cyclohexadiene-$Fe(CO)_3$)-1-yl-3-pyridine propionate (*rac*-6-B) 10

1.2.8. Mito-CORM 2-B 12

1.2.9. (1,5-Cyclohexadiene-$Fe(CO)_3$)-1-yl-4-pyridine propionate (*rac*-8-A) 13

1.2.10. Mito-CORM 3-A 15

1.2.11. (1,3-Cyclohexadiene-$Fe(CO)_3$)-1-yl-4-pyridine propionate (*rac*-8-B) 16

1.2.12. Mito-CORM 3-B 17

1.2.13. 3,3,5-Trimethylcyclohexa-1,5-diene-1-yl acetate (10) 19

1.2.14. (3,3,5-Trimethylcyclohexa-1,5-diene-$Fe(CO)_3$)-1-yl acetate (rac-11-A’) and (3,3,5-trimethylcyclohexa-1,3-diene-$Fe(CO)_3$)-1-yl acetate (rac-11-B’) 20

1.2.15. 1-Tri*iso*propylsiloxy-3,3,5-trimethyl-1,5-cyclohexadiene-($Fe(CO)_3$) (rac-12-A’) 22

1.2.16. (3,3,5-Trimethylcyclohexa-1,5-diene-$Fe(CO)_3$)-1-yl-3-pyridine propionate (rac-13-A’) 23

1.2.17. Mito-CORM 2-A’ 25

1.2.18. (3,3,5-Trimethylcyclohexa-1,5-diene-$Fe(CO)_3$)-1-yl-4-pyridine propionate (*rac*-14-A’) 26

1.2.19. Mito-CORM 3-A’ 28

1.2.20. (*E*)-Methyl 3-(pyridine-4-yl)acrylate (16) 29

1.2.21. (*rac*)-Methyl 2-(pyridine-4-yl)cyclopropanecarboxylate (*rac*-17) 30

1.2.22. (*rac*)-Cyclopropanecarboxylic acid, 2-(4-pyridinyl)-hydrochloride (*rac*-18) 31

1.2.23. Complex *ambo-3-B* 32

1.2.24. Mito-CORM 4-B 34

1.2.25. Methyl 3-(pyridine-3-yl)propanoate (19) 35

1.2.26. [Methyl 3-(*N*-methylpyridinium-3-yl)propanoate] triflate (LHP545) 36

1.2.27. Methyl 3-(pyridine-4-yl)propanoate (20) 37

1.2.28. [Methyl 3-(N-methylpyridinium-4-yl)propanoate] triflate (LHP551) 38

NMR Spectra 40

2. In situ quantification of CO release 71
3. Crystallographic details 72
3.1. Crystal data and structure refinement for 1,5-Cyclohexadien-Fe$(CO)_3$)-1-yl isonicotinate (*rac*-2-A) 72
3.2. Crystal data and structure refinement for Mito-CORM 1-A........ 73
3.3. Crystal data and structure refinement for Mito-CORM 1-B........ 74
3.4. Crystal data and structure refinement for *rac*-18 75
3.5. Crystal data and structure refinement for Mito-CORM 2-B........ 76
4. Biological Procedures 77
4.1. Reagents........ 77
4.2. Cell culture and viability assays........ 78
4.3. Protein isolation and Western Blotting........ 79
4.4. RNA isolation, cDNA synthesis and qPCR........ 79
4.5. Seahorse metabolic analysis 80
5. References........ 81

1. Experimental

1.1. General Information

Unless otherwise specified, all reactions were carried out under inert conditions. Glassware was heat-dried under vacuum and flushed with argon (*Linde*® Argon 4.6 (99.996 %, <1 ppm water, <1 ppm oxygen). Solids were added under argon counter flow. Liquids were added with syringes through gas-tight septa. Before use, syringes were flushed with argon thrice.

Reagents were purchased from commercial suppliers (*Sigma-Aldrich*, *Lancaster*, *Alfa Aesar*, *Carbolution*, *ABCR* or *Acros*) and used without further purification unless otherwise noted. Tetrahydrofuran, diethyl ether and toluene were distilled from sodium and benzophenone. Dichloromethane was distilled from calcium hydride. 2-Cyclohexenone and DIPA were distilled and stored under argon.

All reactions were monitored by TLC using 0.25 mm *Merck* silica gel 60 F254 precoated plates and visualized using with UV light (*Mineralight* UVGL-25 lamp) or by staining with a potassium permanganate solution (3 g $KMnO_4$, 5 mL NaOH (5 Vol.-%) in 300 mL H_2O) followed by heating. Flash column chromatography was conducted with silica gel from *Acros* (60 Å, 0.035 nm – 0.070 nm). All iron carbonyl complexes were purified using ultra pure silica gel supplied by *Acros* (60 Å, 0.040 nm – 0.060 nm).

^{1}H and ^{13}C NMR spectra were recorded on *Bruker* instruments: Avance II 300 MHz (^{1}H NMR: 300 MHz; ^{13}C NMR: 75 MHz), Avance AVIII-HD 500 (^{1}H NMR: 500 MHz; ^{13}C NMR: 125 MHz), or Avance II 600 (^{1}H NMR: 600 MHz; 150 MHz). Chemical shifts are given in ppm (parts per million). Coupling constants are reported in Hz. Multiplicities are specified using the following abbreviations: s (singlet), d (doublet), t (triplet), q (quartet), m (multiplet) and combinations of these abbreviations.

IR spectra were recorded at room temperature on an UATR TWO FT-IR-spectrometer from *Perkin Elmer*. Wavenumbers ῦ [cm^{-1}] and relative intensities of selected signals are listed using the following abbreviations: s (strong), m (medium), w (weak).

GC-MS spectra were recorded on an *Agilent* HP6890 system with a (MSD)5937N mass detector. A *Macherey-Nagel*-1-MS (30 m x 0.25 mm) capillary column was used. Hydrogen with a flow of 30 mL/min was utilized as carrier gas using the following temperature program (50-300M) at a gas stream of 10 psi: 50 °C (2 min), 50 – 300 °C (21 °C/min), 300 °C (5 min). The relative intensity of the peaks as percentage of the most intense signal (100%).

HR-ESI-MS spectra were measured on a THERMO Scientific LTQ Orbitrap XL-FTMS Analyser using electrospray ionization (3.4 kV spray voltage). The capillary and tube lens voltage were set to 3.0 V.

HR-EI spectra were recorded on a *Thermo Scientific Exative GC* instrument.

Melting points were measured using a *Büchi* B-545 system with a heating rate of 2 °C min^{-1}.

1.2. Syntheses

TIPS-complexes *rac*-**1-A** and **B** were synthesized according to a previously published procedure.[1]

1.2.1. (1,5-Cyclohexadiene-Fe(CO)$_3$)-1-yl isonicotinate (*rac*-2-A)

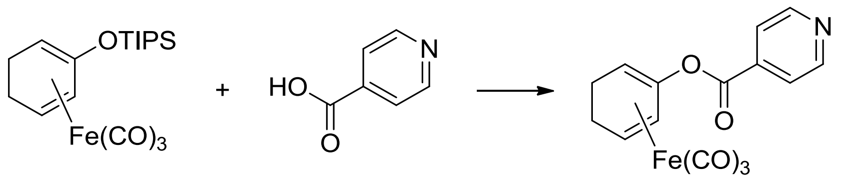

rac-**1-A** **4** *rac*-**2-A**

To a solution of TIPS-complex *rac*-**1-A** (201 mg, 0.51 mmol, 1.0 eq.) in THF (5.0 mL) at room temp was added a solution of TBAF (1 M in THF, 0.51 mL, 0.51 mmol, 1.0 eq.) and the mixture was stirred for 10 min at room temp. Then pyridine (0.13 mL, 1.53 mmol, 3.0 eq.) was added and the mixture was stirred for 10 min at room temp before EDC*HCl (215 mg, 1.12 mmol, 2.2 eq.), DMAP (19.1 mg, 0.15 mmol, 0.3 eq.) and acid **4** (69 mg, 0.56 mmol, 1.1 eq.) were added successively. The mixture was stirred for 18 h at room temp and was diluted with EtOAc (15 mL). The mixture was washed with saturated aqueous $NaHCO_3$ (5 mL), water (5 mL) and brine (5 mL). The organic phase was dried with $MgSO_4$ and the solvent was removed under reduced pressure. The crude product was purified by column chromatography (ultra pure SiO_2, cHex/EtOAc = 5:1) to yield *rac*-**2-A** (114 mg, 0.33 mmol, 66%) as a yellow solid.

M($C_{15}H_{11}FeNO_5$): 341.10 g mol^{-1}.

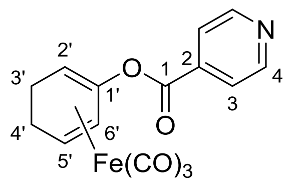

Melting Point: 85 – 86 °C (EtOAc).

TLC: R_f (EtOAc) = 0.73.

**^{1}H NMR:**	(500 MHz, $CDCl_3$) δ = 8.83 (d, ^3J = 5.1 Hz, 2H, H-4), 7.86 (d, ^3J = 5.4 Hz, 2H, H-3), 5.65 (dd, ^3J = 6.7, ^4J = 2.1 Hz, 1H, H-6‘), 3.48 (dt, ^3J = 4.2, ^4J = 2.3 Hz, 1H, H-2‘), 2.93 (dt, ^3J = 6.3, ^4J = 3.0 Hz, 1H, H-5‘), 1.93 – 1.87 (m, 1H, H-3‘), 1.84 – 1.77 (m, 1H, H-3‘), 1.65 – 1.49 (m, 2H, H-4‘).
**^{13}C NMR:**	(125 MHz, $CDCl_3$): δ = 210.6 ($Fe(CO)_3$), 164.7 (C-1), 151.0 (C-3), 136.1 (C-2), 128.6 (C-1’), 123.0 (C-3), 80.1 (C-6), 59.0 (C-2), 52.4 (C-5), 24.7 (C-3’), 23.6 (C-4’).
**LR-MS (ESI):**	$[M+H]^+$ = 341.9 amu.
**HR-MS (ESI):**	Calc..: $[M+H]^+$ = 342.0059 amu, found. = 342.0058 amu.
**FT-IR:**	(ATR) ṽ [cm^{-1}] = 2950 (w), 2933 (w), 2856 (w), 2045 (s), 1961 (s); 1748 (m), 1560 (w), 1564 (w), 1456 (w), 1430 (w), 1409 (w), 1325 (w), 1268 (m), 1168 (m), 1120 (w), 1086 (w), 1063 (w), 1023 (w), 992 (w), 926 (w), 846 (w), 807 (w), 752 (w), 703 (w), 641 (w), 619 (m), 608 (m), 572 (m), 513 (w), 461 (w).

1.2.2. Mito-CORM 1-A

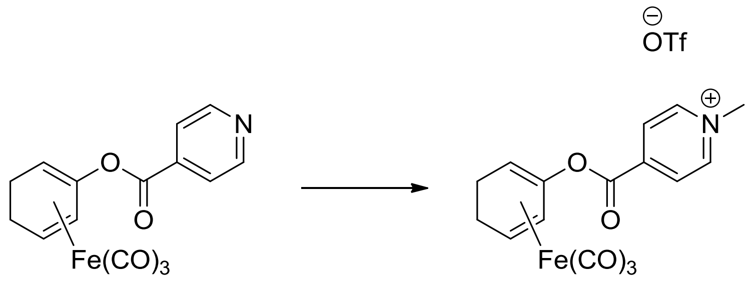

rac-**2-A** **Mito-CORM 1-A**

To a solution of complex *rac*-**2-A** (103 mg, 0.30 mmol, 1.0 eq.) in absolute Et_2O (3.0 mL) was added MeOTf (34.2 µL, 0.30 mmol, 1.0 eq.) dropwise. The mixture was stirred for 30 min at room temp, the formed solid was filtered off and washed with absolute Et_2O (20 mL). **Mito-CORM 1-A** (131 mg, 0.26 mmol, 86%) was obtained as a yellow solid.

M($C_{17}H_{13}F_3FeNO_8S$): 504.19 g mol^{-1}.

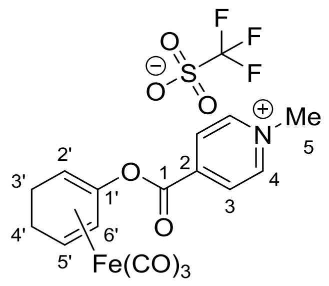

Melting Point: 112 – 113 °C (Et_2O).

^{1}H NMR: (500 MHz, DMSO-d$_6$) δ = 9.20 (d, ^3J = 6.3 Hz, 2H, H-4), 8.50 (d, ^3J = 6.2 Hz, 2H, H-3), 6.06 (dd, ^3J = 6.8, ^4J = 2.0 Hz, 1H, H-6‘), 4.45 (s, 3H, H-5), 3.79 (dt, ^3J = 4.4, ^4J = 2.4 Hz, 1H, H-2‘), 3.13 (dt, ^3J = 6.3, 2.9 Hz, 1H, H-5‘), 1.88 – 1.72 (m, 2H, H-3‘), 1.65 – 1.45 (m, 2H, H-4‘).

^{13}C NMR: (125 MHz, DMSO-d$_6$): δ = 210.7 (Fe(CO)$_3$), 161.5 (C-1), 147.2, (C-4), 142.4 (C-2), 128.1 (C-1'), 126.8 (C-3), 120.6 (q, $^1J_{C\text{-}F}$ = 320 Hz, CF_3) , 80.6 (C-6'), 58.9 (C-2'), 53.4 (C-5'), 48.5 (C-5), 24.1 (C-3'), 23.0 (C-4').

^{19}F NMR: (471 MHz, DMSO-d_6) δ = -77.8 (s).

LR-MS (ESI): [M-CF_3SO_3]$^+$ = 356.0 amu.

HR-MS (ESI): Calc.: [M-CF_3SO_3]$^+$ = 356.0216 amu, found. = 356.0220 amu.

FT-IR: (ATR) ṽ [cm^{-1}] = 3134 (w), 3091 (w), 3063 (w), 2988 (w), 2954 (w), 2925 (w), 2853 (w), 2800 (w), 2049 (s), 1954 (s), 1759 (m), 1646 (w), 1583 (w), 1456 (w), 1430 (w), 1393 (w), 1338 (w), 1262 (s), 1225 (s), 1160 (s), 1119 (m), 1096 (m), 1057 (m), 1030 (s), 996 (w), 927 (w), 868 (m), 814 (w), 781 (w), 758 (m), 675 (m), 635 (s), 617 (s), 603 (s), 570 (s), 516 (s), 478 (m), 458 (m).

1.2.3. (1,3-Cyclohexadiene-Fe(CO)3)-1-yl isonicotinate (*rac*-2-B)

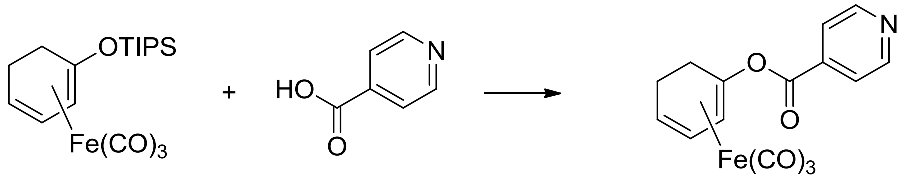

rac-**1-B** **4** *rac*-**2-B**

To a solution of TIPS-complex *rac*-**1-B** (100 mg, 0.25 mmol, 1.0 eq.) in CH_2Cl_2 (2.5 mL) at room temp was added a solution of TBAF (1 M in THF, 0.26 mL, 0.26 mmol, 1.0 eq.) and the mixture was stirred for 10 min at room temp. Then pyridine (67 µL, 0.84 mmol, 3.3 eq.) was added and the mixture was stirred for 10 min at room temp before EDC*HCl (108 mg, 0.56 mmol, 2.2 eq.), DMAP (10.0 mg, 0.076 mmol, 0.3 eq.) and acid **4** (35 mg, 0.28 mmol, 1.1 eq.) were added successively. The mixture was stirred for 17 h at room temp. Afterwards, the mixture was diluted with CH_2Cl_2 (10 mL) washed with saturated aqueous $NaHCO_3$ (2.5 mL), water (2.5 mL) and brine (5 mL). The organic phase was dried with $MgSO_4$ and the solvent was removed under reduced pressure. The crude product was purified by column chromatography (ultra pure SiO_2, cHex/EtOAc = 2:1) to yield *rac*-**2-B** (64 mg, 0.19 mmol, 74%) as a yellow solid.

M($C_{15}H_{11}FeNO_5$): 341.10 g mol^{-1}.

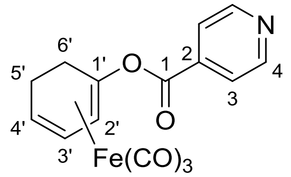

Melting Point: 113 – 114 °C (EtOAc).

TLC: R_f (cHex/EtOAc = 2:1) = 0.27.

^{1}H NMR: (500 MHz, $CDCl_3$) δ = 8.79 (d, ^3J = 5.2 Hz, 2H, H-4), 7.84 (d, ^3J = 6.0 Hz, 1H, H-3), 5.53 (dd, ^3J = 4.3, ^4J = 2.2 Hz, 1H, H-2‘), 5.18 (dd, ^3J = 6.4, 4.7 Hz, 1H, H-3‘), 3.25 – 3.08 (m, 1H, H-4‘), 2.37 – 2.18 (m, 1H, H-6‘), 1.98 – 1.88 (m, 2H, H-5‘, H-6‘), 1.84 – 1.63 (m, 1H, H-5‘).

^{13}C NMR: (125 MHz, $CDCl_3$): δ = 211.1 ($Fe(CO)_3$), 163.6 (C-1), 150.8 (C-4), 137.2 (C-2), 122.9 (C-3), 103.7 (C-1'), 81.2 (C-3'), 80.1 (C-2'), 60.9 (C-4'), 26.8 (C-6'), 24.4 (C-5').

LR-MS (ESI): $[M+H]^+$ = 341.9 amu.

HR-MS (ESI): Calc.: $[M+H]^+$ = 342.0059 amu, found. = 342.0058 amu.

FT-IR: (ATR) ṽ [cm^{-1}] = 3058 (w), 3036 (w), 2947 (w), 2858 (w), 2047 (s), 1963 (s), 1735 (m), 1596 (w), 1563 (w), 1470 (w), 1445 (w), 1408 (w), 1383 (w), 1324 (w), 1276 (m), 1261 (m), 1213 (w), 1182 (w), 1122 (m), 1080 (w), 1064 (w), 1004 (w), 958 (w), 913 (w), 849 (w), 781 (w), 754 (w), 705 (w), 678 (w), 640 (w), 612 (m), 560 (m), 538 (w), 516 (w), 492 (w).

1.2.4. Mito-CORM 1-B

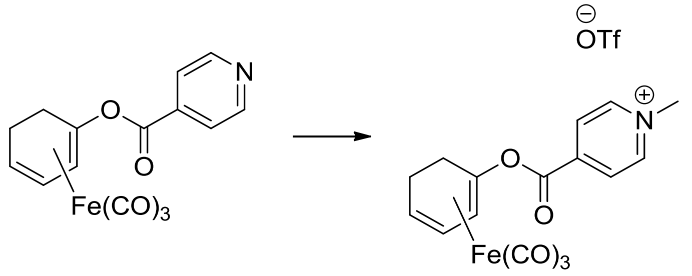

rac-**2-B** **Mito-CORM 1-B**

To a solution of complex *rac*-**2-B** (50.0 mg, 0.146 mmol, 1.0 eq.) in absolute Et_2O (2.5 mL) was added MeOTf (23.1 µL, 0.293 mmol, 2.0 eq.) dropwise. The mixture was stirred for 30 min at room temp, the formed solid was filtered off and washed with absolute Et_2O (20 mL). **Mito-CORM 1-B** (61.0 mg, 0.121 mmol, 82%) was obtained as a yellow solid.

M($C_{17}H_{13}F_3FeNO_8S$): 504.19 g mol^{-1}.

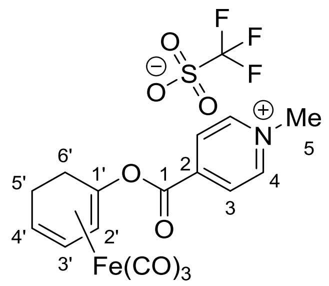

Melting Point: 171 – 172 °C (Et_2O).

^{1}H NMR: (500 MHz, DMSO-d_6) δ = 9.20 (d, ^3J = 6.4 Hz, 2H, H-4), 8.56 (d, ^3J = 6.7 Hz, 2H, H-3), 6.00 (d, ^3J = 4.6 Hz, 1H, H-2‘), 5.54 (dd, ^3J = 5.5 Hz, 1H, H-3‘), 4.42 (s, 3H, H-5), 3.40 – 3.35 (m, 1H, H-4‘), 2.31 – 2.21 (m, 1H, H-6‘), 1.94 – 1.82 (m, 2H, H-5‘, H-6‘), 1.77 – 1.68 (m, 1H, H-5‘).

^{13}C NMR: (125 MHz, DMSO-d_6): δ = 211.5 ($Fe(CO)_3$), 160.5 (C-1), 147.3 (C-4), 142.9 (C-2), 126.7 (C-3), 120.7 (q, $^1J_{C\text{-}F}$ = 322 Hz, CF_3), 104.4 (C-1’), 82.1 (C-3’), 79.9 (C-2’), 62.4 (C-4’), 48.5 (C-5), 26.0 (C-6’), 23.7 (C-5’).

^{19}F NMR: (471 MHz, DMSO-d_6) δ = -77.8 (s).

LR-MS (ESI): $[M\text{-}CF_3SO_3]^+$ = 356.0 amu.

HR-MS (ESI): Calc.: $[M\text{-}CF_3SO_3]^+$ = 356.0216 amu, found. = 356.0215 amu.

FT-IR: (ATR) ῡ [cm^{-1}] = 3136 (w), 3091 (w), 3069 (w), 2964 (w), 2936 (w). 2861 (w), 2051 (s), 1966 (s), 1742 (w), 1647 (w), 1583 (w), 1472 (w), 1326 (w), 1259 (s), 1224 (m), 1186 (w), 1143 (m), 1124 (m), 1035 (m), 1004 (w), 904 (w), 867 (w), 802 (w), 762 (w), 680 (w), 640 (m), 611 (s), 565 (m), 516 (m), 472 (w), 456 (m).

1.2.5. (1,5-Cyclohexadiene-Fe(CO)3)-1-yl-3-pyridine propionate (*rac*-6-A)

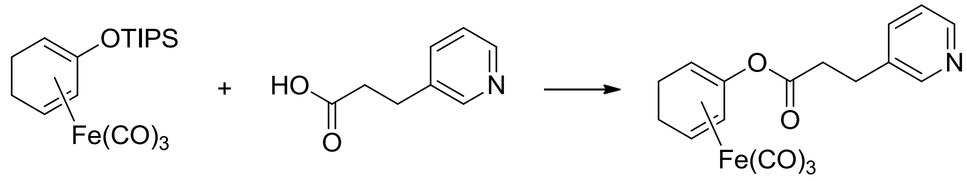

rac-**1-A** **5** *rac*-**6-A**

To a solution of TIPS-complex *rac*-**1-A** (200 mg, 0.51 mmol, 1.0 eq.) in CH_2Cl_2 (5.0 mL) at room temp was added a solution of TBAF (1 M in THF, 0.52 mL, 0.52 mmol, 1.0 eq.) and the mixture was stirred for 10 min at room temp. Then pyridine (0.14 mL, 1.68 mmol, 3.3 eq.) was added and the mixture was stirred for 10 min at room temp before EDC*HCl (215 mg, 1.12 mmol, 2.2 eq.), DMAP (19.0 mg, 0.15 mmol, 0.3 eq.) and acid **5** (85 mg, 0.56 mmol, 1.1 eq.) were added successively. The mixture was stirred for 2 h at room temp. Afterwards, the mixture was diluted with CH_2Cl_2 (20 mL) washed with saturated aqueous $NaHCO_3$ (5 mL), water (5 mL) and brine (5 mL). The organic phase was dried with $MgSO_4$ and the solvent was removed under reduced pressure. The crude product was purified by column chromatography (ultra pure SiO_2, cHex/EtOAc = 1:1) to yield *rac*-**6-A** (123 mg, 0.33 mmol, 65%) as a yellow oil.

M($C_{17}H_{15}FeNO_5$): 369.15 g mol^{-1}.

TLC: R_f (cHex/EtOAc = 2:1) = 0.15.

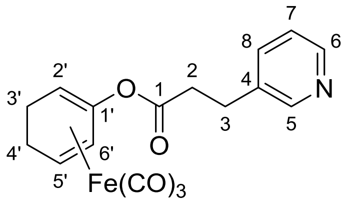

^{1}H NMR: (600 MHz, $CDCl_3$) δ = 8.60 (brs, 2H, H-5, H-6), 7.58 (d, ^3J = 7.8 Hz, 1H, H-8), 7.29 (d, ^3J = 6.8 Hz, 1H, H-7), 5.48 (dd, ^3J = 6.8, ^4J = 2.0 Hz, 1H, H-6‘), 3.36 – 3.25 (m, 1H, H-2‘), 3.03 (t, ^3J = 7.5 Hz, 2H, H-3), 2.88 – 2.85 (m, 1H, H-5‘), 2.81 (t, ^3J = 7.5 Hz, 2H, H-2), 1.89 – 1.80 (m, 1H, H-3‘), 1.75 (ddt, ^2J = 14.5, ^3J = 10.2, 4.1 Hz, 1H, H-3‘), 1.64 – 1.49 (m, 2H, H-4‘).

^{13}C NMR: (150 MHz, $CDCl_3$): δ = 210.7 ($Fe(CO)_3$), 171.7 (C-1), 150.0 (C-5), 148.2 (C-6), 135.9 (C-8), 135.5 (C-4), 128.3 (C-1’), 123.8 (C-7),

79.9 (C-6'), 59.0 (C-2'), 52.2 (C-5'), 35.4 (C-2), 28.0 (C-3), 24.7 (C-3'), 23.6 (C-4').

LR-MS (ESI): $[M+H]^+$ = 370.0 amu.

HR-MS (ESI): Calc.: $[M+H]^+$ = 370.0372 amu, found. = 370.0370 amu.

Calc.: $[M+Na]^+$ = 393.0192 amu, found. = 393.0190 amu.

FT-IR: (ATR) ṽ [cm^{-1}] = 3029 (w), 3007 (w), 2933 (w), 2855 (w), 2043 (s), 1953 (s), 1758 (m), 1576 (w), 1479 (w), 1471 (w), 1457 (m), 1425 (m), 1395 (w), 1366 (w), 1321 (w), 1292 (w), 1230 (w), 1167 (s), 1119 (s), 1075 (m), 1027 (w), 995 (w), 924 (w), 864 (w), 839 (w), 802 (w), 713 (m), 657 (w), 607 (s), 568 (s), 504 (m), 460 (m).

1.2.6. Mito-CORM 2-A

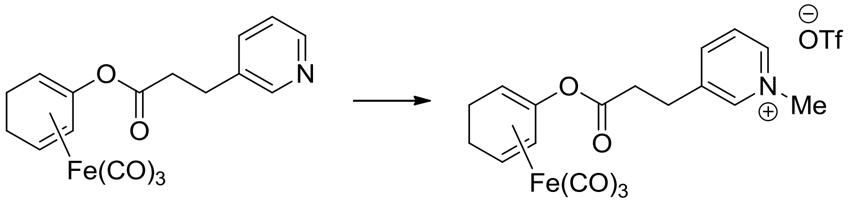

rac-**6-A** **Mito-CORM 2-A**

To a solution of complex *rac*-**6-A** (88.1 mg, 0.238 mmol, 1.0 eq.) in absolute Et_2O (5 mL) was added MeOTf (52.2 µL, 0.477 mmol, 2.0 eq.) dropwise. The mixture was stirred for 30 min at room temp the formed solid was filtered off and washed with absolute Et_2O (20 mL). **Mito-CORM 2-A** (107 mg, 0.20 mmol, 84%) was obtained as a yellow oil.

M($C_{19}H_{18}F_3FeNO_8S$): 533.25 g mol^{-1}.

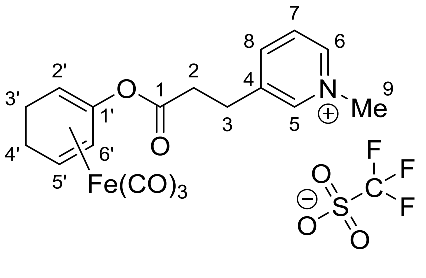

**^{1}H NMR:**	(500 MHz, $CDCl_3$) δ = 8.87 (s, 1H, H-5), 8.73 (d, ^3J = 6.0 Hz, 1H, H-6), 8.35 (d, ^3J = 8.0 Hz, 1H, H-8), 7.93 (t, ^3J = 6.9 Hz, 1H, H-7), 5.52 (dd, ^3J = 6.7, ^4J = 2.0 Hz, 1H, H-6‘), 4.46 (s, 3H, H-9), 3.36 – 3.28 (m, 1H, H-2‘), 3.20 (t, ^3J = 6.9 Hz, 2H, H-3), 2.95 (dt, ^3J = 11.2, 5.2 Hz, 2H, H-2), 2.90 – 2.79 (m, 1H, H-5‘), 1.84 – 1.68 (m, 2H, H-3‘), 1.61 – 1.46 (m, 2H, H-4‘).
**^{13}C NMR:**	(125 MHz, $CDCl_3$): δ = 210.7 ($Fe(CO)_3$), 171.4 (C-1), 145.8 (C-5), 145.5 (C-8), 143.5 (C-6), 142.1 (C-4), 128.2 (C-1’), 127.9 (C-7), 121.8 (d, $^1J_{C\text{-}F}$ = 320 Hz, CF_3), 80.0 (C-6’), 58.7 (C-2’), 52.5 (C-5’), 49.0 (C-9), 33.8 (C-2), 27.4 (C-3), 24.7 (C-3’), 23.5 (C-4’).
**^{19}F NMR:**	(471 MHz, DMSO-d_6) δ = -77.8 (s).
**LR-MS (ESI):**	$[M\text{-}CF_3SO_3]^+$ = 384.0 amu.
**HR-MS (ESI):**	Calc.: $[M\text{-}CF_3SO_3]^+$ = 384.0529 amu, found. = 384.0526 amu.
**FT-IR:**	(ATR) ῡ [cm^{-1}] = 3071 (w), 3014 (w), 2935 (w), 2851 (w), 2045 (s), 1957 (s), 1756 (m), 1639 (w), 1510 (w), 1469 (w), 1458 (w), 1431 (w), 1255 (s), 1224 (m), 1132 (s), 1076 (w), 1029 (s), 992 (w), 924 (w), 864 (w), 811 (w), 755 (m), 682 (m), 637 (s), 610 (s), 569 (s), 516 (s).

1.2.7. (1,3-Cyclohexadiene-Fe(CO)3)-1-yl-3-pyridine propionate (*rac*-6-B)

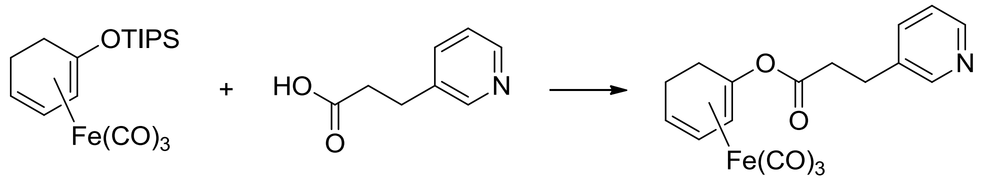

To a solution of TIPS-complex *rac*-**1-B** (200 mg, 0.51 mmol, 1.0 eq.) in CH_2Cl_2 (5.0 mL) at room temp was added a solution of TBAF (1 M in THF, 0.52 mL, 0.52 mmol, 1.0 eq.) and the mixture was stirred for 10 min at room temp. Then pyridine (0.14 mL, 1.68 mmol, 3.3 eq.) was added and the mixture was stirred for 10 min at room temp before EDC*HCl (215 mg, 1.12 mmol, 2.2 eq.),

DMAP (19.0 mg, 0.15 mmol, 0.3 eq.) and acid **5** (85 mg, 0.28 mmol, 1.1 eq.) were added successively. The mixture was stirred for 3 h at room temp. Afterwards, the mixture was diluted with CH_2Cl_2 (20 mL) washed with saturated aqueous $NaHCO_3$ (5 mL), water (5 mL) and brine (5 mL). The organic phase was dried with $MgSO_4$ and the solvent was removed under reduced pressure. The crude product was purified by column chromatography (ultra pure SiO_2, cHex/EtOAc = 1:1) to yield *rac*-**6-B** (175 mg, 0.47 mmol, 93%) as a yellow oil.

M($C_{17}H_{15}FeNO_5$): 369.15 g mol^{-1}.

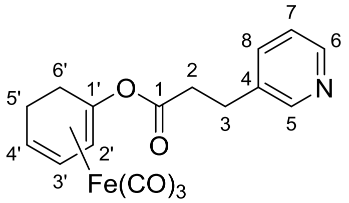

TLC: R_f (cHex/EtOAc = 2:1) = 0.15.

^{1}H NMR: (600 MHz, $CDCl_3$) δ = 8.50 (d, ^4J = 2.3 Hz, 1H, H-5), 8.48 (dd, ^3J = 4.9, ^4J = 1.6 Hz, 1H, H-6), 7.58 – 7.53 (m, 1H, H-8), 7.23 (ddd, ^3J = 7.8, 4.8, ^5J = 0.8 Hz, 1H, H-7), 5.35 (dd, ^3J = 4.5, ^4J = 1.2 Hz, 1H, H-2‘), 5.12 (ddt, ^3J = 6.3, 4.5, ^4J = 0.8 Hz, 1H, H-3‘), 3.11 (dddd, ^3J = 6.3, 3.8, 2.3, ^4J = 1.5 Hz, 1H, H-4‘), 2.98 (td, ^3J = 7.8, 1.9 Hz, 2H, H-3), 2.66 (td, ^3J = 7.8, 1.1 Hz, 2H, H-2), 2.12 (dddd, ^2J = 13.0, ^3J = 11.6, ^4J = 3.0, 1.2 Hz, 1H, H-6‘), 1.87 (ddt, ^2J = 15.4, ^3J = 12.1, 3.3 Hz, 1H, H-5‘), 1.73 (dddd, ^2J = 13.4, ^3J = 8.4, 3.3, 0.9 Hz, 1H, H-6‘), 1.70 – 1.63 (m, 1H, H-5‘).

^{13}C NMR: (150 MHz, $CDCl_3$): δ = 211.3 ($Fe(CO)_3$), 170.7 (C-1), 150.0 (C-5), 148.1 (C-6), 135.9 (C-8), 135.7 (C-4), 123.5 (C-7), 103.4 (C-1’), 80.9 (C-3’), 80.3 (C-2’), 60.6 (C-4’), 35.7 (C-2), 28.1 (C-3), 26.8 (C-6’), 24.2 (C-5’).

LR-MS (ESI): $[M+H]^+$ = 370.0 amu.

HR-MS (ESI): Calc.: $[M+H]^+$ = 370.0372 amu, found. = 370.0366 amu.

Calc.: $[M+Na]^+$ = 392.0192 amu, found. = 392.0187 amu.

FT-IR: (ATR) ῡ [cm^{-1}] = 3055 (w), 3026 (w), 2937 (w), 2894 (w), 2858 (w), 2044 (s), 1958 (s), 1747 (m), 1576 (w), 1479 (w), 1425 (w), 1368 (w), 1328 (w), 1294 (w), 1231 (w), 1182 (m), 1135 (m), 1110 (m), 1068 (w), 1028 (w), 1005 (w), 900 (w), 802 (w), 753 (w), 714 (w), 612 (s), 561 (m), 514 (w), 490 (w).

1.2.8. Mito-CORM 2-B

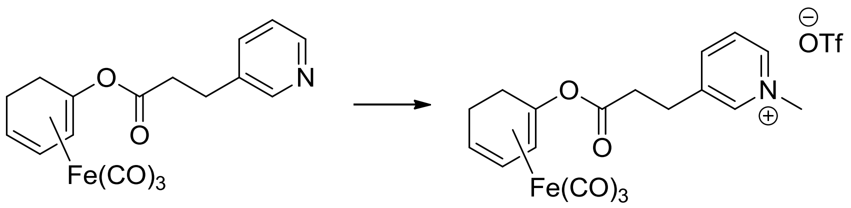

rac-**6-B** **Mito-CORM 2-B**

To a solution of complex *rac*-**6-B** (126 mg, 0.341 mmol, 1.0 eq.) in absolute Et_2O (3.5 mL) was added MeOTf (74.7 µL, 0.682 mmol, 2.0 eq.) dropwise. The mixture was stirred for 30 min at room temp the formed solid was filtered off and washed with absolute Et_2O (30 mL). **Mito-CORM 2-B** (180 mg, 0.337 mmol, 99%) was obtained as a yellow solid.

M($C_{19}H_{18}F_3FeNO_8S$): 533.25 g mol^{-1}.

Melting Point: 67 – 70 °C (EtOAc).

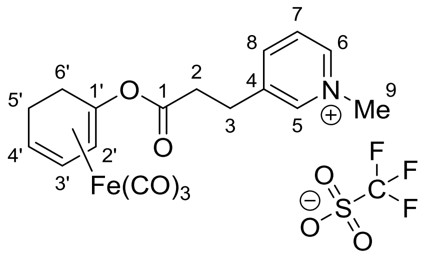

^{1}H NMR: (500 MHz, $CDCl_3$) δ = 8.83 (s, 1H, H-5), 8.73 (d, ^3J = 6.0 Hz, 1H, H-6), 8.33 (d, ^3J = 8.0 Hz, 1H, H-8), 7.90 (dd, ^3J = 8.0, 6.0 Hz, 1H, H-7), 5.35 (d, ^3J = 4.4 Hz, 1H, H-2‘), 5.11 (dd, ^3J = 6.5, 4.6 Hz, 1H, H-3‘), 4.44 (s, 3H, H-9), 3.16 (t, ^3J = 7.0 Hz, 2H, H-3), 3.13 – 3.09 (m, 1H, H-4‘), 2.83 – 2.79 (m, 2H, H-2), 2.12 (t, ^3J = 11.3 Hz, 1H,

H-6‘), 1.84 (td, ^3J = 11.4, 10.8, 3.5 Hz, 1H, H-5‘), 1.73 – 1.60 (m, 2H, H-5‘, H-6‘).

^{13}C NMR: (125 MHz, $CDCl_3$): δ = 211.4 ($Fe(CO)_3$), 170.1 (C-1), 145.6 (C-5), 145.5 (C-8), 143.4 (C-6), 142.4 (C-4), 127.8 (C-7), 121.0 (d, $^1J_{C\text{-}F}$ = 322 Hz, CF_3), 103.4 (C-1’), 81.2 (C-3’), 80.0 (C-2’), 61.0 (C-4’), 48.8 (C-9), 34.0 (C-2), 27.5 (C-3), 26.6 (C-6’), 24.1 (C-5’).

^{19}F NMR: (471 MHz, DMSO-d_6) δ = -77.8 (s).

LR-MS (ESI): $[M\text{-}CF_3SO_3]^+$ = 384.0 amu.

HR-MS (ESI): Calc.: $[M\text{-}CF_3SO_3]^+$ = 384.0529 amu, found. = 384.0526 amu.

FT-IR: (ATR) ῦ [cm^{-1}] = 3072 (w), 2924 (w), 2895 (w), 2857 (w), 2044 (s), 1957 (s), 17543(m), 1639 (w), 1511 (w), 1472 (w), 1454 (w), 1428 (w), 1329 (w), 1255 (s), 1224 (m), 1150 (s), 1107 (m), 1029 (s), 1005 (m), 901 (w), 865 (w), 816 (w), 755 (w), 682 (m), 637 (s), 610 (s), 560 (s), 516 (s), 493 (m).

1.2.9. (1,5-Cyclohexadiene-Fe(CO)$_3$)-1-yl-4-pyridine propionate (*rac*-8-A)

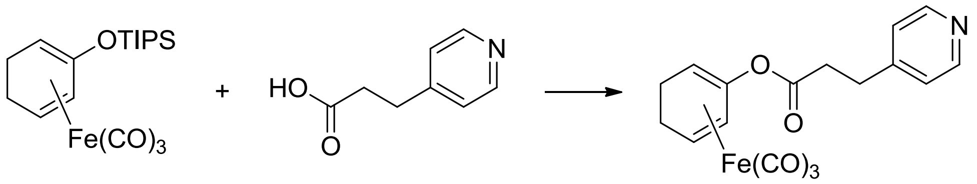

rac-**1-A** **7** *rac*-**8-A**

To a solution of TIPS-complex *rac*-**1-A** (202 mg, 0.51 mmol, 1.0 eq.) in CH_2Cl_2 (5.0 mL) at room temp was added a solution of TBAF (1 M in THF, 0.52 mL, 0.52 mmol, 1.0 eq.) and the mixture was stirred for 10 min at room temp. Then pyridine (0.14 mL, 1.68 mmol, 3.3 eq.) was added and the mixture was stirred for 10 min at room temp before EDC*HCl (215 mg, 1.12 mmol, 2.2 eq.), DMAP (19.0 mg, 0.15 mmol, 0.3 eq.) and acid **7** (85 mg, 0.56 mmol, 1.1 eq.) were added successively. The mixture was stirred for 4 h at room temp. Afterwards, the mixture was diluted with CH_2Cl_2 (20 mL) washed with saturated aqueous $NaHCO_3$ (5 mL), water (5 mL) and brine (5 mL). The organic phase was dried with $MgSO_4$ and the solvent was removed under reduced

pressure. The crude product was purified by column chromatography (ultra pure SiO_2, CyHex/EtOAc = 1:1) to yield *rac*-**8-A** (155 mg, 0.42 mmol, 82%) as a yellow oil.

M($C_{17}H_{15}FeNO_5$): 369.15 g mol^{-1}.

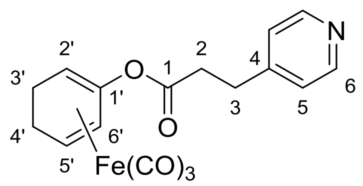

TLC: R_f (cHex/EtOAc = 1:1) = 0.25.

^{1}H NMR: (500 MHz, DMSO-d$_6$) δ = 8.51 – 8.40 (m, 1H, H-6), 7.35 – 7.14 (m, 1H, H-5), 5.75 (dd, ^3J = 6.7, ^4J = 2.0 Hz, 1H, H-6‘), 3.51 – 3.38 (m, 1H, H-2‘), 3.01 – 2.95 (m, 1H, H-5‘), 2.93 – 2.82 (m, 4H, H-2, H-3), 1.78 – 1.59 (m, 2H, H-3‘), 1.51 – 1.42 (m, 2H, H-4‘).

^{13}C NMR: (125 MHz, DMSO-d$_6$): δ = 211.0 (Fe(CO)$_3$), 171.7 (C-1), 149.5 (C-6), 148.9 (C-4), 128.1 (C-1’), 123.8 (C-5), 80.3 (C-6’), 59.4 (C-2’), 53.2 (C-5’), 33.3 (C-2), 29.1 (C-3), 24.2 (C-3’), 23.1 (C-4’).

LR-MS (ESI): $[M+H]^+$ = 370.0 amu.

HR-MS (ESI): Calc.: $[M+H]^+$ = 370.0372 amu, found. = 370.0374 amu.

FT-IR: (ATR) ṽ [cm^{-1}] = 3060 (w), 3022 (w), 2926 (m), 2894 (m), 2049 (s), 1963 (s), 1763 (m), 1590 (m), 1510 (m), 1488 (m), 1463 (m), 1445 (m), 1362 (w), 1327 (w), 1298 (w), 1269 (w), 1223 (s), 1165 (m), 1131 (w), 1089 (m), 1070 (m), 981 (w), 947 (s), 865 (m), 819 (s), 799 (s), 745 (s), 697 (w), 684 (w), 628 (m), 598 (w), 575 (m), 556 (m), 554 (w), 507 (w).

1.2.10. Mito-CORM 3-A

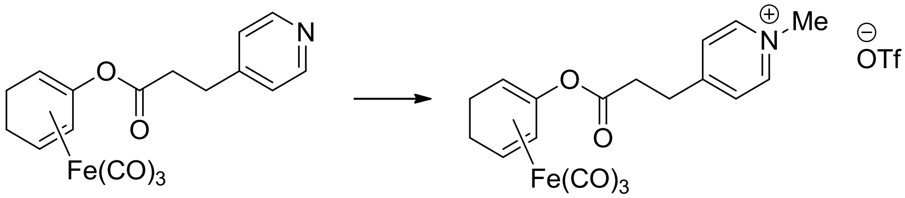

rac-**8-A** **Mito-CORM 3-A**

To a solution of complex *rac*-**8-A** (150 mg, 0.406 mmol, 1.0 eq.) in absolute Et_2O (4.0 mL) was added MeOTf (88.8 µL, 0.812 mmol, 2.0 eq.) dropwise. The mixture was stirred for 30 min at room temp and the formed solid was filtered off and washed with absolute Et_2O (30 mL). **Mito-CORM 3-A** (191 mg, 0.293 mmol, 60%) was obtained as a yellow solid.

M($C_{19}H_{18}F_3FeNO_8S$): 533.25 g mol^{-1}.

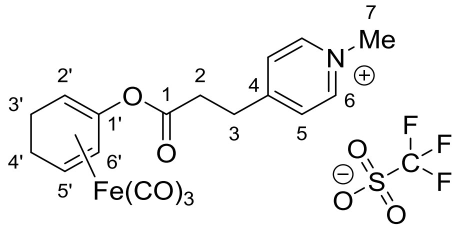

Melting Point 71 – 73 °C (Et_2O).

^{1}H NMR: (500 MHz, DMSO-d6) δ = 8.88 (d, ^3J = 6.3 Hz, 2H, H-6), 8.06 (d, ^3J = 6.3 Hz, 2H, H-5), 5.79 (dd, ^3J = 6.9, ^4J = 2.1 Hz, 1H, H-6‘), 4.29 (s, 3H, H-7), 3.49 – 3.47 (m, 1H, H-2‘), 3.20 (t, ^3J = 7.2 Hz, 2H, H-3), 3.07 (dd, ^3J = 7.1, 4.2 Hz, 1H, H-2), 3.04 – 3.01 (m, 1H, H-5‘), 1.75 – 1.61 (m, 2H, H-3‘), 1.57 – 1.40 (m, 2H, H-4‘).

^{13}C NMR: (125 MHz, DMSO-d6): δ = 211.0 ($Fe(CO)_3$), 171.4 (C-1), 159.8 (C-4), 144.8 (C-6), 128.0 (C-1‘), 127.3 (C-5), 120.7 (d, $^1J_{C\text{-}F}$ = 323 Hz, CF_3), 80.4 (H-6’), 59.4 (H-2’), 53.3 (H-5‘), 47.2 (C-7), 32.1 (H-2), 29.4 (H-3), 24.2 (H-3‘), 23.1 (H-4‘).

^{19}F NMR: (471 MHz, DMSO-*d*6) δ = -77.8 (s).

LR-MS (ESI): $[M\text{-}CF_3SO_3]^+$ = 384.0 amu.

HR-MS (ESI): Calc.: $[M\text{-}CF_3SO_3]^+$ = 384.0529 amu, found. = 384.0527 amu.

FT-IR: (ATR) ῦ [cm^{-1}] = 3138 (w), 3062 (w), 2934 (w), 2861 (w),2045 (s), 1957 (s), 1756 (m), 1647 (w), 1578 (w), 1523 (w), 1472 (w), 1458 (w), 1430 (w), 1373 (w), 1254 (s), 1224 (m), 1134 (s), 1076 (w), 997 (w), 927 (w), 864 (w), 837 (w), 772 (w), 637 (s), 609 (s), 569 (s), 515 (s).

1.2.11. (1,3-Cyclohexadiene-Fe(CO)3)-1-yl-4-pyridine propionate (*rac*-8-B)

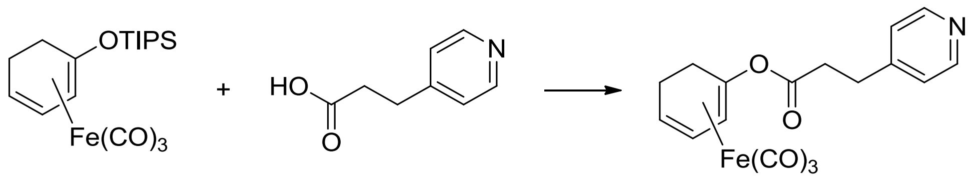

rac-**1-B** **7** *rac*-**8-B**

To a solution of TIPS complex *rac*-**1-B** (201 mg, 0.51 mmol, 1.0 eq.) in CH_2Cl_2 (5.0 mL) at room temp was added a solution of TBAF (1 M in THF, 0.52 mL, 0.52 mmol, 1.0 eq.) and the mixture was stirred for 10 min at room temp. Then pyridine (0.14 mL, 1.68 mmol, 3.3 eq.) was added and the mixture was stirred for 10 min at room temp before EDC*HCl (215 mg, 1.12 mmol, 2.2 eq.), DMAP (19.0 mg, 0.15 mmol, 0.3 eq.) and acid **7** (85 mg, 0.28 mmol, 1.1 eq.) were added successively. The mixture was stirred for 4 h at room temp. Afterwards, the mixture was diluted with CH_2Cl_2 (20 mL) washed with saturated aqueous $NaHCO_3$ (5 mL), water (5 mL) and brine (5 mL). The organic phase was dried with $MgSO_4$ and the solvent was removed under reduced pressure. The crude product was purified by column chromatography (ultra pure SiO_2, CyHex/EtOAc = 1:1) to yield *rac*-**8-B** (184 mg, 0.498 mmol, 97%) as a yellow oil.

M($C_{17}H_{15}FeNO_5$): 369.15 g mol^{-1}.

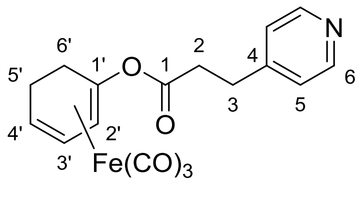

TLC: R_f (cHex/EtOAc = 1:1) = 0.25.

^{1}H NMR: (500 MHz, DMSO-d6) δ = 8.71 (s, 2H, H-6), 7.47 (s, 2H, H-5), 5.61 (d, ^3J = 4.1 Hz, 1H, H-2‘), 5.38 (t, ^3J = 5.1 Hz, 1H, H-3‘), 3.22 (t, ^3J

= 4.4 Hz, 1H, H-4‘), 2.92 (t, ^3J = 7.2 Hz, 2H, H-3), 2.82 – 2.65 (m, 2H, H-2), 2.03 – 1.95 (m, 1H, H-6‘), 1.79 – 1.70 (m, 1H, H-5‘), 1.69 – 1.58 (m, 2H, H-5‘, H-6‘).

^{13}C NMR: (125 MHz, DMSO-d$_6$): δ = 211.6 (Fe(CO)$_3$), 170.0 (C-1), 151.2 (C-5), 147.7 (C-6), 127.2 (C-4), 103.5 (C-1‘), 81.3 (C-3’), 80.1 (C-2’), 61.5 (C-4’), 33.4 (C-2), 29.3 (C-3), 26.1 (C-6’), 23.5 (C-5’).

LR-MS (ESI): [M+H]$^+$ = 370.0 amu.

HR-MS (ESI): Calc.: [M+H]$^+$ = 370.0372 amu, found. = 370.0368 amu.

Calc.: [M+Na]$^+$ = 392.0192 amu, found. = 392.0191 amu.

FT-IR: (ATR) ῦ [cm^{-1}] = 3069 (w), 3031 (w), 2940 (w), 2913 (w), 2857 (w), 2048 (s), 1969 (s), 1748 (m), 1602 (m), 1561 (w), 1415 (w), 1385 (w), 1328 (w), 1224 (w), 1182 (m), 1135 (s), 1110 (m), 1073 (w), 1004 (m), 901 (m), 812 (m), 618 (s), 562 (s), 489 (m).

1.2.12. Mito-CORM 3-B

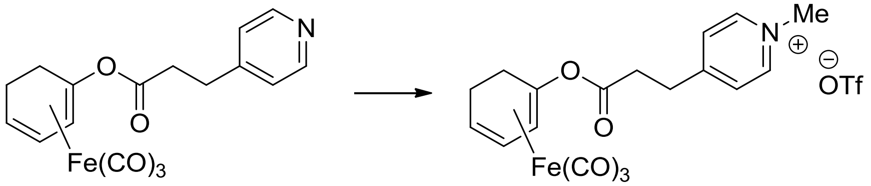

rac-**8-B** **Mito-CORM 3-B**

To a solution of complex *rac*-**8-B** (150 mg, 0.406 mmol, 1.0 eq.) in absolute Et_2O (4.0 mL) was added MeOTf (88.9 μL, 0.812 mmol, 2.0 eq.) dropwise. The mixture was stirred for 30 min at room temp the formed solid was filtered off and washed with absolute Et_2O (30 mL). **Mito-CORM 3-B** (191 mg, 0.358 mmol, 88%) was obtained as a yellow solid.

M($C_{19}H_{18}F_3FeNO_8S$): 533.25 g mol^{-1}.

Melting Point: 60 – 62 ° C (Et_2O).

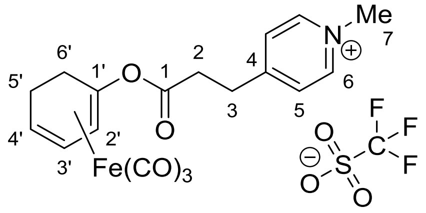

^{1}H NMR: (500 MHz, DMSO-d$_6$) δ = 8.90 – 8.79 (m, 2H, H-6), 8.10 – 7.95 (m, 2H, H-5), 5.63 (dd, ^3J = 4.6, ^4J = 1.3 Hz, 1H, H-2‘), 5.40 (dd, ^3J = 6.5, 4.6 Hz, 1H, H-3‘), 4.27 (s, 3H, H-7), 3.27 – 3.22 (m, 1H, H-4‘), 3.14 (t, ^3J = 7.2 Hz, 2H, H-3), 2.96 – 2.79 (m, 2H, H-2), 2.06 – 1.95 (m, 1H, H-6‘), 1.80 – 1.72 (m, 1H, H-5‘), 1.71 – 1.58 (m, 2H, H-5‘, H-6‘).

^{13}C NMR: (125 MHz, DMSO-d$_6$): δ = 212.2 ($Fe(CO)_3$), 170.3 (C-1), 160.5 (C-4), 145.2 (C-6), 127.7 (C-5), 121.2 (q, $^1J_{C\text{-}F}$ = 323 Hz, CF_3), 104.1 (C-1'), 81.9 (C-3'), 80.6 (C-2'), 62.2 (C-4'), 47.7 (C-7), 33.0 (C-2), 30.0 (C-3), 26.7 (C-6'), 24.0 (C-5').

^{19}F NMR: (471 MHz, DMSO-d_6) δ = -77.8 (s).

LR-MS (ESI): $[M\text{-}CF_3SO_3]^+$ = 384.0 amu.

HR-MS (ESI): Calc.: $[M\text{-}CF_3SO_3]^+$ = 384.0529 amu, found. = 384.0530 amu.

FT-IR: (ATR) ῦ [cm^{-1}] = 3134 (w), 3062 (w), 2947 (w), 2923 (w), 2901 (w), 2861 (w), 2046 (s), 1963 (s), 1744 (m), 1647 (m), 1578 (w), 1523 (w), 1455 (w), 1428 (w), 1382 (w), 1259 (s), 1224 (m), 1154 (s), 1108 (m), 1068 (w), 1030 (s), 905 (w), 865 (w), 837 (w), 756 (w), 638 (s), 614 (s), 562 (m), 518 (m), 458 (w).

1.2.13. 3,3,5-Trimethylcyclohexa-1,5-diene-1-yl acetate (10)

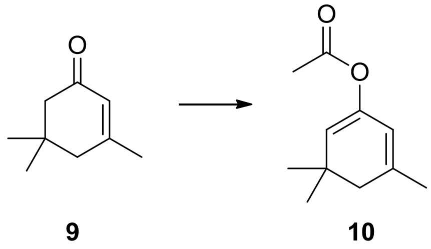

According to a procedure by *S. Romanski*[1], to a solution of diisopropylamine (1.65 mL, 11.7 mmol, 1.6 Äq.) in THF (10 mL) at –78 °C a solution of *n*BuLi (2.3 M in THF, 4.4 mL, 10.1 mmol, 1.4 Äq.) was added dropwise and the mixture was stirred at -78 °C for further 30 min. Subsequently, a solution of isophorone (**9**) (1.17 mL, 7.25 mmol, 1.0 Äq.) in THF (5 mL) was added dropwise at – 78 °C over a period of 1 h by a syringe pump. Then, aceticanhydride (0.81 mL, 8.6 mmol, 1.2 Äq.) was added dropwise, the cooling bath was removed and the mixture was stirred for 1 h at room temp. The reaction was stopped by addition of saturated aqueous NH_4Cl (25 mL) and extracted with MTBE (2 x 50 mL). The combined organic phases were washed with water (3 x 15 mL), dried over $MgSO_4$ and the solvent was removed under reduced pressure. The crude product was purified by column chromatography (SiO_2, CyHex/EtOAc = 30:1) to yield **10** (983 mg, 5.45 mmol, 76%) as a colorless oil.

M($C_{11}H_{16}O_2$): 180.25 g/mol

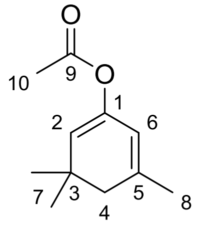

TLC: R_f(cHex/EtOAc 30:1) = 0.33.

^{1}H NMR: (500 MHz, $CDCl_3$): δ [ppm] = 5.45 (dt, ^4J = 3.2, 1.6 Hz, 1H, H-6), 5.01 – 5.00 (m, 1H, H-2), 2.12 (s, 3H, H-10), 2.03 – 2.02 (m, 2H, H-4), 1.78 (s, 3H, H-8), 1.03 (s, 6H, H-7).

^{13}C NMR: (125 MHz, $CDCl_3$): δ [ppm] = 169.6 (C-9), 145.2 (C-1), 138.0 (C-5), 119.1 (C-2), 116.8 (C-6), 43.7 (C-4), 32.5 (C-3), 28.5 (C-7), 23.5 (C-8), 21.2 (C-10).

**GC-MS:**	m/z (%) = 180 (12) $[M]^{+\cdot}$, 138 (5), 108 (5), 123 (100).
**HR-MS (DIP-MS 70 eV):**	Calc.: $[M]^{+}$ = 180.1145 amu, found.: 180.1144 amu.
**FT-IR:**	(ATR) $\tilde{\nu}$ [cm^{-1}] = 3441 (s), 2959 (m), 2927 (m), 2872 (w), 1756 (m), 1722 (m), 1688 (w), 1463 (w), 1409 (w), 1369 (m), 1268 (m), 1203 (s), 1115 (m), 1102 (s), 1089 (s), 1064 (m), 1046 (m), 1018 (m), 959 (m), 921 (m), 898 (m), 827 (m), 732 (m), 602 (w).

1.2.14. (3,3,5-Trimethylcyclohexa-1,5-diene-$Fe(CO)_3$)-1-yl acetate (rac-11-A') and (3,3,5-trimethylcyclohexa-1,3-diene-$Fe(CO)_3$)-1-yl acetate (rac-11-B')

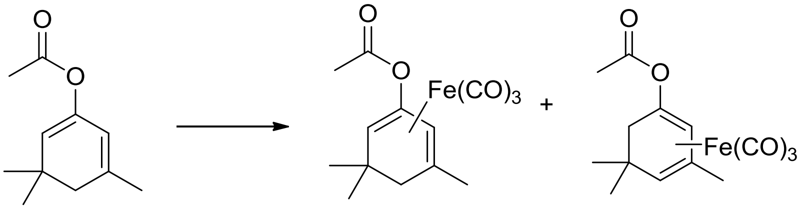

10 *rac*-**11-A'** 93:7 *rac*-**11-B'**

According to a procedure by *S. Romanski*[2], under an argon atmosphere, to a suspension of diirononacarbonyl (3.03 g, 8.3 mmol, 3.0 Äq.) in dry Et_2O (30 mL) the dienolacetate **10** (500 mg, 2.8 mmol, 1.0 Äq.) was added and the mixture was degassed with argon for 5 min. The mixture was heated at 40 °C for 48 hours before the solvent was removed under reduced pressure. The crude product was purified by column chromatography (ultra pure SiO_2, cHex/NEt_3 = 20:1) to yield a mixture of *rac*-**11-A'** and *rac*-**11-B'** (*rac*-**11-A'** / *rac*-**11-B'** 93:7) (669 mg, 2.08 mmol, 75%) as an orange/brown oil.

M($C_{14}H_{16}FeO_5$): 320.12 g/mol

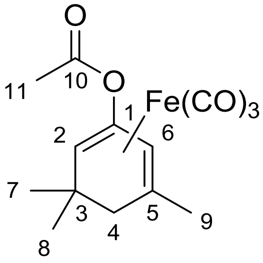

TLC: R_f(cHex/EtOAc 40:1) = 0.43.

^{1}H NMR: (500 MHz, $CDCl_3$): δ [ppm] = 5.60 (s, 1H, H-6), 2.98 (s, 1H, H-2), 2.18 (s, 3H, H-11), 1.63 – 1.41 (m, 5H, H-4/9), 1.10 (s, 3H, H-7/8), 1.08 (s, 3H, H-7/8).

^{13}C NMR: (125 MHz, $CDCl_3$): δ [ppm] = 211.5 ($Fe(CO)_3$), 169.9 (C-10), 124.0 (C-1), 84.4 (C-6), 70.0 (C-2), 67.7 (C-5), 48.7 (C-4), 36.7 (C-3), 34.6 (C-7/8), 30.9 (C-7/8), 25.3 (C-9), 21.2 (C-11).

LR-MS (ESI): $[M+Na]^+$ = 343.0 amu.

HR-MS (ESI): calc.: $[M+Na]^+$ = 343.0239 amu, found. = 343.0241 amu.

FT-IR: (ATR) $\tilde{\nu}$ [cm^{-1}] = 2958 (w), 2928 (w), 2907 (w), 2892 (w), 2866 (w), 2840 (w), 2039 (s), 1954 (s), 1768 (m), 1445 (w), 1419 (w), 1366 (m), 1302 (w), 1193 (s), 1153 (m), 1124 (m), 1107 (m), 1039 (w), 1017 (m), 981 (w), 960 (w), 932 (w), 899 (w), 883 (w), 846 (w), 734 (w), 638 (w), 602 (s), 577 (s), 567 (m), 538 (m), 507 (m), 492 (w), 456 (m).

M($C_{14}H_{16}FeO_5$): 320.12 g/mol

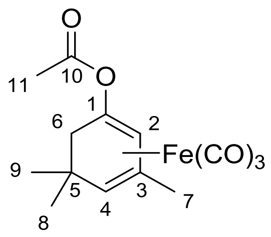

TLC: R_f(cHex/EtOAc 40:1) = 0.35.

^{1}H NMR: (500 MHz, $CDCl_3$): δ [ppm] = 5.34 (s, 1H, H-2), 2.73 (d, ^4J = 1.8 Hz, 1H, H-4), 2.14 – 2.08 (m, 4H, H-6/7), 2.03 (s, 3H, H-11), 1.70 (d, ^2J = 14.0 Hz, 1H, H-6), 1.11 (s, 3H, H-8/9), 0.97 (s, 3H, H-8/9).

**^{13}C NMR:**	(125 MHz, $CDCl_3$): δ [ppm] = 211.4 ($Fe(CO)_3$), 169.3 (C-10), 99.3 (C-1), 95.5 (C-3), 82.2 (C-2), 75.8 (C-4), 44.8 (C-6), 35.3 (C-5), 34.6 (C-8/9), 30.8 (C-8/9), 21.7 (C-7), 21.1 (C-11).
**LR-MS (ESI):**	$[M+Na]^+$ = 343.0 amu.
**HR-MS (ESI):**	$[M+Na]^+$ calc. = 343.0239 amu, found. = 343.0237 amu.
**FT-IR**:	(ATR) $\tilde{\nu}$ [cm^{-1}] = 2957 (w), 2927 (w), 2863 (w), 2840 (w), 2043 (s), 1958 (s), 1750 (m), 1451 (w), 1364 (m), 1301 (w), 1220 (s), 1198 (s), 1161 (w), 1120 (w), 1077 (m), 1040 (m), 1019 (w), 1010 (w), 959 (w), 894 (w), 849 (w), 644 (s), 608 (m), 585 (m), 565 (m), 538 (w), 522 (w), 507 (w), 462 (w).

1.2.15. 1-Tri*iso*propylsiloxy-3,3,5-trimethyl-1,5-cyclohexadiene-($Fe(CO)_3$) (rac-12-A')

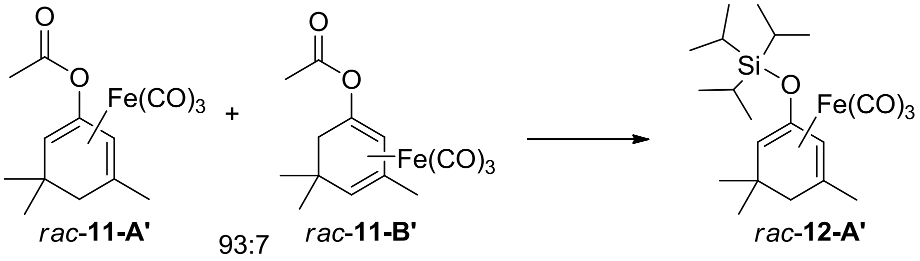

According to a previously published procedure[3], to a mixture of dienolacetate-complexes (93% *rac*-**11-A'**) (500 mg, 1.56 mmol, 1.0 eq.) and K_2CO_3 (216 mg, 1.56 mmol, 1.0 eq.) in DMF (10 mL) was added dropwise hydrazine (1 M in THF, 1.56 mL, 1.56 mmol, 1.0 eq.) and the mixture was stirred for 1 h at room temp. Afterwards, TIPSOTf (1.68 mL, 6.24 mmol, 4.0 eq.) was added and stirring was continued for additional 18 h. After addition of saturated aqueous NH_4Cl (25 mL) and extraction with EtOAc (3 x 50 mL) the combined organic layers were washed with saturated aqueous NH_4Cl (75 mL), brine (75 mL) and dried with $MgSO_4$. After removal of the solvent under reduced pressure, the crude product was purified by column chromatography (ultra pure SiO_2, cHex) to yield *rac*-**12-A'** (462 mg, 1.06 mmol, 68%) as a single regio isomer as a yellow oil.

M($C_{21}H_{34}FeO_4Si$): 434.43 g/mol

TLC: R_f(cHex) = 0.81.

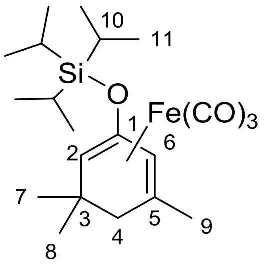

^{1}H NMR: (500 MHz, $CDCl_3$): δ [ppm] = 5.22 (s, 1H, H-6), 3.00 (s, 1H, H-2), 1.57 – 1.52 (m, 1H, H-4), 1.53 (s, 3H, H-9), 1.38 (d, ^2J = 15.2 Hz, 1H, H-4), 1.30 – 1.21 (m, 3H, H-10), 1.14 (d, ^3J = 7.1 Hz, 18H, H-11), 1.10 (s, 3H, H-7/8), 1.05 (s, 3H, H-7/8).

^{13}C NMR: (125 MHz, $CDCl_3$): δ [ppm] = 212.4 ($Fe(CO)_3$), 132.1 (C-1), 80.6 (C-6), 70.5 (C-2), 64.2 (C-5), 48.9 (C-4), 36.4 (C-3), 35.4 (C-7/8), 30.9 (C-7/8), 25.6 (C-9), 18.0 (C-11), 12.7 (C-10).

HR-MS (DIP-MS, 70 eV): m/z calc. for $[M-CO]^+$ = 406.1621 required; found: 406.1615.

FT-IR: (ATR) $\tilde{\nu}$ [cm^{-1}] = 2947 (w), 2893 (w), 2868 (w), 2033 (s), 1950 (s), 1480 (m), 1446 (m), 1404 (w), 1382 (w), 1362 (w), 1302 (w), 1260 (m), 1236 (w), 1162 (m), 1130 (m), 1116 (w), 1071 (w), 1040 (w), 1016 (w), 1003 (m), 992 (m), 971 (w), 919 (w), 901 (w), 881 (m), 869 (w), 826 (w), 761 (m), 676 (m), 651 (w), 605 (s), 579 (m), 554 (w), 532 (w), 511 (m).

1.2.16. (3,3,5-Trimethylcyclohexa-1,5-diene-$Fe(CO)_3$)-1-yl-3-pyridine propionate (rac-13-A’)

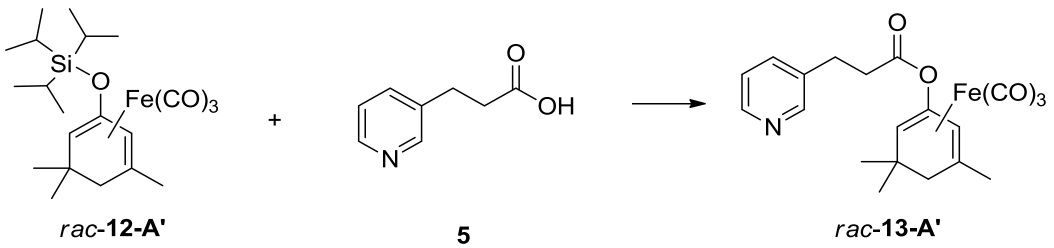

rac-**12-A'** **5** *rac*-**13-A'**

To a solution of TIPS-complex *rac*-**12-A’** (125 mg, 0.287 mmol, 1.0 eq.) in CH_2Cl_2 (3.0 mL) at room temp was added a solution of TBAF (1 M in THF, 0.29 mL, 0.29 mmol, 1.0 eq.) and the mixture was stirred for 10 min at room temp. Then pyridine (0.08 mL, 0.95 mmol, 3.3 eq.) was added and the mixture was stirred for 20 min at room temp before EDC*HCl (121 mg, 0.63 mmol, 2.2 eq.), DMAP (11 mg, 0.09 mmol, 0.3 eq.) and acid **5** (48 mg, 0.51 mmol, 1.1 eq.) were added successively. The mixture was stirred for 2 h at room temp. Afterwards the solvent was removed under reduced pressure. The crude product was purified by column chromatography (ultra pure SiO_2, cHex/EtOAc = 1:1) to yield *rac*-**13-A’** (72 mg, 0.175 mmol, 60%) as a yellow oil.

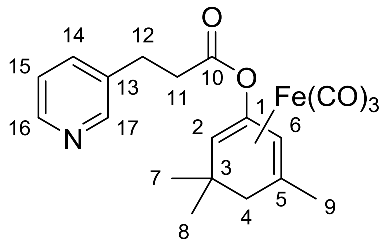

M($C_{20}H_{21}FeNO_5$): 411.24 g/mol

TLC: R_f(cHex/EtOAc 1:1) = 0.52.

^{1}H NMR: (500 MHz, $CDCl_3$): δ [ppm] = 8.53 (s, 1H, H-17), 8.51 (s, 1H, H-16), 7.56 (d, ^3J = 7.8 Hz, 1H, H-14), 7.25 – 7.22 (m, 1H, H-15), 5.52 (s, 1H, H-6), 3.01 (t, ^3J = 7.5 Hz, 2H, H-12), 2.89 (d, ^4J = 1.9 Hz, 1H, H-2), 2.78 (t, ^3J = 7.4 Hz, 2H, H-11), 1.62 – 1.52 (m, 2H, H-4), 1.54 (s, 3H, H-9), 1.48 – 1.39 (m, 1H, H-4‘), 1.07 (s, 3H, H-7/8), 1.06 (s, 3H, H-7/8).

^{13}C NMR: (125 MHz, $CDCl_3$): δ [ppm] = 211.4 ($Fe(CO)_3$), 171.3 (C-10), 149.9 (C-17), 148.1 (C-16), 135.9 (C-14), 135.3 (C-13), 123.9 (C-1), 123.6 (C-15), 84.4 (C-6), 70.0 (C-2), 67.86 (C-5), 48.6 (C-4), 36.6 (C-3), 35.5 (C-11), 34.6 (C-7/8), 30.9 (C-7/8), 27.9 (C-12), 25.2 (C-9).

LR-MS (ESI): $[M+H]^+$ = 412.0 amu.

HR-MS (ESI): $[M+H]^+$ calc. = 412.0842 amu, found. = 412.0843 amu.

FT-IR: (ATR) $\tilde{\nu}$ [cm^{-1}] = 3032 (w), 2957 (w), 2927 (w), 2864 (w), 2842 (w), 2038 (s), 1952 (s), 1759 (m), 1593 (w), 1576 (w), 1479 (w), 1445 (w),

1423 (w), 1379 (w), 1366 (w), 1299 (w), 1216 (m), 1174 (w), 1153 (w), 1127 (m), 1038 (w), 1028 (w), 999 (w), 981 (w), 960 (w), 931 (w), 897 (w), 869 (w), 847 (w), 801 (w), 713 (m), 611 (s), 575 (s), 536 (w), 522 (m), 507 (w).

1.2.17. Mito-CORM 2-A'

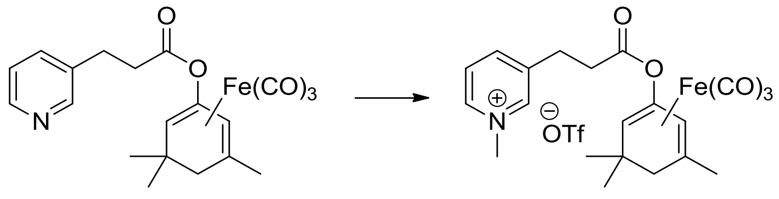

rac-**13-A'** **Mito-CORM 2-A'**

To a solution of complex *rac*-**13-A'** (70 mg, 0.170 mmol, 1.0 eq.) in absolute Et_2O (2.0 mL) was added MeOTf (37.2 µL, 0.340 mmol, 2.0 eq.) dropwise. The mixture was stirred for 30 min at room temp and the formed viscous yellow oil was washed with absolute Et_2O. During that process the oil solidified to give **Mito-CORM 2-A'** (50 mg, 0.087 mmol, 51%) as a yellow solid.

M($C_{22}H_{24}F_3FeNO_8S$): 575.33 g/mol

Melting Point: 90 – 92 °C (Et_2O).

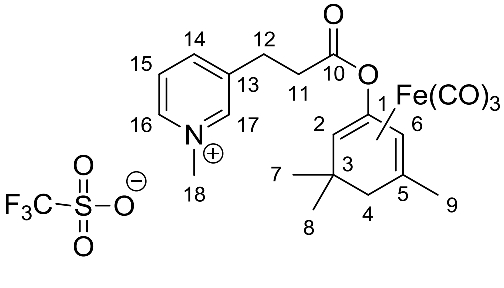

^{1}H NMR: (500 MHz, $CDCl_3$): δ [ppm] = 8.89 (s, 1H, H-17), 8.77 (d, ^3J = 6.0 Hz, 1H, H-16), 8.37 (d, ^3J = 8.0 Hz, 1H, H-14), 7.95 (dd, ^3J = 8.1, 6.0 Hz, 1H, H-15), 5.61 (s, 1H, H-6), 4.48 (s, 3H, H-18), 3.22 (t, ^3J = 7.0 Hz, 2H, H-12), 2.98 – 2.93 (m, 3H, H-2/11), 1.59 (d, ^2J = 15.6 Hz, 1H, H-4), 1.57 (s, 3H, H-9), 1.47 (d, ^2J = 15.4 Hz, 1H, H-4‘), 1.08 (s, 6H, H-7/8).

^{13}C NMR: (125 MHz, $CDCl_3$): δ [ppm] = 211.4 ($Fe(CO)_3$), 170.9 (C-10), 145.6 (C-17), 145.3 (C-14), 143.4 (C-16), 142.0 (C-13), 127.8 (C-15), 123.9

(C-19), 121.0 (d, $^1J_{C\text{-}F}$ = 319.3 Hz, CF_3), 84.3 (C-6), 69.7 (C-2), 68.3 (C-5), 48.8 (C-18), 48.6 (C-4), 36.6 (C-3), 34.6 (C-8), 33.9 (C-11), 30.8 (C-7), 27.3 (C-12), 25.2 (C-9).

^{19}F NMR: (471 MHz, DMSO-d_6) δ = -77.8 (s).

LR-MS (ESI): $[M\text{-}CF_3SO_3]^+$ = 426.1 amu.

HR-MS (ESI): $[M\text{- }CF_3SO_3]^+$ calc. = 426.0998 amu, found. = 426.1002 amu.

FT-IR: (ATR) $\tilde{\nu}$ [cm^{-1}] = 3060 (w), 2965 (w), 2924 (w), 2862 (w), 2044 (s), 1987 (s), 1969 (s), 1744 (m), 1641 (w), 1512 (w), 1479 (w), 1451 (w), 1423 (w), 1378 (w), 1367 (w),1257 (s), 1224 (s), 1212 (m), 1154 (s), 1108 (w), 1028 (s), 998 (w), 983 (w), 967 (w), 933 (w), 900 (w), 889 (w), 854 (w), 821 (w), 783 (w), 738 (w), 687 (m), 660 (w), 639 (s), 613 (s), 603 (s), 574 (s), 517 (m).

1.2.18. (3,3,5-Trimethylcyclohexa-1,5-diene-Fe(CO)3)-1-yl-4-pyridine propionate (*rac*-14-A')

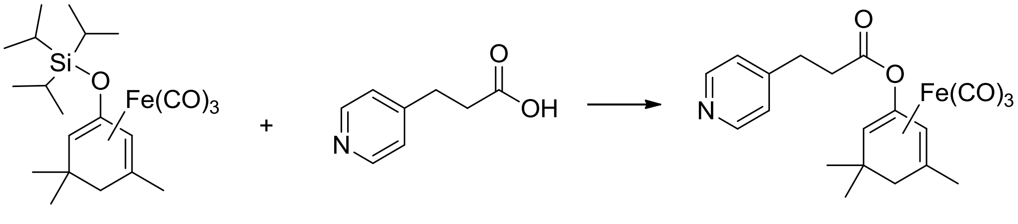

rac-**12-A'** **7** *rac*-**14-A'**

To a solution of TIPS-complex *rac*-**12-A'** (200 mg, 0.46 mmol, 1.0 eq.) in CH_2Cl_2 (5.0 mL) at room temp was added a solution of TBAF (1 M in THF, 0.46 mL, 0.46 mmol, 1.0 eq.) and the mixture was stirred for 10 min at room temp. Then pyridine (0.12 mL, 1.52 mmol, 3.3 eq.) was added and the mixture was stirred for 20 min at room temp before EDC*HCl (194 mg, 1.01 mmol, 2.2 eq.), DMAP (17 mg, 0.14 mmol, 0.3 eq.) and acid **7** (76 mg, 0.51 mmol, 1.1 eq.) were added successively. The mixture was stirred for 45 min at room temp. Afterwards the solvent was removed under reduced pressure. The crude product was purified by column chromatography (ultra pure SiO_2, CyHex/EtOAc = 1:1) to yield *rac*-**14-A'** (162 mg, 0.39 mmol, 86%) as a yellow oil.

M($C_{20}H_{21}FeNO_5$): 411.24 g/mol

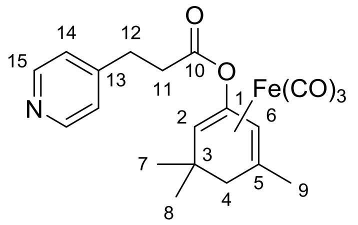

TLC: R_f(cHex/EtOAc 1:1) = 0.40.

^{1}H NMR: (500 MHz, DMSO-d$_6$): δ [ppm] = 8.49 (br s, 2H, H-15), 7.31 (s, 2H, H-14), 5.87 (s, 1H, H-6), 2.97 (s, 1H, H-2), 2.91 (m, 2H, H-12), 2.88 (m, 2H, H-11), 1.52 (s, 3H, H-9), 1.49 (s, 1H, H-4), 1.47 (s, 1H, H-4’), 1.02 (s, 3H, H-7/8), 1.01 (s, 3H, H-7/8).

^{13}C NMR: (125 MHz, DMSO-d$_6$): δ [ppm] = 212.4 (Fe(CO)$_3$), 171.4 (C-10), 149.5 (C-15), 148.9 (C-13), 123.8 (C-14), 123.7 (C-1), 84.7 (C-6), 70.4 (C-2), 69.1 (C-5), 48.1 (C-4), 36.3 (C-3), 34.2 (C-7/8), 33.5 (C-11), 30.5 (C-7/8), 29.2 (C-12), 24.8 (C-1).

LR-MS (ESI): $[M+H]^+$ = 412.0 amu.

HR-MS (ESI): $[M+H]^+$ calc. = 412.0842 amu, found. = 412.0842 amu.

FT-IR: (ATR) $\tilde{\nu}$ [cm^{-1}] = 3029 (w), 2957 (w), 2865 (w), 2842 (w), 2038 (s), 1952 (s), 1760 (m), 1602 (w), 1560 (w), 1445 (w), 1416 (w), 1379 (w), 1366 (w), 1300 (w), 1218 (m), 1176 (w), 1153 (m), 1128 (s), 1072 (w), 1039 (w), 1015 (w), 981 (w), 960 (w), 930 (w), 846 (w), 896 (w), 872 (w), 846 (w), 809 (w), 779 (w), 611 (s), 600 (s), 574 (s), 535 (m), 507 (m).

1.2.19. Mito-CORM 3-A’

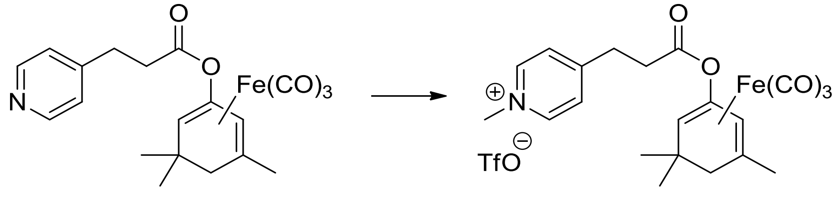

To a solution of complex *rac*-**14-A’** (100 mg, 0.234 mmol, 1.0 eq.) in absolute Et_2O (3.5 mL) was added MeOTf (53.2 µL, 0.486 mmol, 2.0 eq.) dropwise. The mixture was stirred for 45 min at room temp and the formed solid was filtered off and washed with absolute Et_2O (30 mL). Mito-CORM **Mito-CORM 3-A’** (120 mg, 0.209 mmol, 86%) was obtained as a yellow solid.

M($C_{22}H_{24}F_3FeNO_8S$): 575.33 g/mol

Melting Point: 128 – 130 °C (Et_2O).

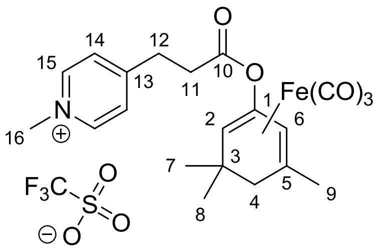

^{1}H NMR: (500 MHz, $CDCl_3$): δ [ppm] = 8.76 (d, ^3J = 4.8 Hz, 1H, H-15), 7.89 (d, ^3J = 6.2 Hz, 1H, H-14), 5.59 (s, 1H, H-6), 4.44 (d, ^3J = 2.9 Hz, 3H, H-16), 3.25 (t, ^3J = 7.1 Hz, 2H, H-12), 2.97 – 2.91 (m, 3H, H-2/11), 1.58 (d, ^2J = 15.5 Hz, 1H, H-4), 1.55 (s, 3H, H-9), 1.45 (d, ^2J = 15.4 Hz, 1H, H-4), 1.07 (s, 6H, H-7/8).

^{13}C NMR: (125 MHz, $CDCl_3$): δ [ppm] = 211.3 ($Fe(CO)_3$), 170.4 (C-10), 160.6 (C-13), 145.0 (C-15), 128.2 (C-14), 123.9 (C-1), 84.3 (C-6), 69.7 (C-2), 68.3 (C-5), 48.6 (C-4), 48.2 (C-16), 36.7 (C-3), 34.6 (C-7/8), 33.1 (C-11), 30.8 (C-7/8), 30.2 (C-12), 25.2 (C-9).

^{19}F NMR: (471 MHz, DMSO-d_6) δ = -77.8 (s).

LR-MS (ESI): $[M\ CF_3SO_3]^+$ = 426.0 amu.

HR-MS (ESI): $[M- CF_3SO_3]^+$ calc. = 426.0998 amu, found. = 426.0998 amu.

FT-IR: (ATR) $\tilde{\nu}$ [cm^{-1}] = 3063 (w), 2961 (w), 2046 (s), 1972 (s), 1758 (w), 1648 (w), 1576 (w), 1522 (w), 1478 (w), 1447 (w), 1367 (w), 1258 (s),1227 (m), 1144 (m), 1033 (m), 980 (w), 865 (w),837 (w), 758 (w), 645 (s), 613 (s), 575 (m), 518 (m).

1.2.20. (*E*)-Methyl 3-(pyridine-4-yl)acrylate (16)

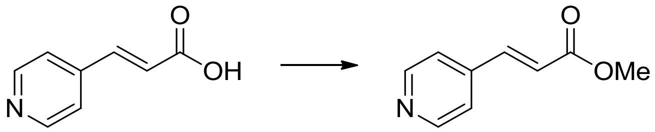

15 **16**

To a suspension of acid **15** (1.63 g, 10.9 mmol, 1.0 eq.) in MeOH (25 mL) was added dropwise conc. sulfuric acid (1.0 mL, 18.7 mmol, 1.7 eq.). The mixture was heated at 65 °C for 3 h. The solvent was removed under reduced pressure, the residue neutralized with saturated aqueous $NaHCO_3$ and the aqueous phase was extracted with MTBE (3 x 75 mL). The combinded organic phases were washed with water (75 mL) and dried with $MgSO_4$. The solvent was removed under reduced pressure and the product **16** (1.77 g, 10.8 mmol, >99%) was obtained as a colorless solid.

M($C_9H_9NO_2$): 163.18 g/mol

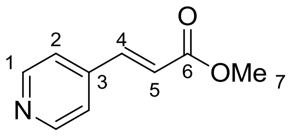

TLC: R_f(cHex/EtOAc 1:1) = 0.22.

Melting Point: 72 – 74 °C (MTBE).

^{1}H NMR: (300 MHz, $CDCl_3$): δ [ppm] = 8.70 – 8.62 (m, 2H, H-1), 7.61 (d, ^3J = 16.1 Hz, 1H, H-4), 7.41 – 7.33 (m, 2H, H-2), 6.60 (d, ^3J = 16.1 Hz, 1H, H-5), 3.83 (s, 3H, H-7).

^{13}C NMR: (75 MHz, $CDCl_3$): δ [ppm] = 166.4 (C-6), 150.6 (C-1), 141.9 (C-4), 141.5 (C-3), 122.4 (C-5), 121.8 (C-2), 52.0 (C-7).

GC-MS: m/z (%) = 163 (67), 132 (100), 104 (20), 78 (15), 51 (10), 163 (16).

FT-IR: (ATR) $\tilde{\nu}$ [cm^{-1}] = 3401 (w), 3346 (w), 3150 (w), 3053 (w), 3031 (m), 2949 (w), 2907 (w), 2842 (w), 2656 (w), 2140 (w), 2050 (w), 1995 (w), 1947 (w), 1923 (w), 1903 (w), 1862 (w), 1713 (s), 1668 (w), 1639 (m), 1597 (m), 1550 (m), 1503 (w), 1476 (w), 1459 (m), 1435 (m), 1421 (m), 1346 (w), 1323 (m), 1302 (m), 1238 (w), 1199 (m), 1172 (s), 1076 (m), 1035 (w), 1010 (s), 990 (s), 939 (m), 909 (m), 896 (m), 845 (m), 816 (s), 752 (m), 728 (s), 667 (w), 647 (w), 569 (s), 507 (s), 514 (s).

The spectroscopic data are in accordance with the literature.[4]

1.2.21. (*rac*)-Methyl 2-(pyridine-4-yl)cyclopropanecarboxylate (*rac*-17)

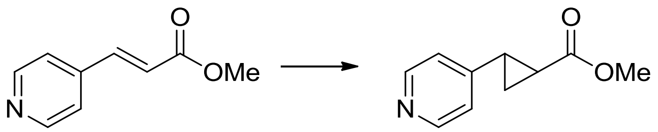

According to a modified procedure, [5] trimethylsulfoxonium iodide (1.24g, 5.62 mmol, 1.6 eq.) and sodium hydride (220 mg, 6.20 mmol, 1.8 eq.) were added to a flame dried flask and dry DMSO (20 mL) was added over 10 min at room temp and stirred for further 10 min. Then, a solution of ester **16** (560 mg, 3.45 mmol, 1.0 eq.) in dry DMSO (30 mL) was added dropwise and the orange mixture was stirred at room temp for 19 h. The mixture was diluted with water (100 mL) and extracted with MTBE (3 x 100 mL). The combined organic phases were washed with brine (100 mL) dired with $MgSO_4$ and the solvent was removed under reduced pressure. The crude product was purified by column chromatography (SiO_2, cHex/EtOAc = 1:1) to yield *rac*-**17** (180 mg, 1.01 mmol, 29%) as a colourless oil.

M($C_{10}H_{11}NO_2$): 177.20 g/mol

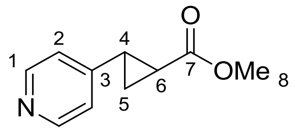

TLC: R_f(cHex/EtOAc 1:1) = 0.25.

**^{1}H NMR:**	(500 MHz, $CDCl_3$): δ [ppm] = 8.47 – 8.46 (m, 2H, H-1), 7.00 – 6.98 (m, 2H, H-2), 3.73 (s, 3H, H-8), 2.46 (ddd, ^3J = 9.1, 6.3, 4.1 Hz, 1H, H-6), 2.01 – 1.98 (ddd, ^3J = 8.5, 5.5, 4.1 Hz, H-4), 1.71 – 1.67 (ddd, ^3J = 9.1, 5.5, ^2J = 4.8 Hz, H-5α), 1.38 – 1.34 (ddd, ^3J = 8.6, 6.4, ^2J = 4.8 Hz, H-5β).
**^{13}C NMR:**	(125 MHz, $CDCl_3$): δ [ppm] = 173.0 (C-7), 149.8 (C-1), 149.4 (C-3), 121.2 (C-2), 52.1 (C-8), 25.1 (C-6), 24.5 (C-4), 17.5 (C-5).
**GC-MS:**	m/z (%) = 177 (47), 146 (50), 132 (12), 118 (100), 91 (31), 59 (11).
**HR-MS (EI-70 eV):**	$[M-H]^+$ calc. = 176.0706 amu, found. = 176.0698 amu.
**FT-IR:**	(ATR) $\tilde{\nu}$ [cm^{-1}] = 3441 (w), 3029 (w), 2953 (w), 2105 (m), 1938 (w), 1724 (s), 1601 (s), 1557 (w), 1496 (w), 1450 (m), 1437 (m), 1413 (w), 1401 (m), 1346 (m), 1320 (m), 1273 (m), 1243 (w), 1199 (s), 1172 (s), 1118 (w), 1087 (w), 1069 (w), 1044 (m), 991 (m), 936 (m), 907 (m), 845 (m), 831 (w), 809 (s), 787 (m), 742 (w), 683 (w), 667 (w), 652 (w), 560 (m), 544 (m).

1.2.22. (*rac*)-Cyclopropanecarboxylic acid, 2-(4-pyridinyl)-hydrochloride (*rac*-18)

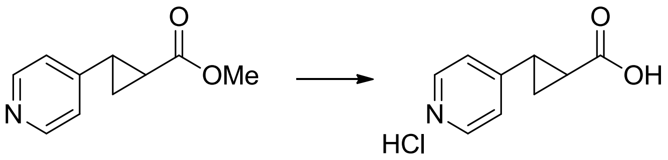

rac-**17** *rac*-**18**

To a solution of ester *rac*-**17** (353 mg, 1.99 mmol, 1.0 eq.) in a mixture of H_2O/MeOH (1:1, 6.0 mL) was added NaOH (239 mg, 5.97 mmol, 3.0 eq.). The mixture was stirred at room temp for 72 h and then acidified to pH = 2 by addition of hydrochloric acid (1 M). The solvent was evaporated and the product *rac*-**18** (2.04 g, quant.) was obtained as a white solid. The product contained NaCl but was used without purification for the next step.

M($C_9H_9NO_2$): 163.18 g/mol

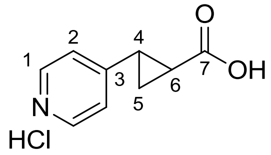

Melting Point: 228 – 235 °C (H_2O).

^{1}H NMR: (500 MHz, DMSO-d$_6$): δ [ppm] = 12.78 (s, 1H, CO_2H), 8.77 (d, ^3J = 6.8 Hz, 2H, H-1), 7.89 (d, ^3J = 6.8 Hz, 2H, H-2), 2.77 (ddd, ^3J = 9.1, 6.1, 4.1 Hz, 1H, H-6), 2.30 (ddd, ^3J = 8.8, 5.8, 4.1 Hz, 1H, H-4), 1.73 – 1.64 (m, 2H, H-5).

^{13}C NMR: (125 MHz, DMSO-d$_6$): δ [ppm] = 172.5 (C-7), 161.5 (C-3), 141.0 (C-1), 124.0 (C-2), 26.8 (C-6), 24.8 (C-4), 19.2 (C-5).

LR-MS (ESI): $[M+H]^+$ = 164.0 amu.

HR-MS (ESI): $[M+H]^+$ calc. = 164.0706 amu, found. = 164.0705 amu.

FT-IR: (ATR) $\tilde{\nu}$ [cm^{-1}] = 3067 (m), 2697 (br), 2051 (w), 1980 (w), 1716 (s), 1636 (s), 1601 (m), 1508 (m), 1450 (m), 1422 (m), 1369 (m), 1317 (w), 1291 (w), 1251 (w), 1221 (w), 1169 (s), 1088 (m), 1060 (m), 1038 (m), 1007 (w), 952 (w), 936 (m), 834 (s), 805 (s), 783 (m), 730 (m), 641 (s), 563 (w), 540 (s).

1.2.23. Complex *ambo-3-B*

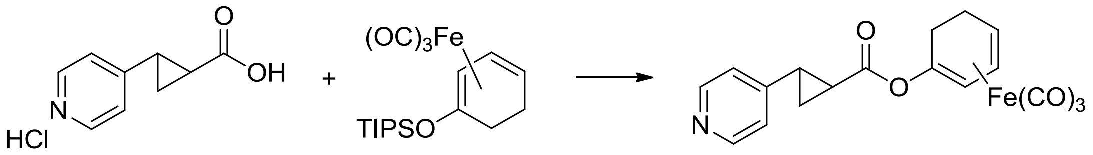

rac-**18** *rac*-**1-B** *ambo*-**3-B**

To a solution of TIPS-complex *rac*-**1-B** (100 mg, 0.25 mmol, 1.0 eq.) in CH_2Cl_2 (2.5 mL) at room temp was added a solution of TBAF (1 M in THF, 0.25 mL, 0.25 mmol, 1.0 eq.) and the mixture was stirred for 10 min at room temp. Then pyridine (0.09 mL, 1.06 mmol, 4.3 eq.) was added and the mixture was stirred for 15 min at room temp before EDC*HCl (104 mg, 0.54 mmol, 2.2 eq.), DMAP (9.0 mg, 0.07 mmol, 0.3 eq.) and acid *rac*-**18** (80 mg, 0.49 mmol, 2.0 eq.) were added

successively. The mixture was stirred for 21 h at room temp. The solvent was removed under reduced pressure and the crude product was purified by column chromatography (ultra pure SiO_2, cHex/EtOAc = 1:1) to yield *ambo*-**3-B** (72 mg, 0.19 mmol, 65%) as a yellow oil.

M($C_{18}H_{15}NO_5Fe$): 381.17 g/mol

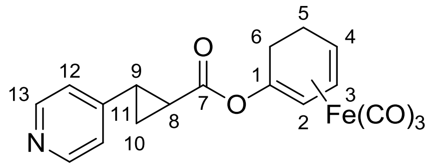

TLC: R_f(cHex/EtOAc 1:1) = 0.28.

^{1}H NMR: (500 MHz, $CDCl_3$, (mixture of diastereomers)): δ [ppm] = 8.64 – 8.29 (m, 2H, H-13), 7.07 – 6.89 (m, 2H, H-12), 5.51 – 5.27 (m, 1H, H-2), 5.22 – 5.01 (m, 1H, H-3), 3.20 – 3.01 (m, 1H, H-4), 2.56 – 2.39 (m, 1H, H-8), 2.26 – 2.13 (m, 1H, H-6), 2.02 – 1.82 (m, 2H, H-5, H-9), 1.86 – 1.54 (m, 3H, H-5, H-6, H-10), 1.49 – 1.28 (m, 1H, H-10).

^{13}C NMR: (125 MHz, $CDCl_3$, (mixture of diastereomers)): δ [ppm] = 211.3 ($Fe(CO)_3$), 170.9/170.8 (C-7), 150.0/150.0 (C-13), 149.3/149.2 (C-11), 121.4/121.3 (C-12), 103.5/103.4 (C-1), 81.0/81.0 (C-3), 80.1/80.1 (C-2), 60.7/60.6 (C-4), 26.8/26.8 (C-6), 25.7/25.3 (C-8), 25.0/24.9 (C-9), 24.2 (C-5), 17.8/17.1 (C-10).

LR-MS (ESI): $[M+H]^+$ = 382.0 amu.

HR-MS (ESI): $[M+H]^+$ calc. = 382.0372 amu, found. = 382.0370 amu.

FT-IR: (ATR) $\tilde{\nu}$ [cm^{-1}] = 3068 (w), 2986 (w), 2932 (w), 2878 (w), 1567 (w), 1461 (m), 1404 (w), 1379 (m), 1370 (m), 1257 (m), 1220 (s), 1169 (m), 1133 (w), 1100 (m), 1079 (s), 991 (m), 915 (w), 896 (w), 845 (m), 765 (s), 741 (m), 684 (w), 634 (w), 514 (w), 479 (w).

1.2.24. Mito-CORM 4-B

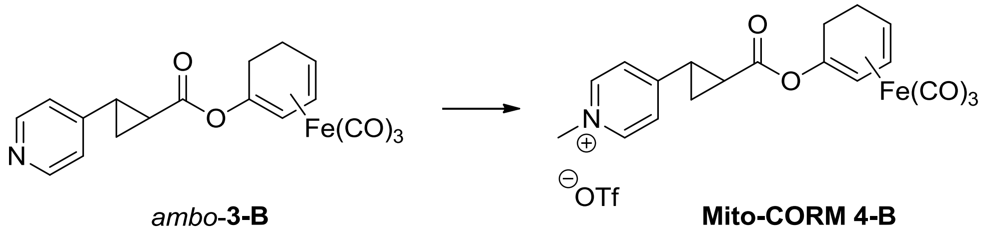

To a solution of complex *ambo*-**3-B** (72 mg, 0.19 mmol, 1.0 eq.) in absolute Et_2O (2.0 mL) was added MeOTf (41.0 µL, 0.38 mmol, 2.0 eq.). The mixture was stirred for 1.5 h at room temp and the solvent was decanted. The residue was washed with absolute Et_2O (30 mL) and dried under high vacuum to form Mito-CORM Mito-CORM 4-B (100 mg, 0.18 mmol, 97%) as a pale yellow solid.

M($C_{20}H_{18}NO_8F_3SFe$): 545.26 g/mol

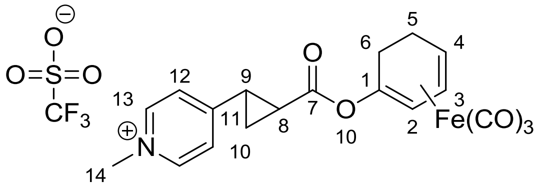

Melting Point: 72 – 80 °C.

^{1}H NMR: (500 MHz, $CDCl_3$, (mixture of diastereomers)): δ [ppm] = 8.94 – 8.44 (m, 2H, H-13), 7.68 – 7.43 (m, 2H, H-12), 5.53 – 5.28 (m, 1H, H-2), 5.21 – 5.04 (m, 1H, H-3), 4.38 (s, 3H, H-14), 3.19 – 3.04 (m, 1H, H-4), 2.83 – 2.52 (m, 1H, H-9), 2.38 – 2.08 (m, 2H, H-6, H-8), 2.04 – 1.78 (m, 2H, H-5, H-10), 1.81 – 1.51 (m, 3H, H-5, H-6, H-9).

^{13}C NMR: (125 MHz, $CDCl_3$, (mixture of diastereomers)): δ [ppm] = 211.3 ($Fe(CO)_3$), 169.5/169.4 (C-7), 161.3/161.3 (C-11), 145.1/145.1 (C-13), 125.2/125.2 (C-12), 103.5/103.5 (C-1), 81.3/81.2 (C-3), 79.9/79.9 (C-2), 61.0/60.9 (C-4), 48.5 (C-14), 27.9/27.8 (C-6), 26.6/26.6 (C-8), 26.0/25.5 (C-10), 24.1/24.1 (C-5), 20.0/19.3 (C-9).

^{19}F NMR: (471 MHz, DMSO-d_6) δ = -77.5 (s).

LR-MS (ESI): $[M-CF_3SO_3]^+$ = 396.0 amu.

HR-MS (ESI): $[M\text{-}CF_3SO_3]^+$ calc. = 396.0534 amu, found. = 396.0502 amu.

FT-IR: (ATR) $\tilde{\nu}$ [cm^{-1}] = 3492 (w), 3062 (w), 2859 (w), 2324 (w), 2252 (w), 2114 (w), 2046 (s), 1962 (s), 1737 (m), 1644 (m), 1574 (w), 1526 (w), 1473 (w), 1456 (w), 1429 (w), 1410 (w), 1383 (w), 1350 (w), 1328 (m), 1255 (s), 1224 (m), 1200 (m), 1158 (s), 1116 (m), 1067 (w), 1029 (s), 1007 (m), 906 (m), 863 (m), 823 (m), 757 (w), 702 (w), 731 (m), 702 (w), 666 (w), 637 (s), 613 (s), 563 (m), 539 (m), 518 (m).

1.2.25. Methyl 3-(pyridine-3-yl)propanoate (19)

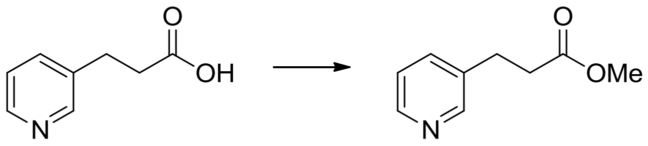

5 **19**

To a suspension of acid **5** (129 mg, 0.86 mmol, 1.0 eq.) in MeOH (4 mL) at 0 °C was added dropwise $SOCl_2$ (0.093 mL, 1.29 mmol, 1.5 eq.). The mixture was stirred at room temp for 5 h. The reaction was stopped by the addition of saturated aqueous $NaHCO_3$ (5 mL) and the aqueous phase was extracted with CH_2Cl_2 (3 x 5 mL). The combined organic phases were dried with $MgSO_4$ and the solvent was removed under reduced pressure to yield the product **19** (141 mg, 0.86 mmol, 100%) as a colorless oil.

M($C_9H_{11}NO_2$): 165.19 g/mol

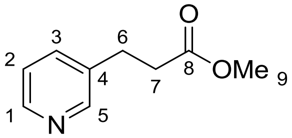

TLC: R_f(cHex/EtOAc 1:1) = 0.14.

^{1}H NMR: (400 MHz, $CDCl_3$): δ [ppm] = 8.53 – 8.34 (m, 2H, H-1, H-5), 7.49 (dtd, ^3J = 7.8, ^4J = 1.7, 0.8 Hz, 1H, H-3), 7.17 (ddd, ^3J = 7.8, 4.8, J = 0.9 Hz, 1H, H-2), 3.63 (s, 3H, H-8), 2.91 (t, ^3J = 7.6 Hz, 2H, H-6), 2.61 (t, ^3J = 7.6 Hz, 2H, H-7).

^{13}C NMR: (100 MHz, $CDCl_3$): δ [ppm] = 172.8 (C-8), 149.9 (C-5), 147.9 (C-1), 135.9 (C-3), 135.9 (C-4), 123.4 (C-2), 51.8 (C-9), 35.2 (C-7), 28.1 (C-6).

^{19}F NMR: (376 MHz, $CDCl_3$) δ = -77.8 (s).

GC-MS: m/z (%) = 165 (14), 150 (23), 135 (100), 120 (2), 105 (83), 78 (17), 51 (16).

FT-IR: (ATR) $\tilde{\nu}$ [cm^{-1}] = 3030 (w), 2998 (w), 2952 (w), 1732 (s), 1592 (w), 1576 (w), 1480 (w), 1436 (m), 1424 (m), 1366 (w), 1297 (w), 1255 (m), 1194 (m), 1174 (s), 1155 (m), 1105 (w), 1027 (m), 986 (w), 951 (w), 900 (w), 839 (w), 802 (m), 714 (s), 630 (m), 614 (w), 574 (w), 500 (w).

The spectroscopic data are in accordance with the literature.[6]

1.2.26. [Methyl 3-(*N*-methylpyridinium-3-yl)propanoate] triflate (LHP545)

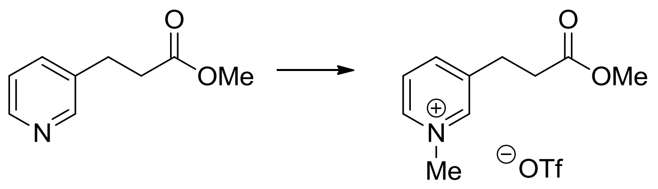

19 **LHP545**

To a solution of **19** (261 mg, 1.58 mmol, 1.0 eq.) in absolute Et_2O (16 mL) was added MeOTf (0.18 mL, 3.16 mmol, 2.0 eq.). The mixture was stirred for 0.5 h at room temp and the solvent was decanted. The residue was washed with absolute Et_2O (30 mL) and the crude product was purified by column chromatography (ultra pure SiO_2, CH_2Cl_2/MeOH = 10:1) to yield **LHP545** (470 mg, 1.42 mmol, 90%) as a colorless oil.

M($C_{11}H_{14}F_3NO_5S$): 329.29 g/mol

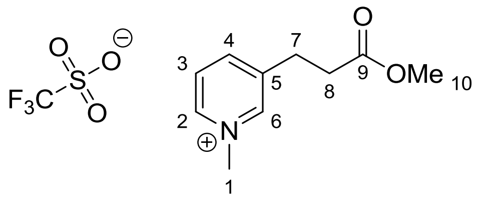

**TLC:**	$R_f(CH_2Cl_2/MeOH\ 10{:}1) = 0.12$.
**^{1}H NMR:**	(500 MHz, $CDCl_3$): δ [ppm] = 8.96 (s, 1H, H-6), 8.83 (dd, ^3J = 5.9, ^4J = 1.5 Hz, 1H, H-2), 8.48 (dt, ^3J = 8.3, ^4J = 1.5 Hz, 1H, H-4), 8.05 (dd, ^3J = 8.1, 6.0 Hz, 1H, H-3), 4.31 (s, 3H, H-1), 3.60 (s, 3H, H-10), 3.04 (t, ^3J = 7.4 Hz, 2H, H-7), 2.80 (t, ^3J = 7.4 Hz, 2H, H-8).
**^{13}C NMR:**	(125 MHz, $CDCl_3$): δ [ppm] = 172.1 (C-9), 145.1 (C-6), 144.9 (C-2), 143.3 (C-4), 141.0 (C-5), 127.1 (C-3), 120.7 (d, 322 Hz, CF_3), 51.5 (C-10), 47.8 (C-1), 33.0 (C-8), 26.9 (C-7).
**HR-MS (ESI):**	$[M\text{-}OTf]^+$ calc. = 180.1019 amu, found. = 180.1019 amu.
**FT-IR:**	(ATR) $\tilde{\nu}$ [cm^{-1}] = 3525 (w), 3074 (w), 2957 (w), 1732 (m), 1640 (w), 1511 (w), 1441 (w), 1373 (w), 1258 (s), 1225 (m), 1155 (s), 1029 (s), 815 (w), 757 (w), 681 (w), 637 (s), 573 (w), 517 (m), 422 (w).

1.2.27. Methyl 3-(pyridine-4-yl)propanoate (20)

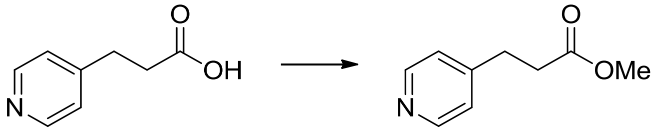

7 **20**

To a suspension of acid **7** (532 mg, 3.52 mmol, 1.0 eq.) in MeOH (20 mL) at 0 °C was added dropwise $SOCl_2$ (0.38 mL, 5.28 mmol, 1.5 eq.). The mixture was stirred at room temp for 3 h. The reaction was stopped by the addition of saturated aqueous $NaHCO_3$ (15 mL) and the aqueous phase was extracted with CH_2Cl_2 (3 x 15 mL). The combined organic phases were dried with $MgSO_4$ and the solvent was removed under reduced pressure to yield the product **20** (574 mg, 3.31 mmol, 94%) as a colorless oil.

M($C_9H_{11}NO_2$): 165.19 g/mol

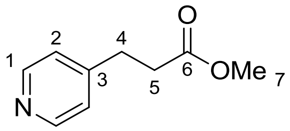

TLC: R_f(cHex/EtOAc 1:1) = 0.14.

**^{1}H NMR:**	(500 MHz, $CDCl_3$): δ [ppm] = 8.44 – 8.17 (m, 2H, H-1), 7.12 – 6.79 (m, 2H, H-2), 3.51 (s, 3H, H-7), 2.81 – 2.74 (m, 2H, H-4), 2.56 – 2.40 (m, 2H, H-5).
**^{13}C NMR:**	(125 MHz, $CDCl_3$): δ [ppm] = 172.4 (C-6), 149.7 (C-1), 149.2 (C-3), 123.5 (C-2), 51.5 (C-7), 33.9 (C-5), 29.8 (C-4).
**GC-MS:**	m/z (%) = 165 (58), 134 (33), 120 (9), 105 (100), 78 (17), 51 (16).
**FT-IR:**	(ATR) $\tilde{\nu}$ [cm^{-1}] = 3072 (w), 3028 (w), 2994 (w), 2953 (w), 2844 (w), 1733 (s), 1559 (w), 1497 (w), 1437 (m), 1416 (m), 1367 (w), 1342 (w), 1295 (w), 1255 (m), 1198 (s), 1170 (s), 1090 (w), 1070 (w), 992 (m), 957 (w), 904 (w), 836 (m), 811 (m), 780 (w), 733 (w), 561 (m), 523 (w), 496 (m), 433 (w).

The spectroscopic data are in accordance with the literature.[7]

1.2.28. [Methyl 3-(N-methylpyridinium-4-yl)propanoate] triflate (LHP551)

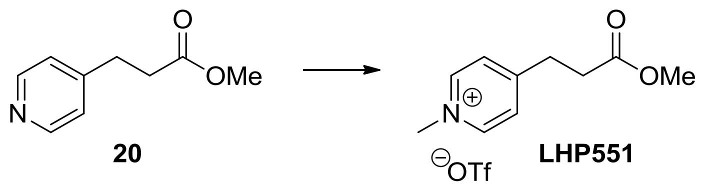

To a solution of **20** (300 mg, 1.82 mmol, 1.0 eq.) in absolute Et_2O (18 mL) was added MeOTf (0.41 mL, 3.63 mmol, 2.0 eq.). The mixture was stirred for 0.5 h at room temp and the solvent was decanted. The residue was washed with absolute Et_2O (30 mL) and the crude product was purified by column chromatography (ultra pure SiO_2, CH_2Cl_2/MeOH = 10:1) to yield **LHP551** (558 mg, 1.70 mmol, 93%) as a colorless oil.

M($C_{11}H_{14}F_3NO_5S$): 329.29 g/mol

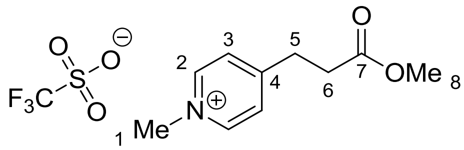

TLC: R_f(CH_2Cl_2/MeOH 10:1) = 0.12.

^{1}H NMR: (400 MHz, $CDCl_3$): δ [ppm] = 8.74 (d, ^{3}J = 6.6 Hz, 2H, H-2), 7.85 (d, ^{3}J = 6.6 Hz, 2H, H-3), 4.42 (s, 3H, H-1), 3.67 (s, 3H, H-8), 3.20 (t, ^{3}J = 7.0 Hz, 2H, H-5), 2.80 (t, ^{3}J = 7.0 Hz, 2H, H-6).

^{13}C NMR: (100 MHz, $CDCl_3$): δ [ppm] = 171.9 (C-7), 161.3 (C-4), 145.0 (C-2), 128.3 (C-3), 120.7 (d, ^{1}J = 320 Hz, CF_3), 52.3 (C-8), 48.3 (C-1), 32.9 (C-6), 30.5 (C-5).

^{19}F NMR: (376 MHz, $CDCl_3$) δ = -77.5 (s).

HR-MS (ESI): $[M\text{-}OTf]^{+}$ calc. = 180.1019 amu, found. = 180.1020 amu.

FT-IR: (ATR) $\tilde{\nu}$ [cm^{-1}] = 3496 (w), 3128 (w), 3064 (w), 2960 (w), 2928 (w), 1728 (m), 1647 (m), 1578 (w), 1522 (w), 1476 (w), 1440 (w), 1420 (w), 1374 (w), 1248 (s), 1223 (s), 1154 (s), 1027 (s), 914 (w), 838 (m), 758 (w), 635 (s), 573 (m), 516 (s), 460 (m).

NMR Spectra

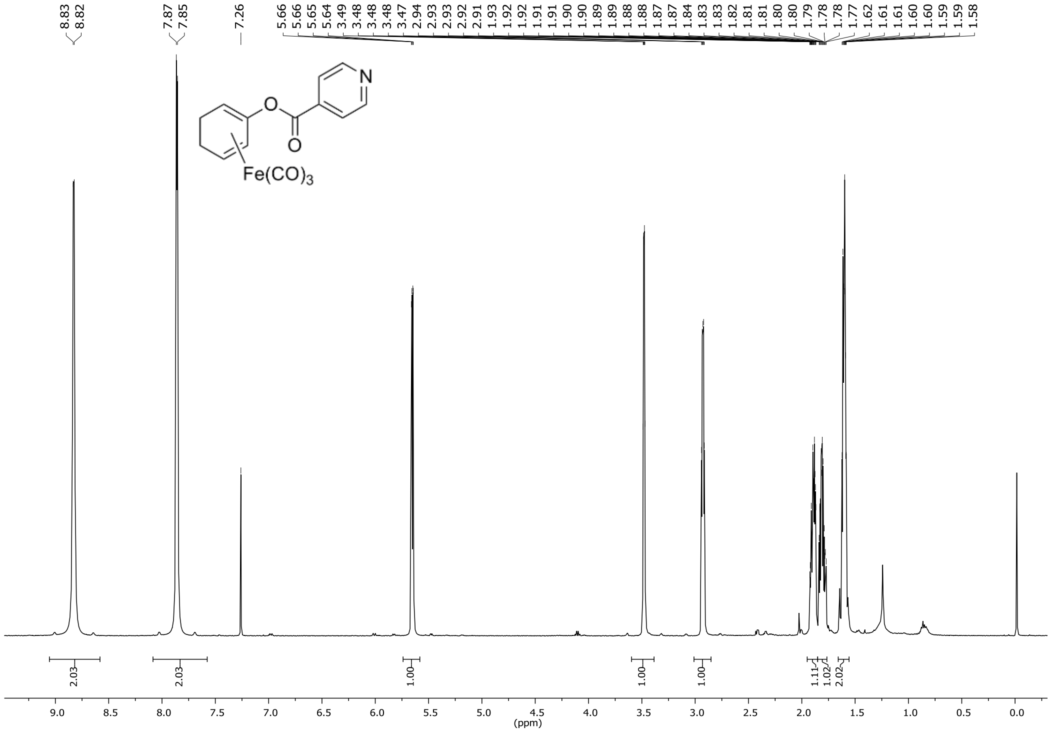

Figure S 1: ^{1}H NMR of (1,5-cyclohexadien-Fe(CO)$_3$)-1-yl isonicotinate (*rac*-**2-A**) (500 MHz, CDCl$_3$).

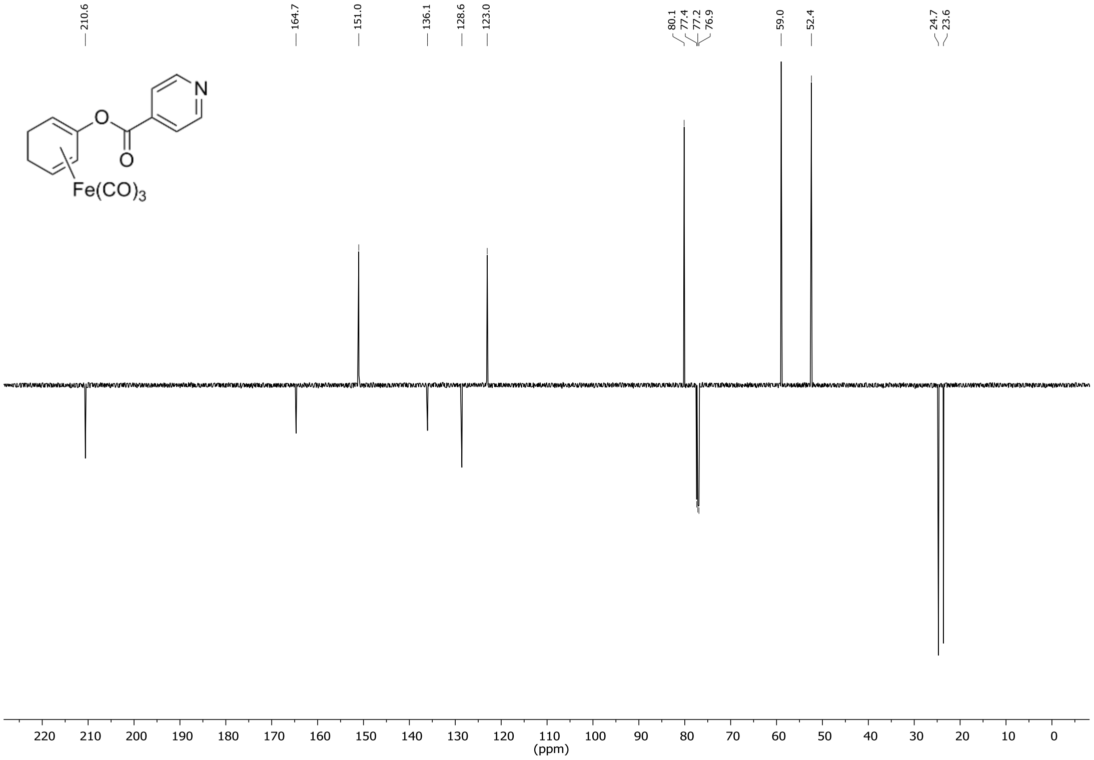

Figure S 2: ^{13}C NMR of (1,5-cyclohexadien-Fe(CO)$_3$)-1-yl isonicotinate (*rac*-**2-A**) (125 MHz, CDCl$_3$).

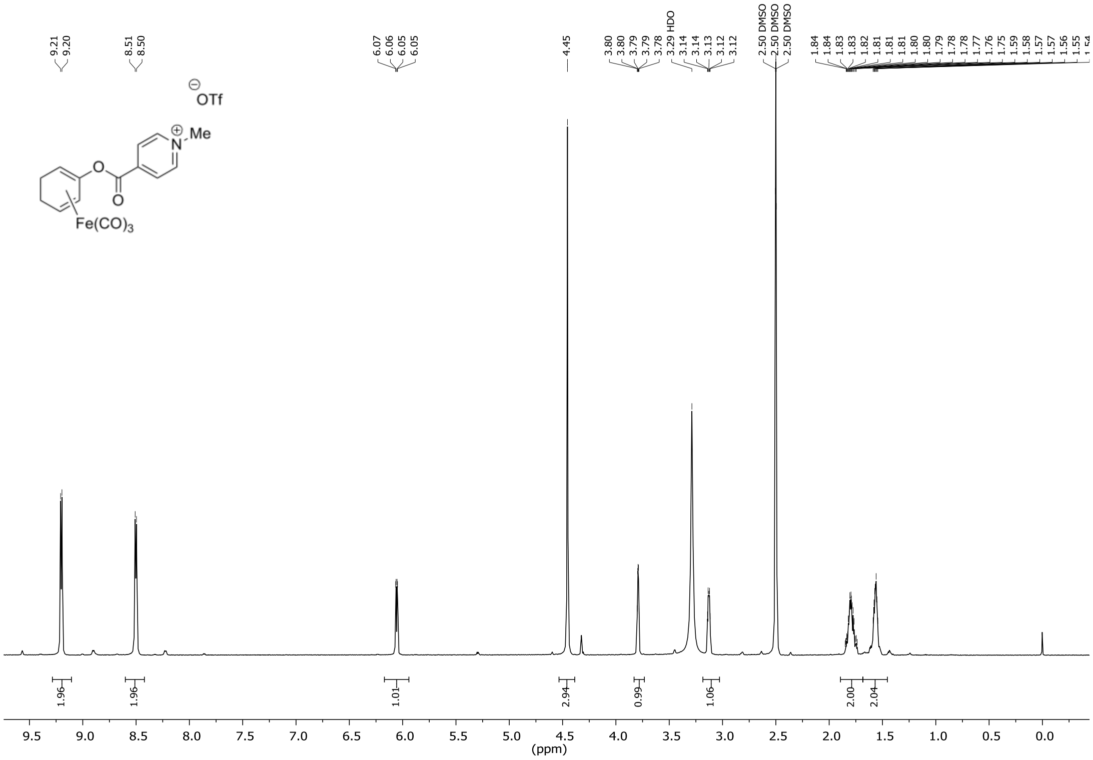

Figure S 3: ^{1}H NMR of Mito-CORM 1-A (500 MHz, DMSO-d_6).

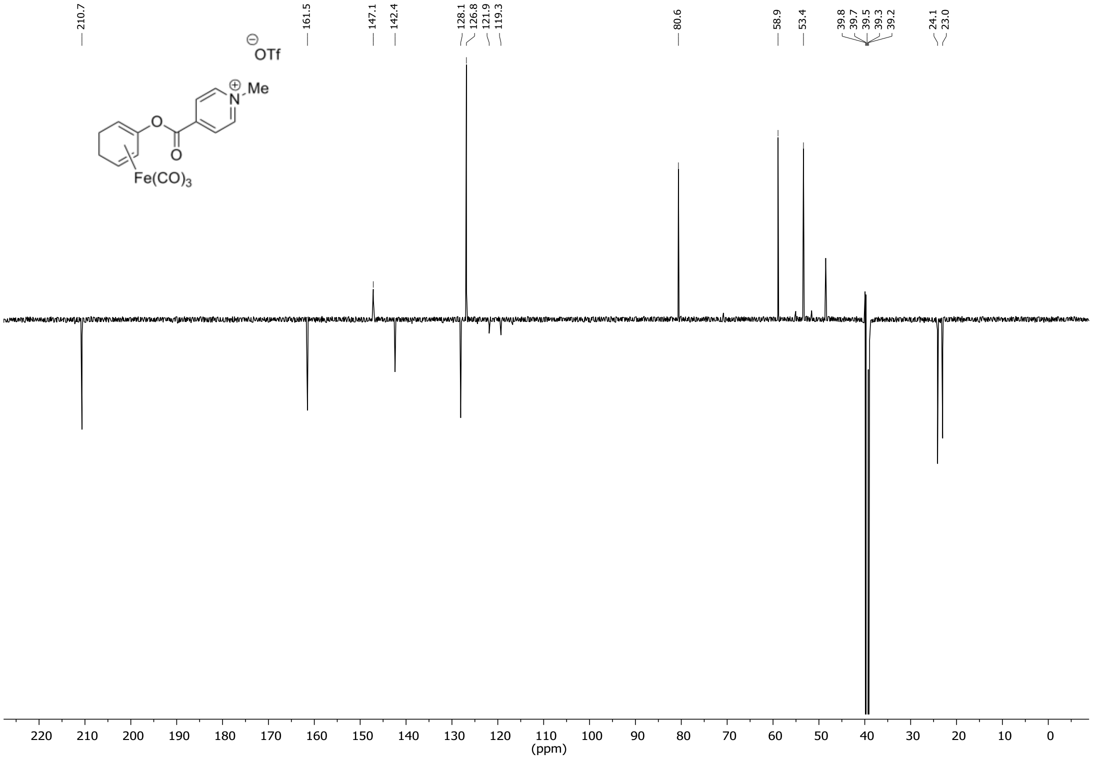

Figure S 4: ^{13}C NMR of Mito-CORM 1-A (125 MHz, DMSO-d_6).

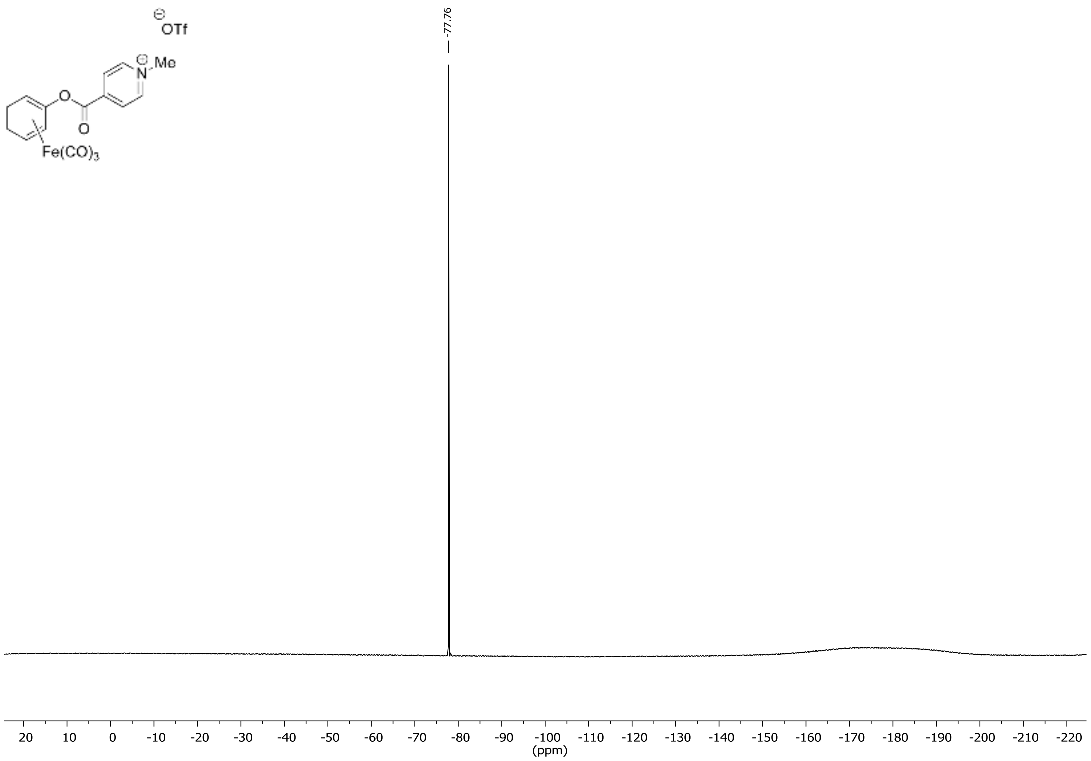

Figure S 5: ^{19}F NMR of Mito-CORM 1-A (471 MHz, DMSO-d$_6$).

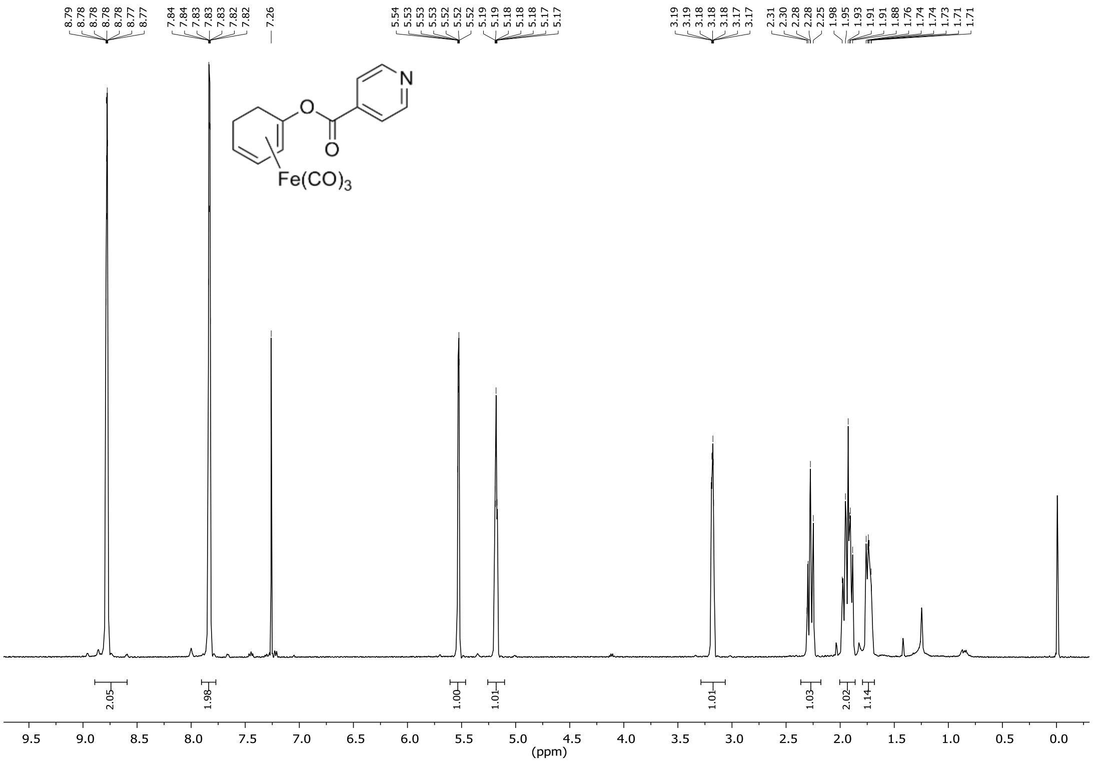

Figure S 6: ^{1}H NMR of (1,3-cyclohexadiene-Fe(CO)$_3$)-1-yl isonicotinate (*rac*-**2-B**) (500 MHz, CDCl$_3$).

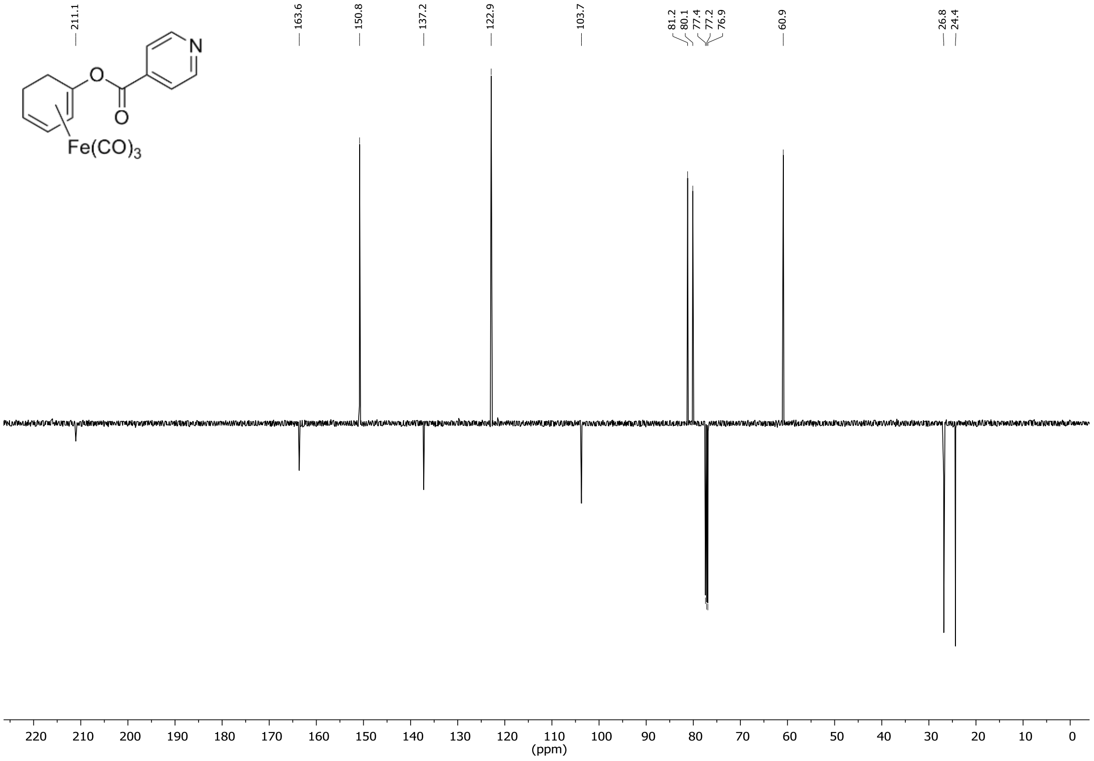

Figure S 7: ^{13}C NMR of (1,3-cyclohexadiene-$Fe(CO)_3$)-1-yl isonicotinate (*rac*-**2-B**) (125 MHz, $CDCl_3$).

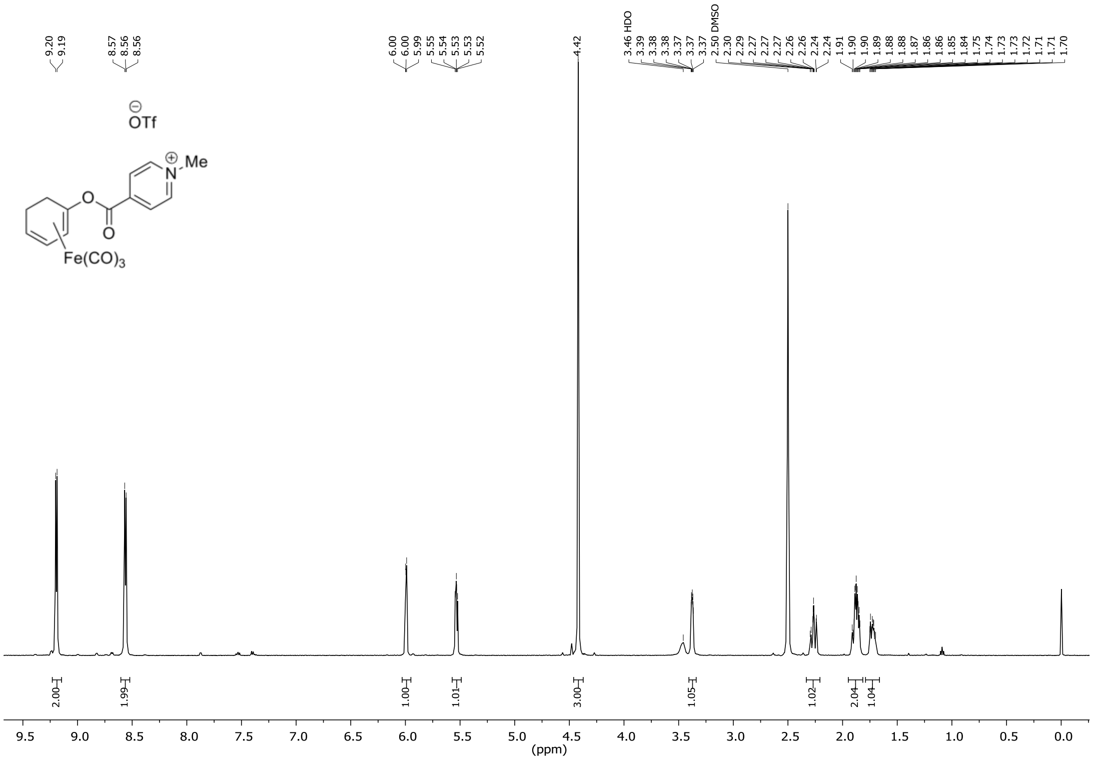

Figure S 8: ^{1}H NMR of Mito-CORM 1-B (125 MHz, DMSO-d_6).

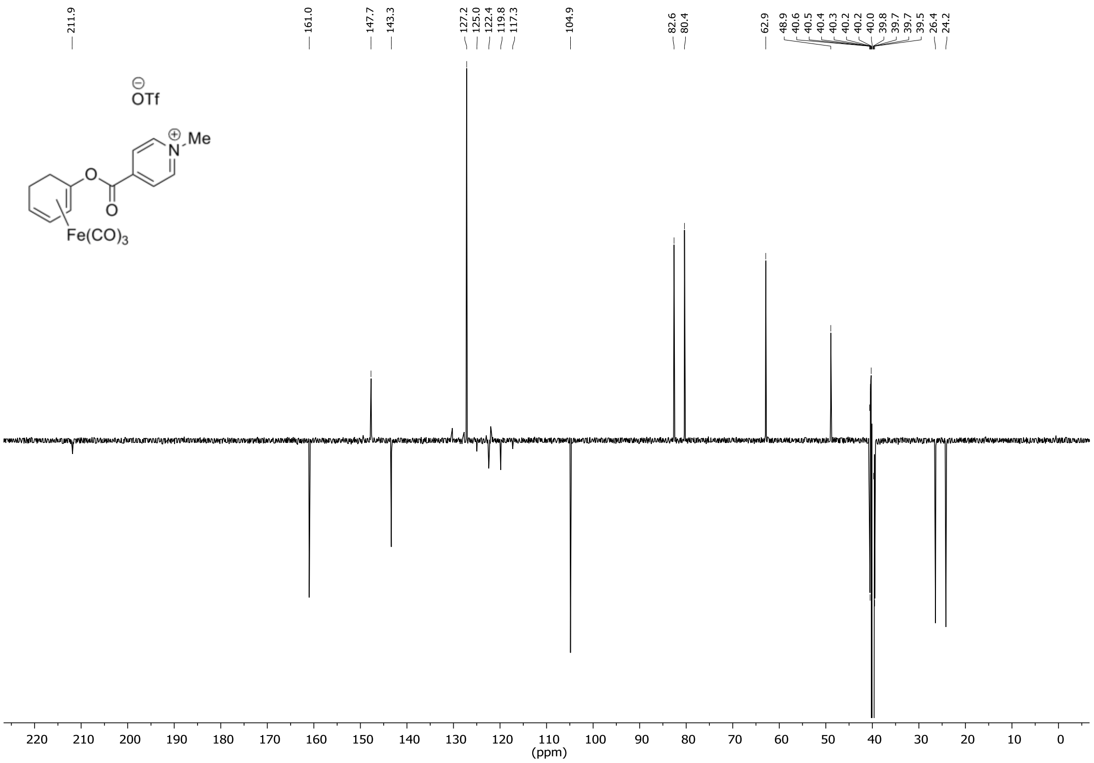

Figure S 9: ^{13}C NMR of Mito-CORM 1-B (125 MHz, DMSO-d_6).

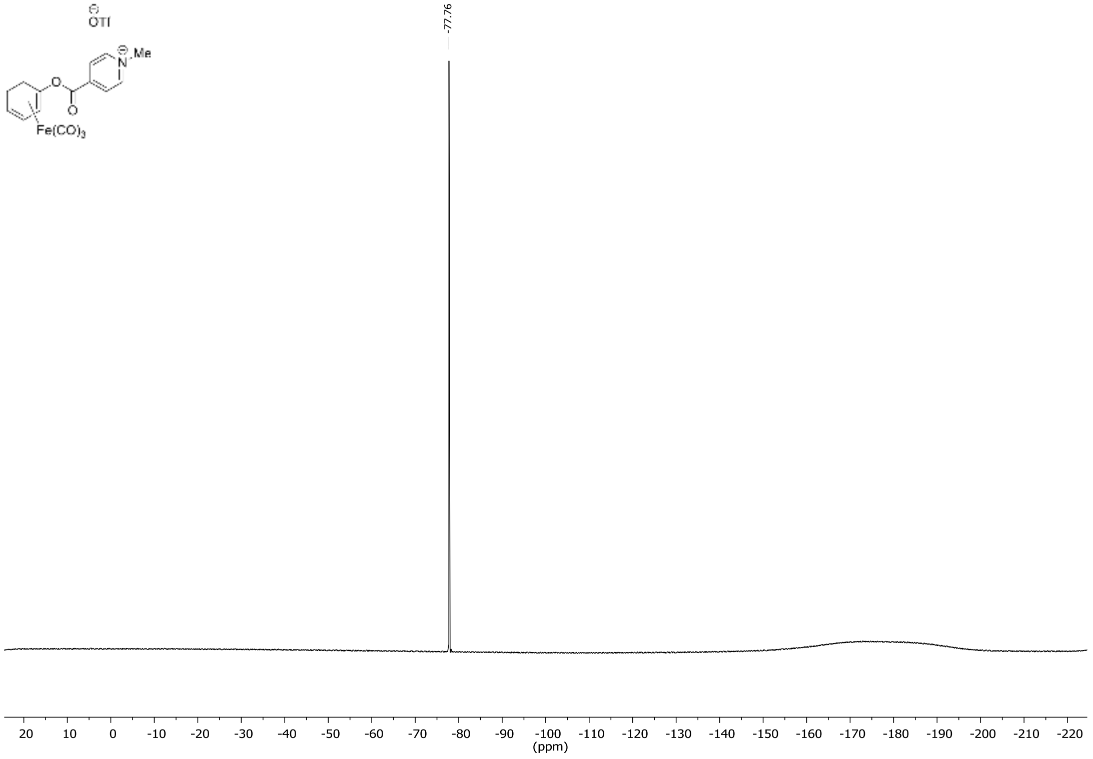

Figure S 10: ^{19}F NMR of Mito-CORM 1-B (471 MHz, DMSO-d_6).

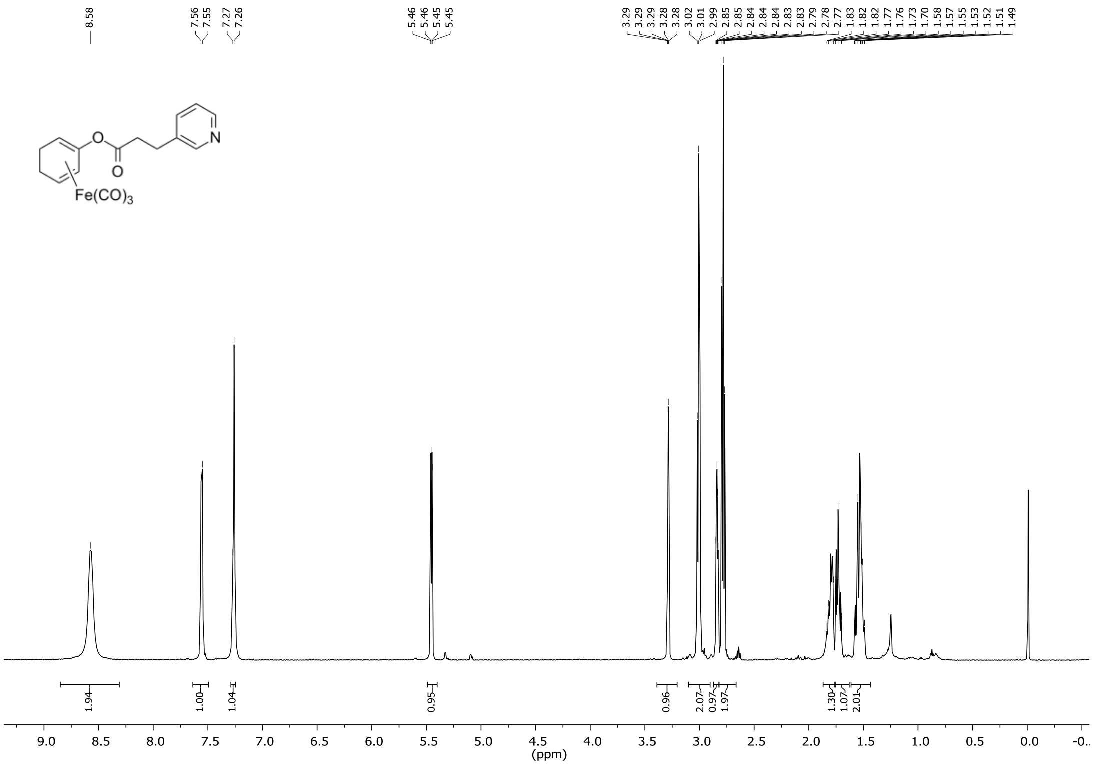

Figure S 11: ^{1}H NMR of (1,5-cyclohexadiene-Fe(CO)$_3$)-1-yl-3-pyridine propionate (*rac*-**6**-**A**) (600 MHz, $CDCl_3$)

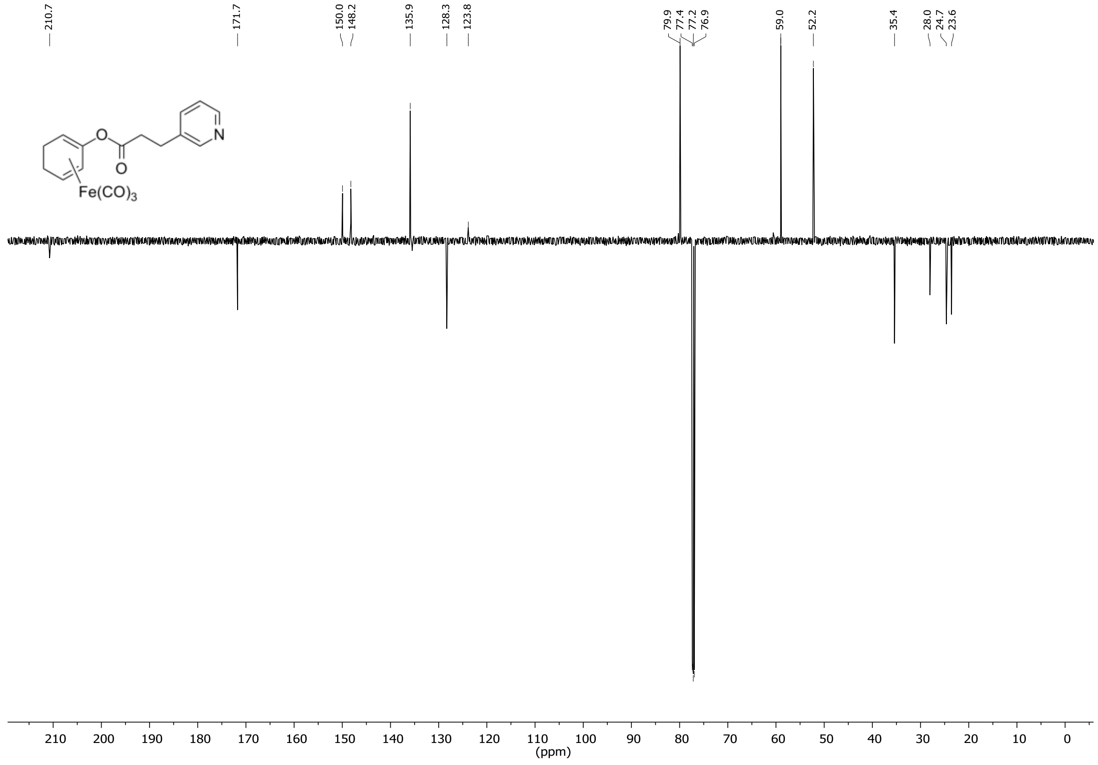

Figure S 12: ^{13}C NMR of (1,5-cyclohexadiene-Fe(CO)$_3$)-1-yl-3-pyridine propionate (*rac*-**6**-**A**) (150 MHz, $CDCl_3$).

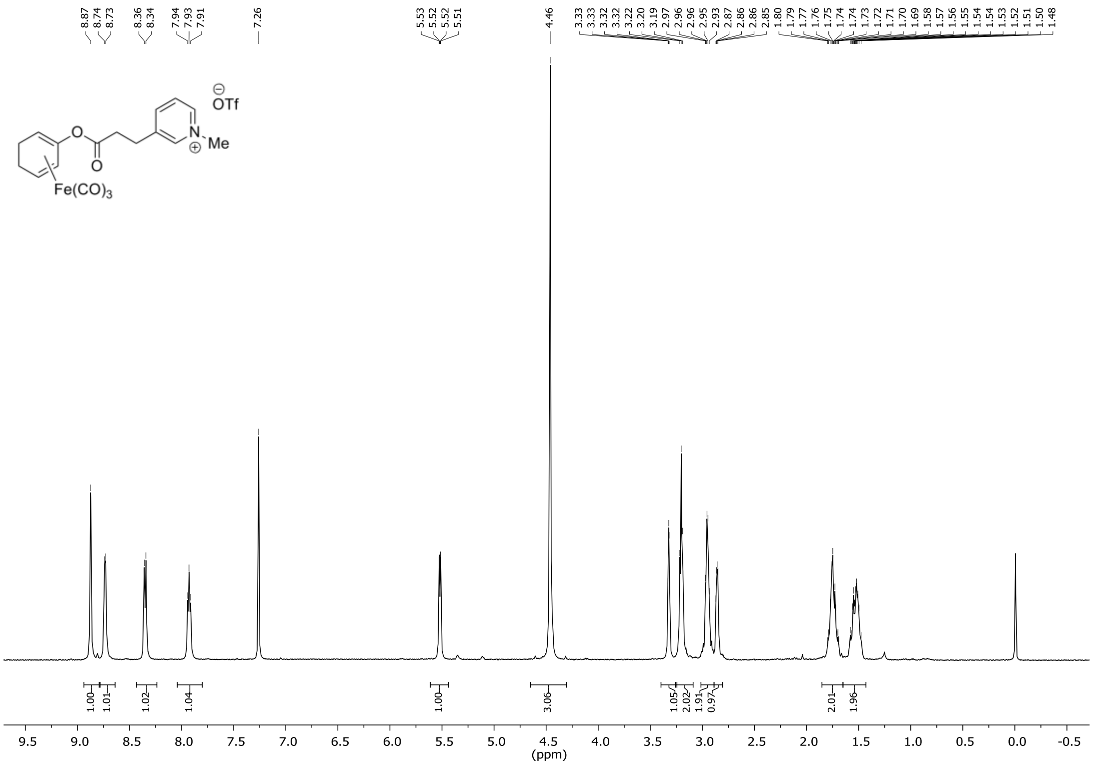

Figure S 13: ^{1}H NMR of **Mito-CORM 2-A** (500 MHz, $CDCl_3$).

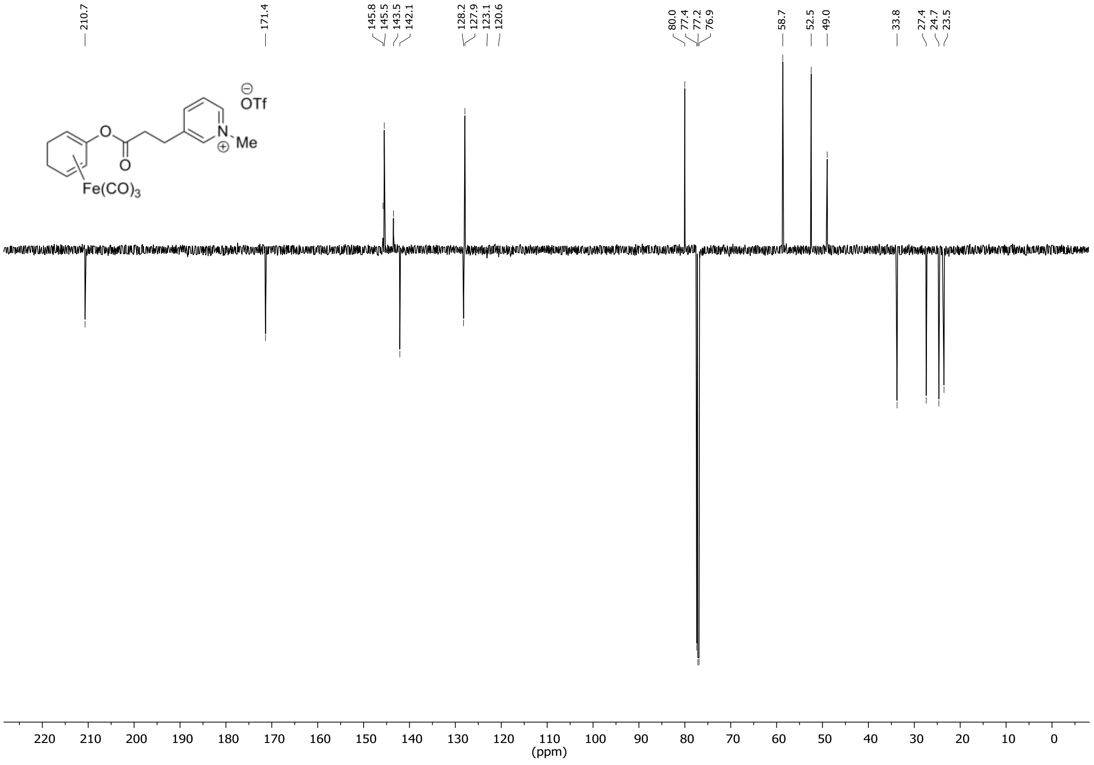

Figure S 14: ^{13}C NMR of **Mito-CORM 2-A** (125 MHz, $CDCl_3$).

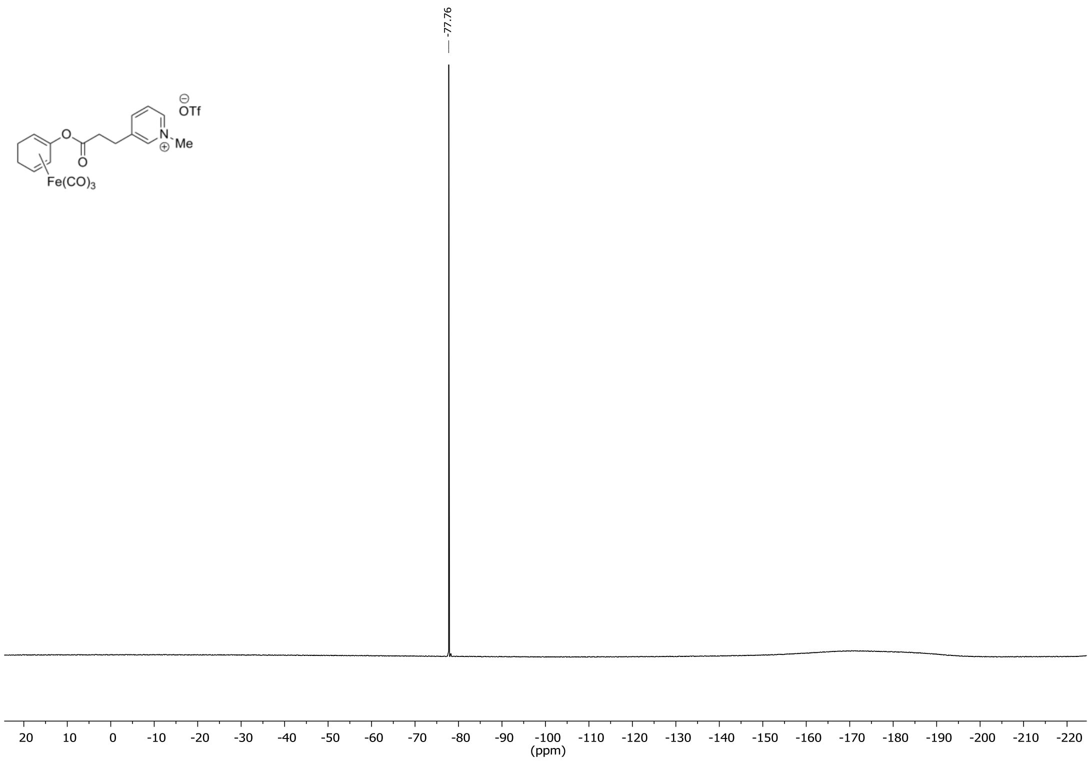

Figure S 15: ^{19}F NMR of **Mito-CORM 2-A** (471 MHz, DMSO-d6).

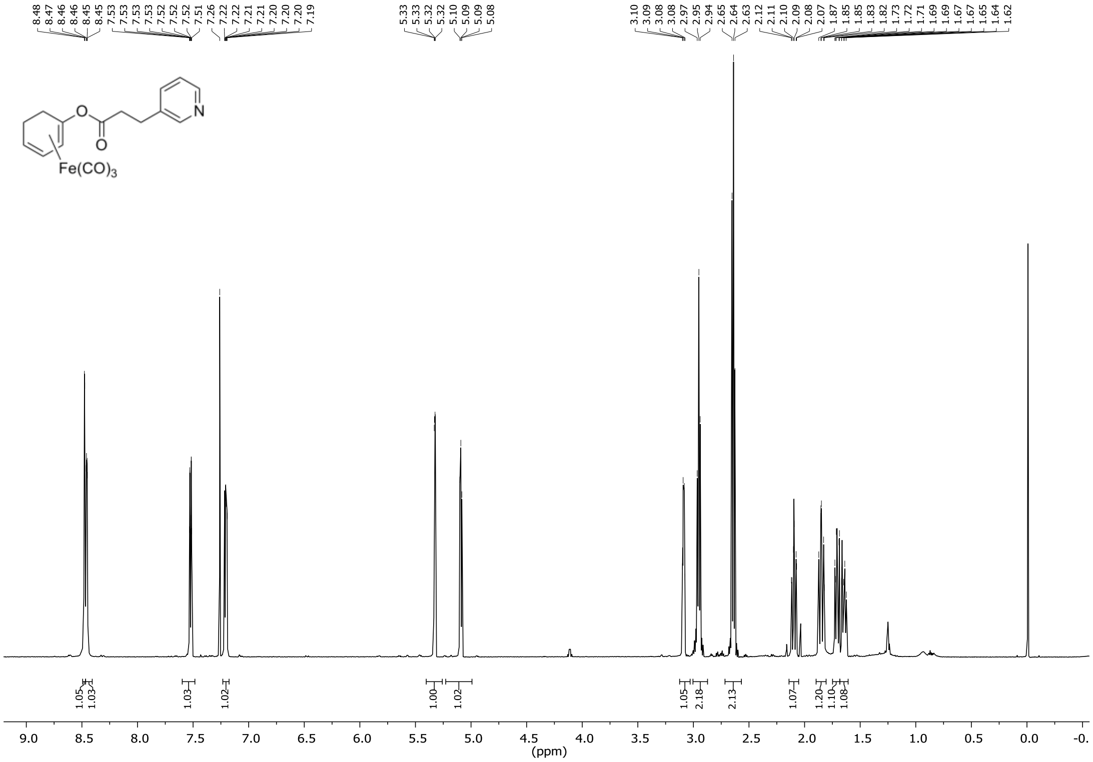

Figure S 16: ^{1}H NMR of (1,3-Cyclohexadiene-Fe(CO)3)-1-yl-3-pyridine propionate (*rac*-**6-B**) (600 MHz, CDCl3)

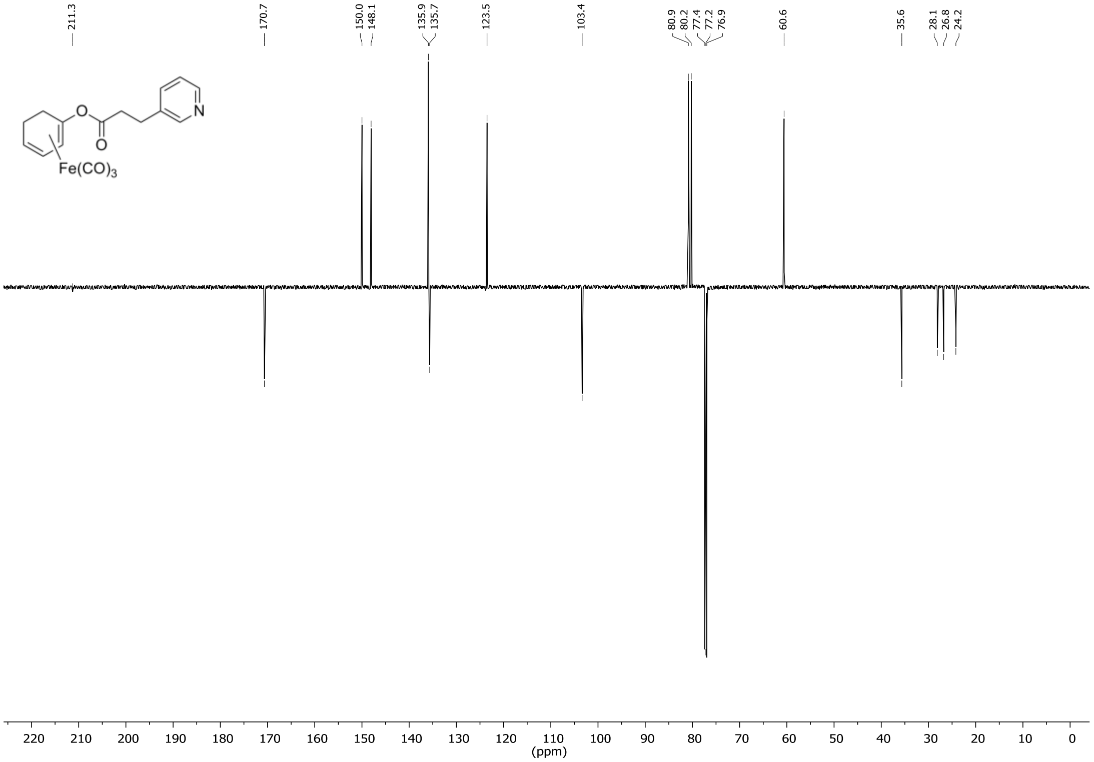

Figure S 17: ^{13}C NMR of ^{1}H NMR of (1,3-Cyclohexadien-Fe(CO)$_3$)-1-yl-3-pyridine propionate (*rac*-**6-B**) (150 MHz, $CDCl_3$).

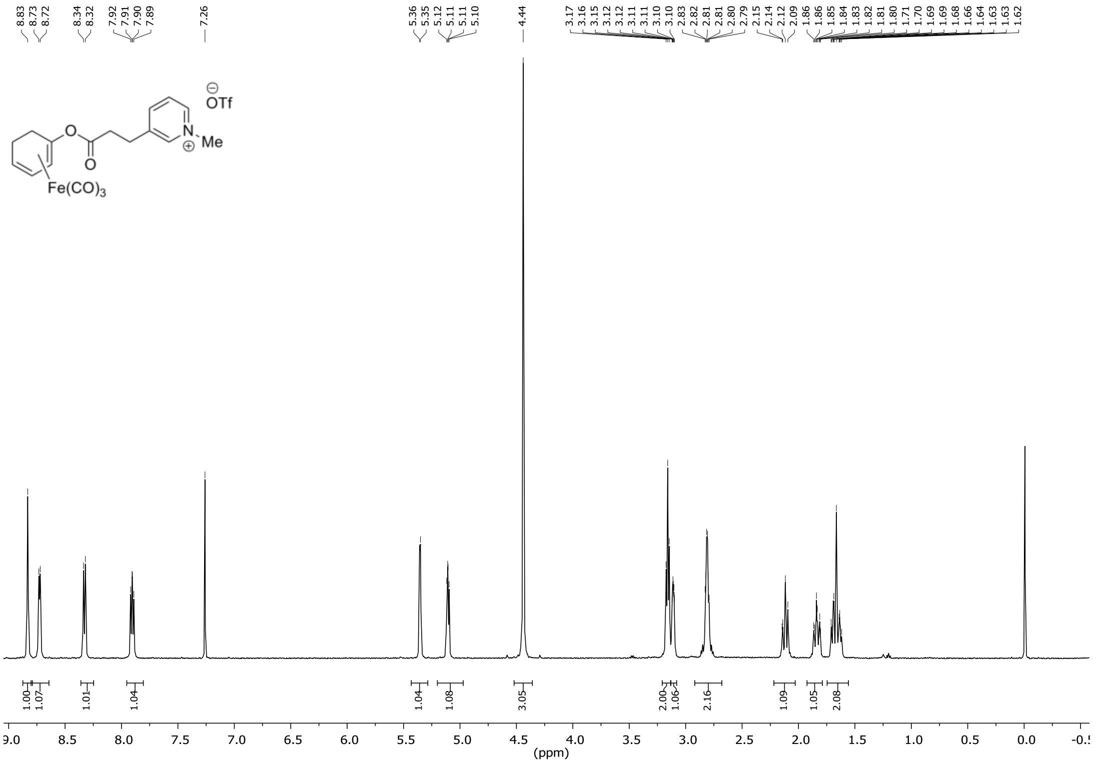

Figure S 18: ^{1}H NMR of **Mito-CORM 2-B** (500 MHz, $CDCl_3$).

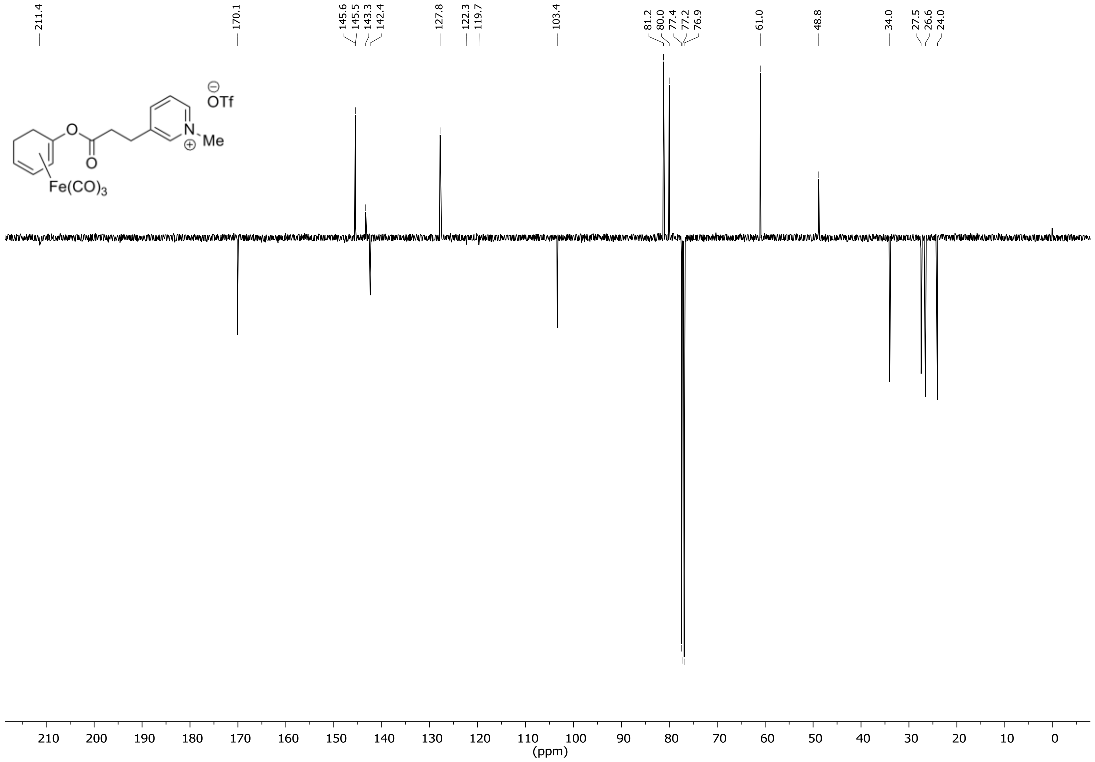

Figure S 19: ^{13}C NMR of **Mito-CORM 2-B** (125 MHz, $CDCl_3$).

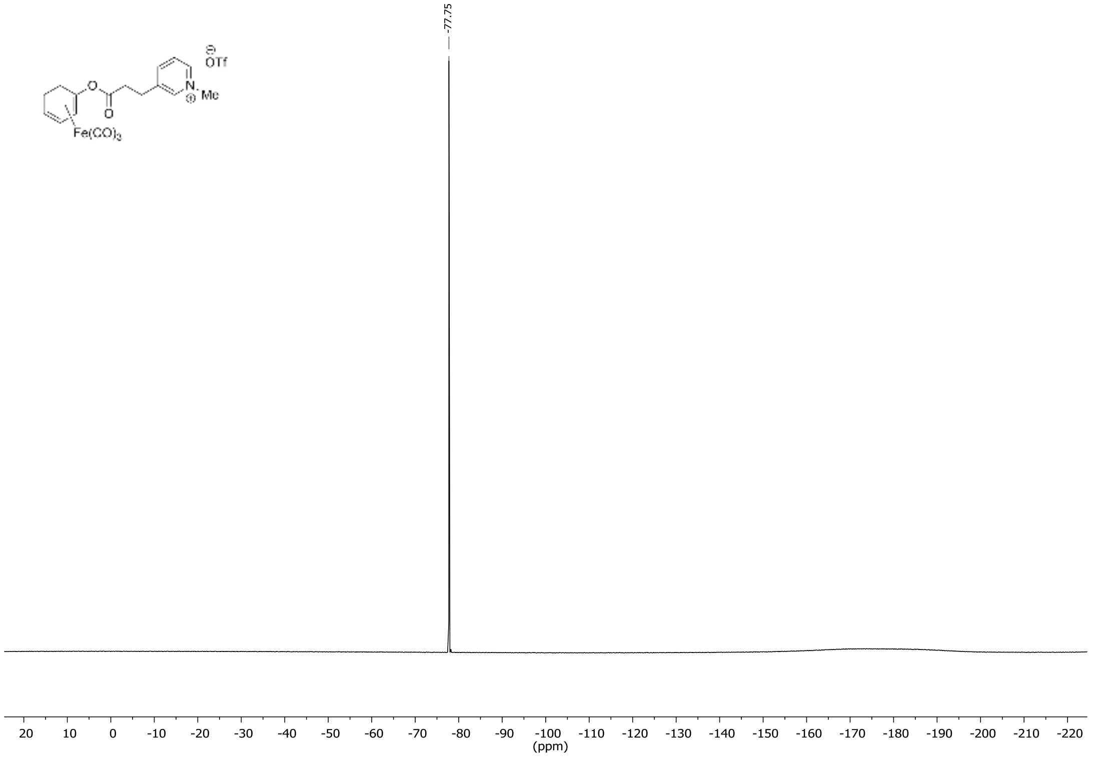

Figure S 20: ^{19}F NMR of **Mito-CORM 2-B** (471 MHz, DMSO-d_6).

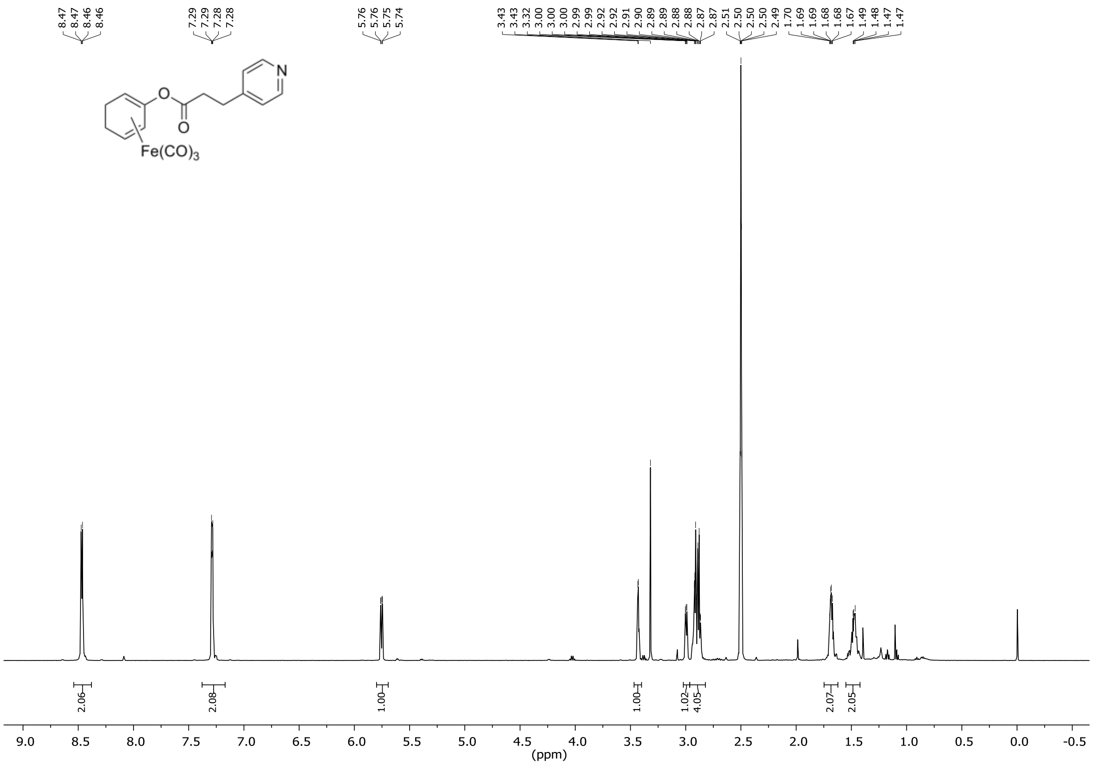

Figure S 21: ^{1}H NMR of (1,5-Cyclohexadien-Fe(CO)3)-1-yl-4-pyridine propionate (*rac*-**8**-**A**) (500 MHz, DMSO-d6).

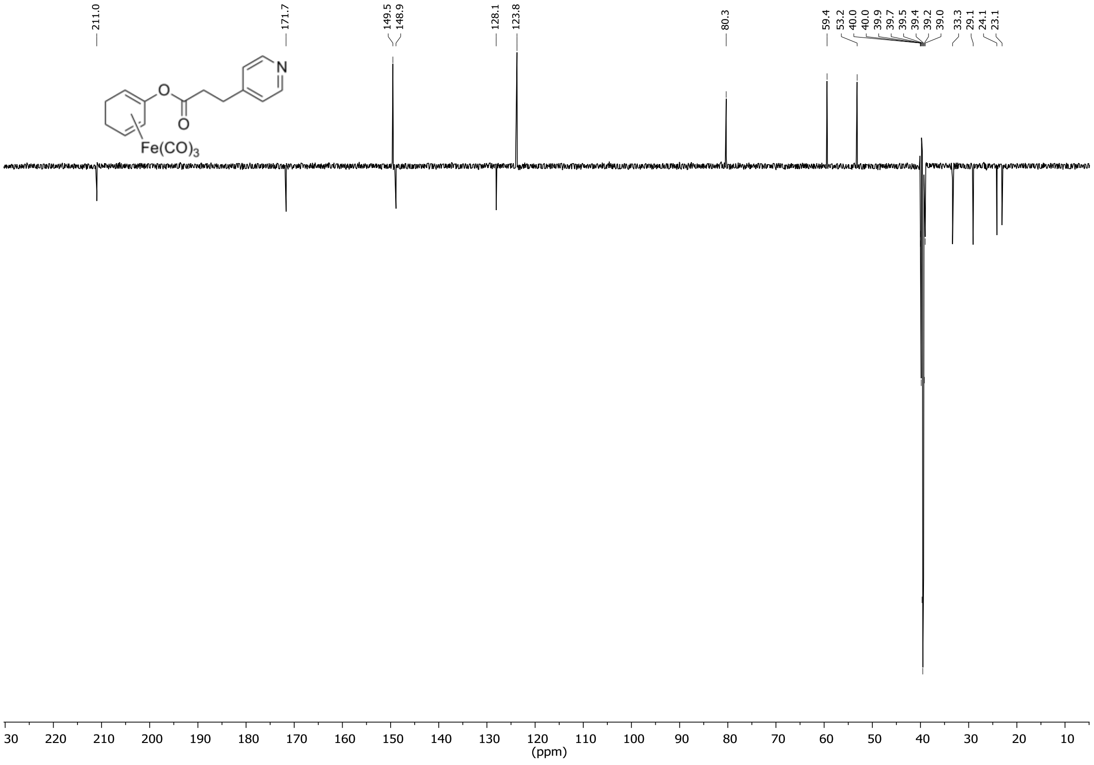

Figure S 22: ^{13}C NMR of (1,5-Cyclohexadien-Fe(CO)3)-1-yl-4-pyridine propionate (*rac*-**8**-**A**) (125 MHz, DMSO-d6).

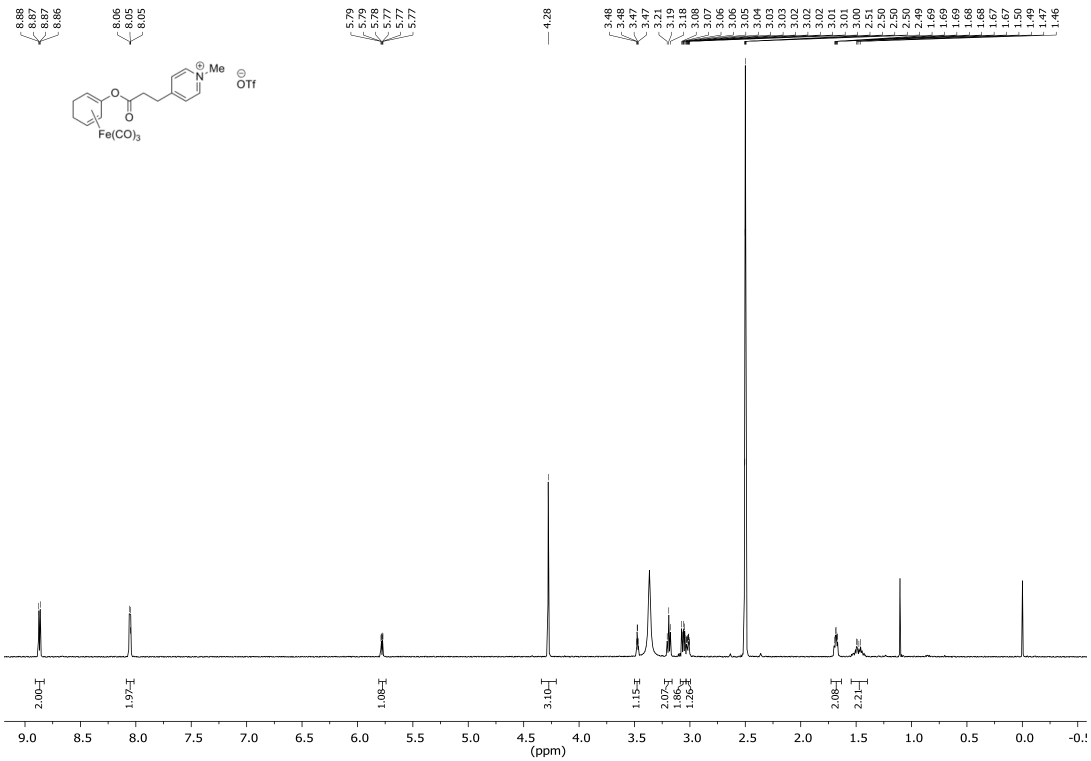

Figure S 23: ^{1}H NMR of **Mito-CORM 3-A** (500 MHz, DMSO-d$_6$).

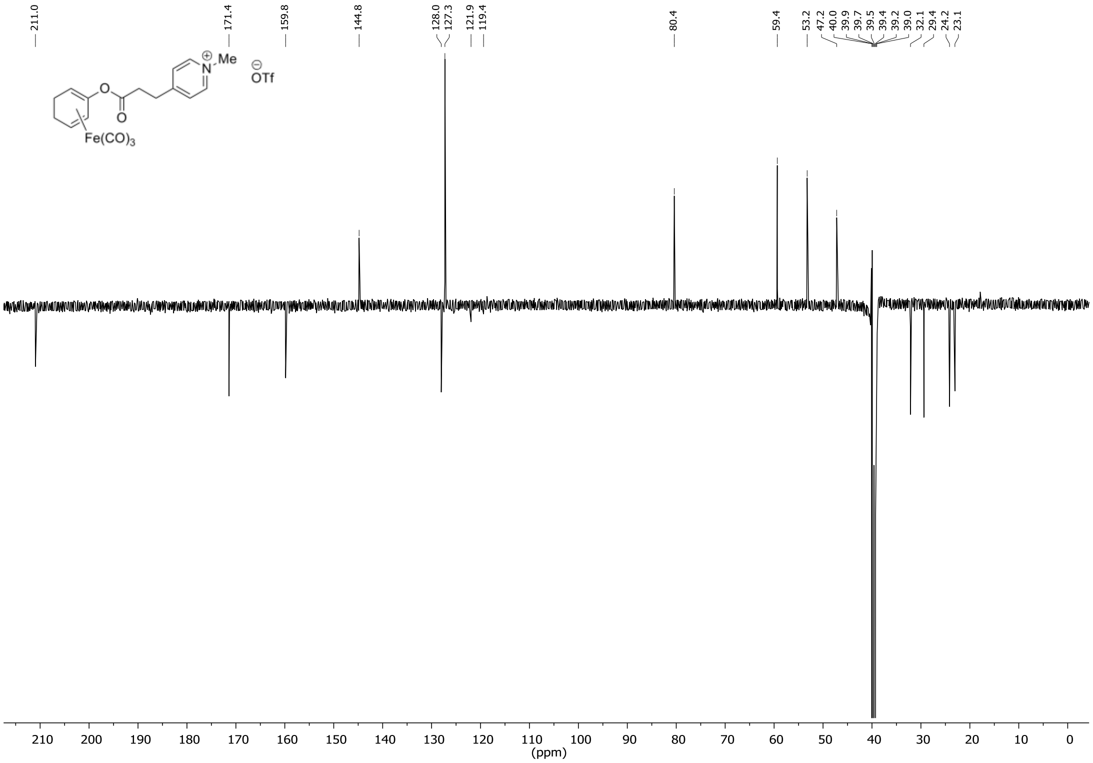

Figure S 24: ^{13}C NMR of **Mito-CORM 3-A** (125 MHz, DMSO-d$_6$).

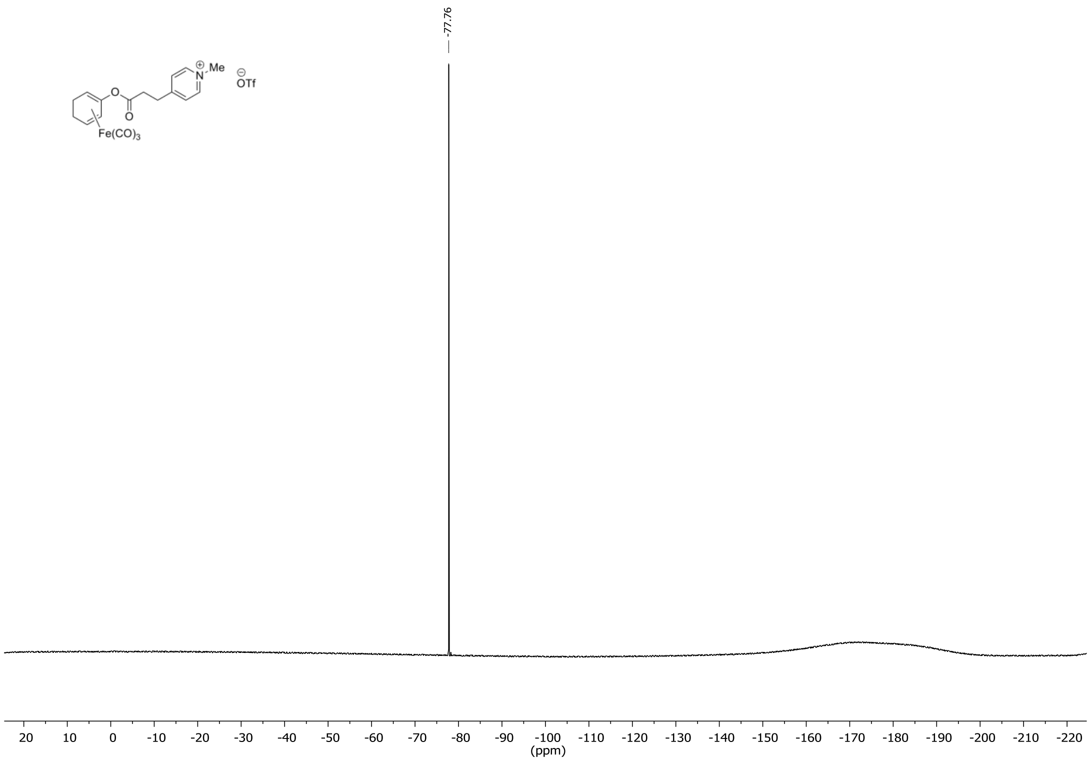

Figure S 25: ^{19}F NMR of **Mito-CORM 3-A** (471 MHz, DMSO-d_6).

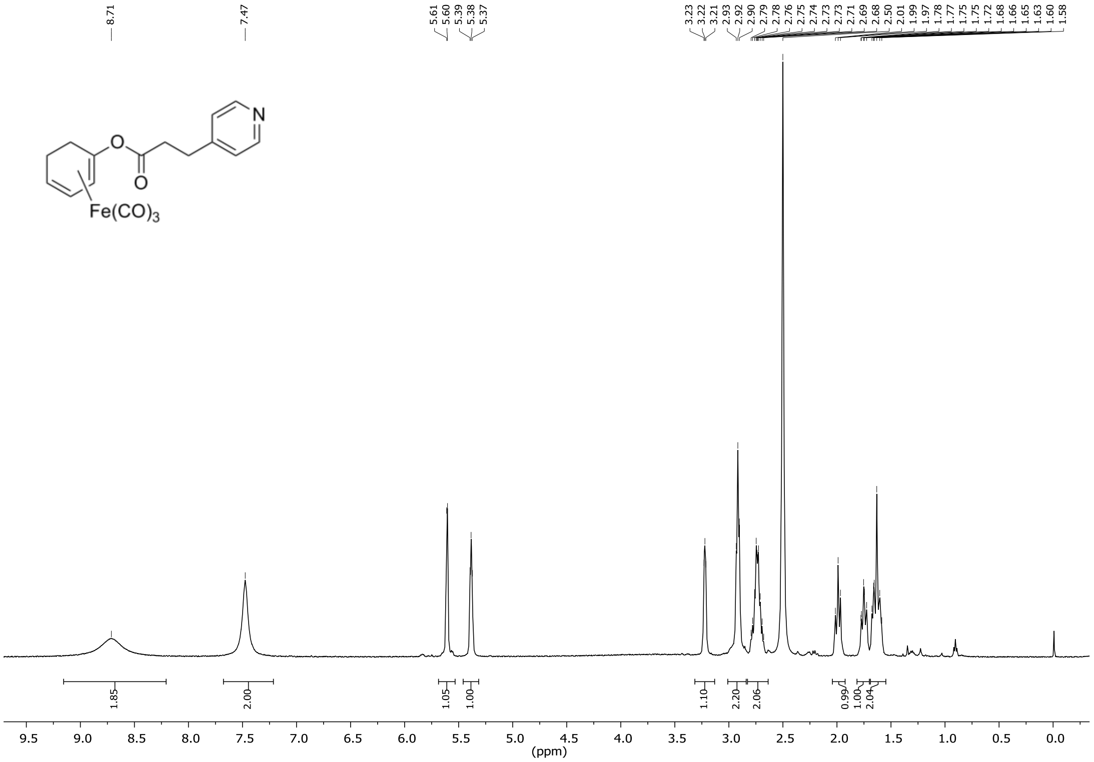

Figure S 26: ^{1}H NMR of (1,3-Cyclohexadien-Fe(CO)3)-1-yl-3-pyridine propionate (*rac*-**8-B**) (500 MHz, DMSO-d_6).

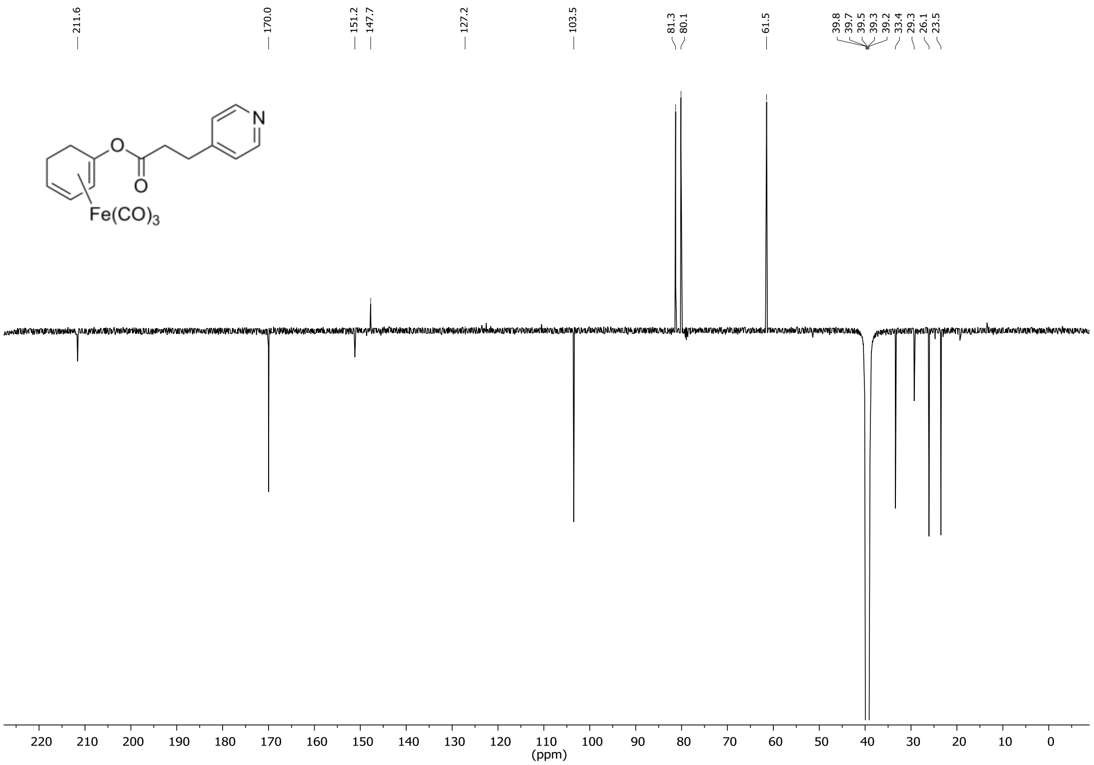

Figure S 27: ^{13}C NMR of (1,3-Cyclohexadien-Fe(CO)$_3$)-1-yl-3-pyridine propionate (*rac*-**8-B**) (125 MHz, DMSO-d$_6$).

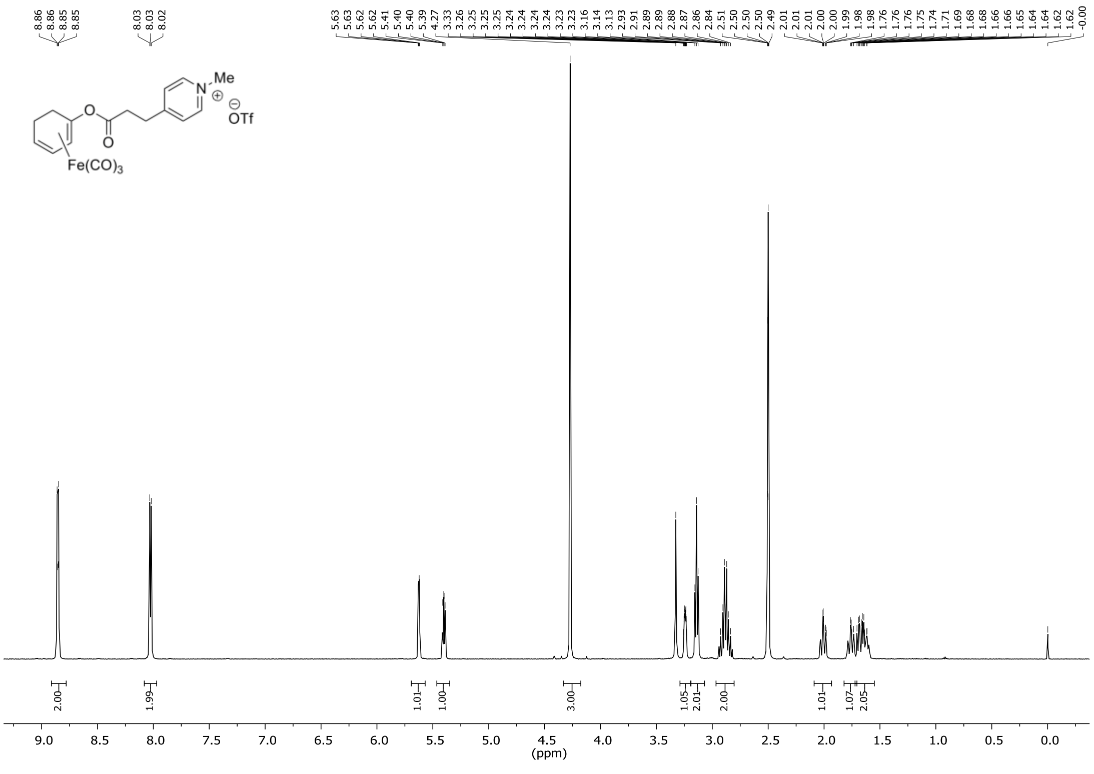

Figure S 28: ^{1}H NMR of **Mito-CORM 3-B** (500 MHz, DMSO-d$_6$).

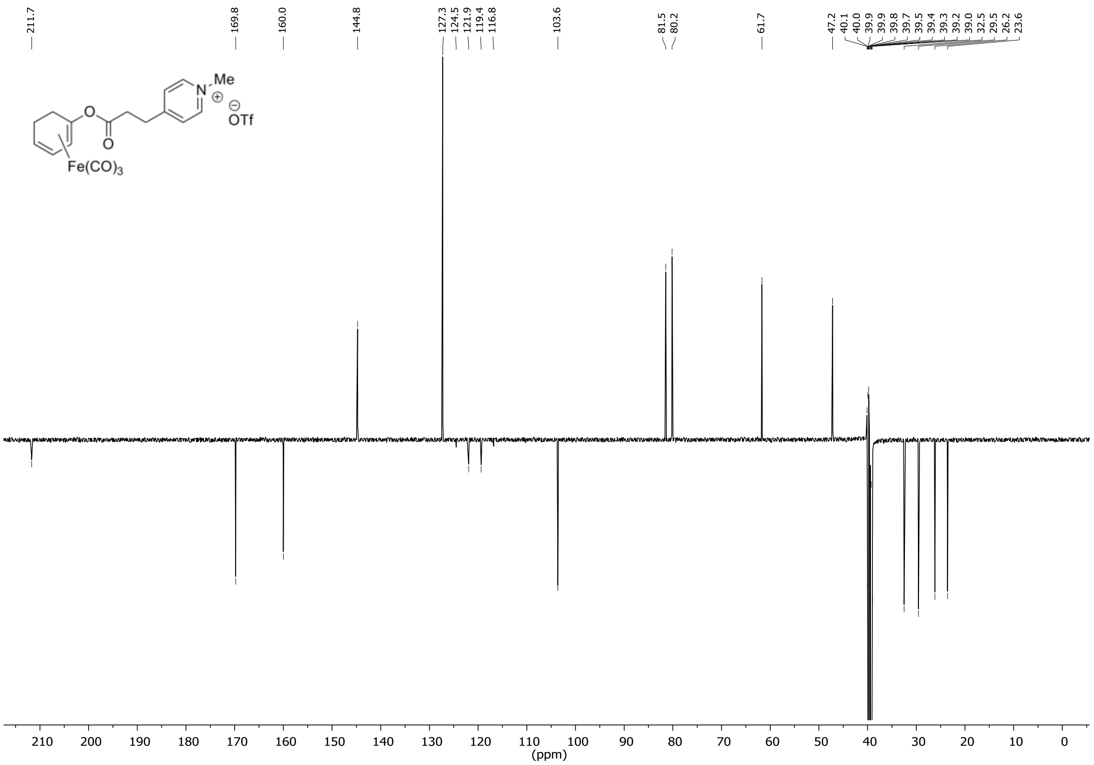

Figure S 29: ^{13}C NMR of **Mito-CORM 3-B** (125 MHz, DMSO-d_6).

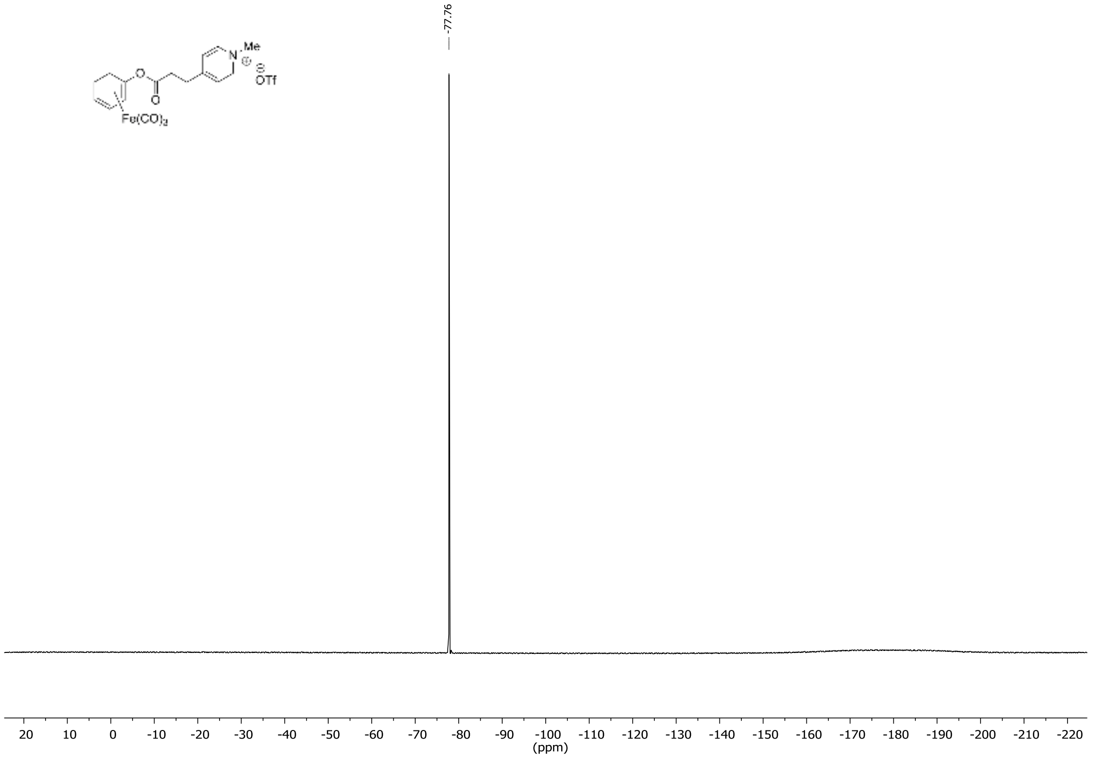

Figure S 30: ^{19}F NMR of **Mito-CORM 3-B** (471 MHz, DMSO-d_6).

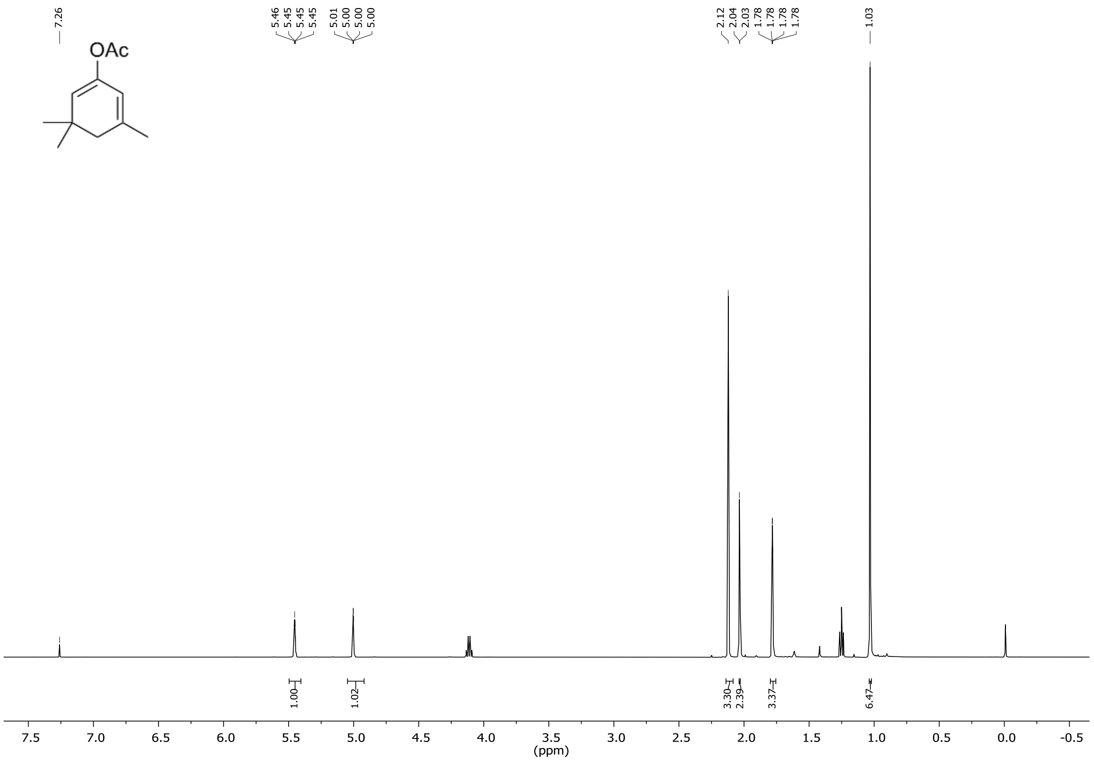

Figure S 31: ^{1}H NMR of 3,3,5-Trimethylcyclohexa-1,5-dien-1-yl acetate (**10**) (500 MHz, $CDCl_3$)

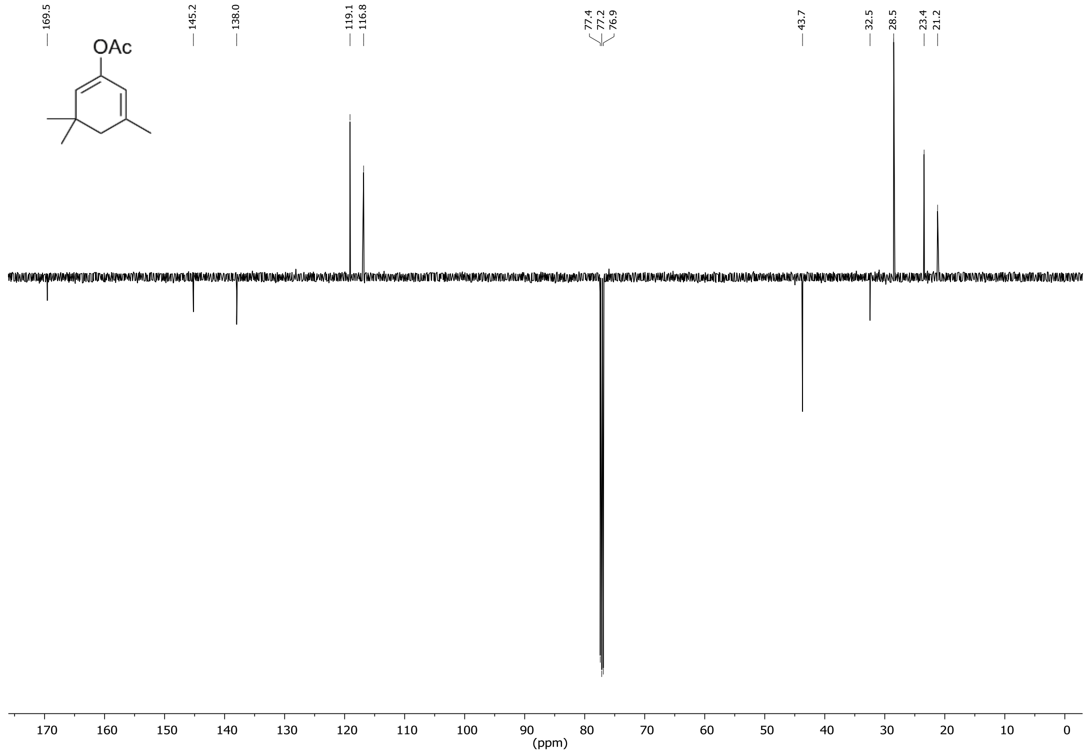

Figure S 32: ^{13}C NMR of 3,3,5-Trimethylcyclohexa-1,5-dien-1-yl acetate (**10**) (125 MHz, $CDCl_3$).

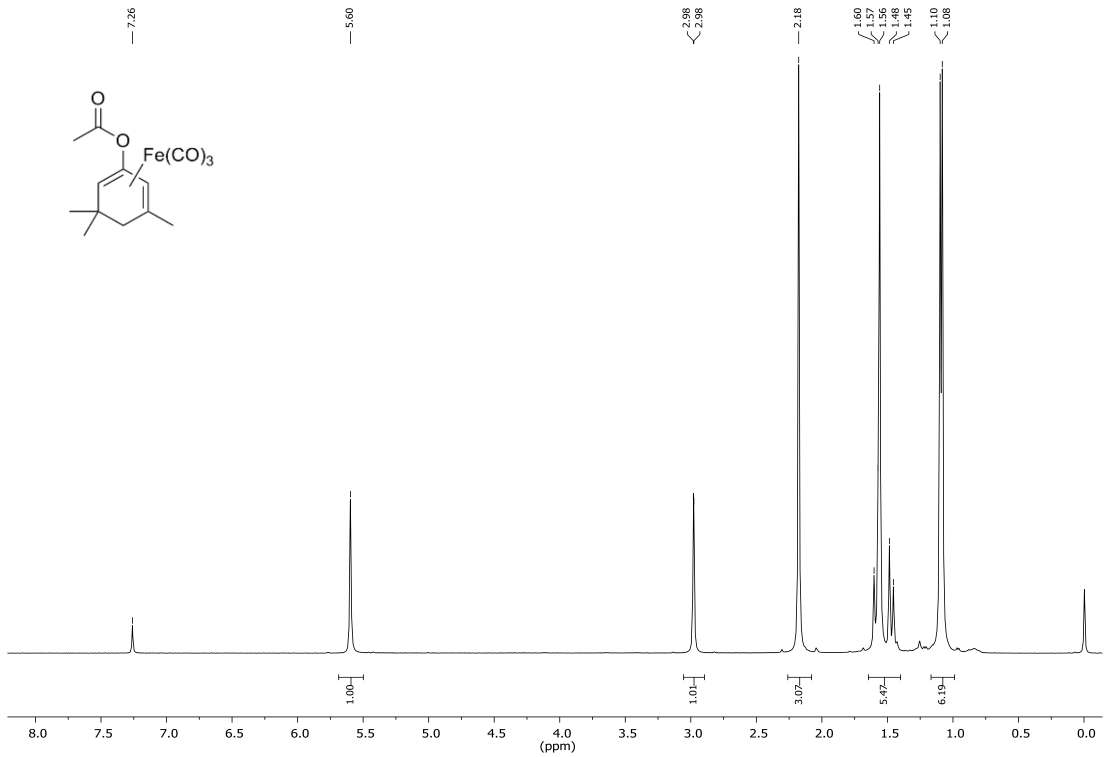

Figure S 33: ^{1}H NMR of (3,3,5-Trimethylcyclohexa-1,5-diene-Fe(CO)$_3$)-1-yl acetate (rac-**11-A**) (500 MHz, $CDCl_3$)

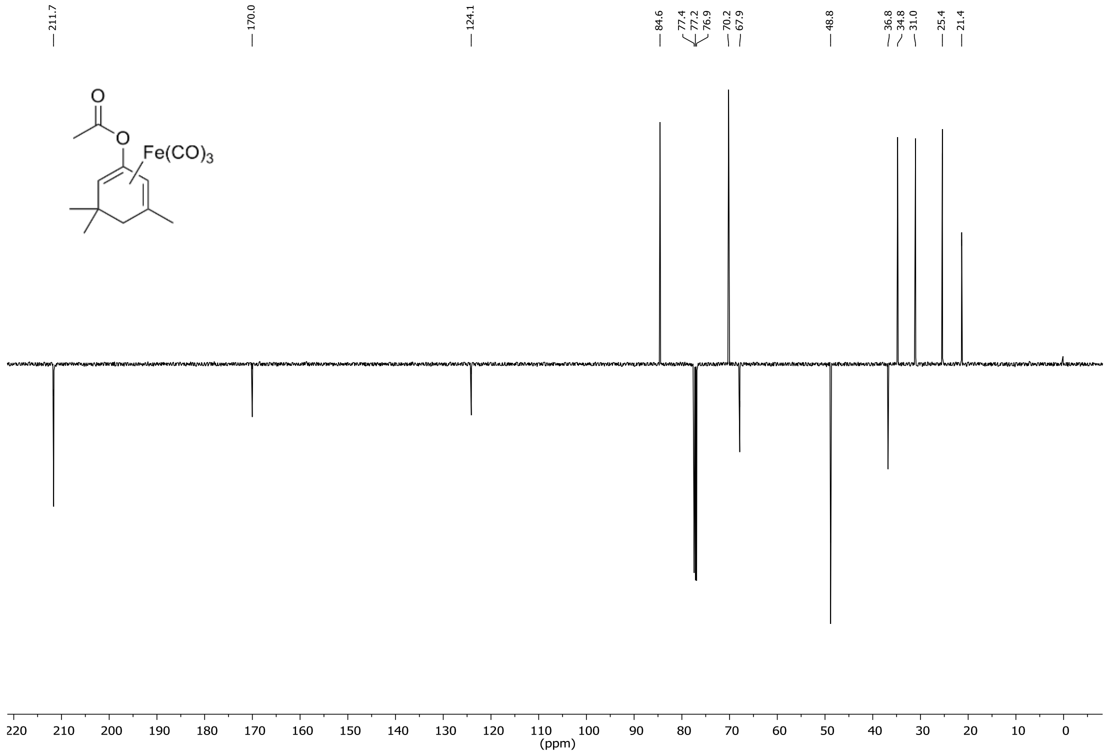

Figure S 34: ^{13}C NMR of (3,3,5-Trimethylcyclohexa-1,5-diene-Fe(CO)$_3$)-1-yl acetate (rac-**11-A**) (125 MHz, $CDCl_3$).

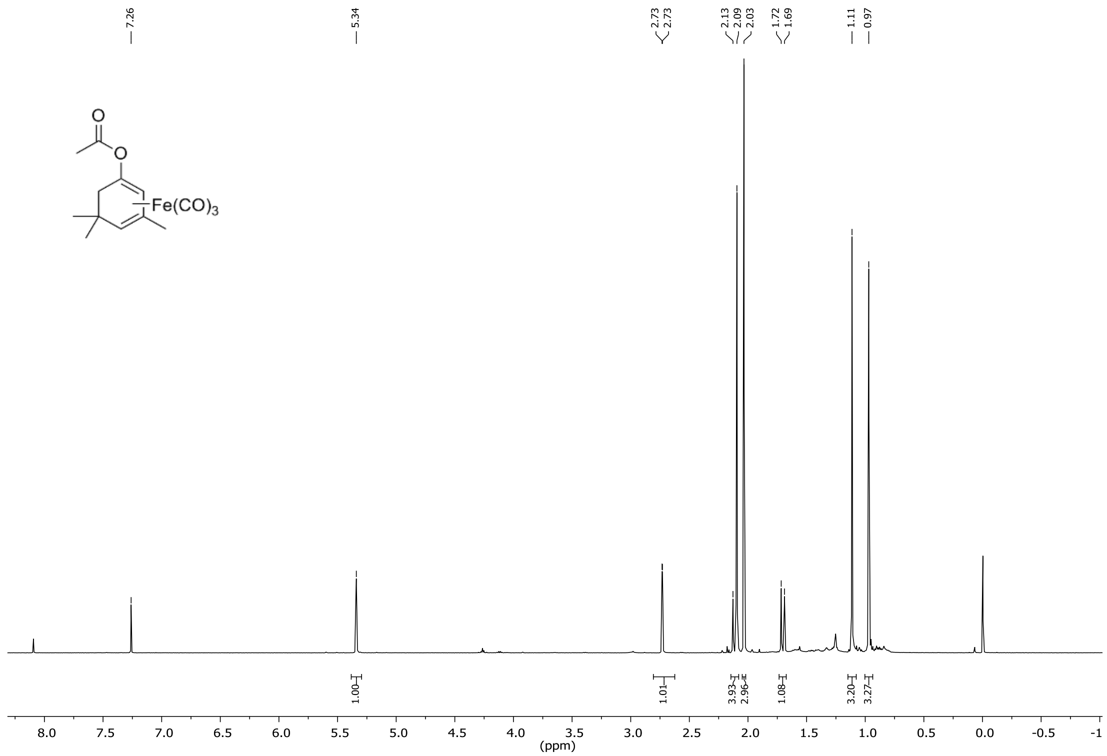

Figure S 35: ^{1}H NMR of (3,3,5-Trimethylcyclohexa-1,3-diene-$Fe(CO)_3$)-1-yl acetate (rac-**11**-**B**) (500 MHz, $CDCl_3$)

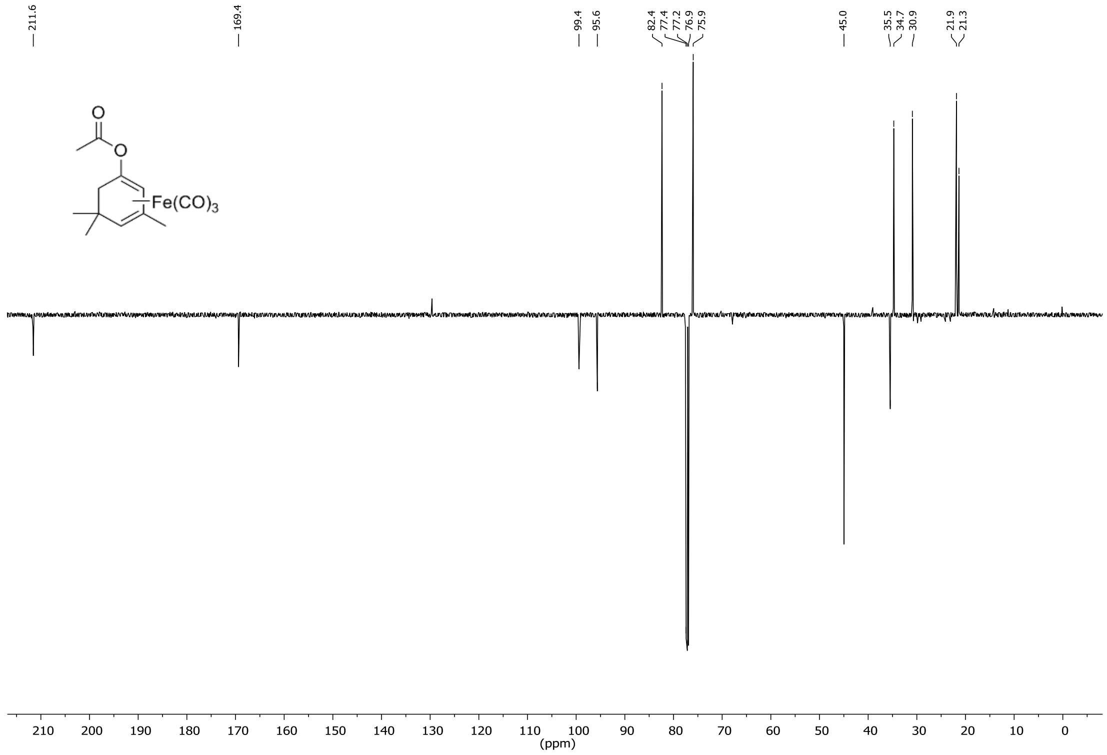

Figure S 36: ^{13}C NMR of (3,3,5-Trimethylcyclohexa-1,3-diene-$Fe(CO)_3$)-1-yl acetate (rac-**11**-**B**) (125 MHz, $CDCl_3$).

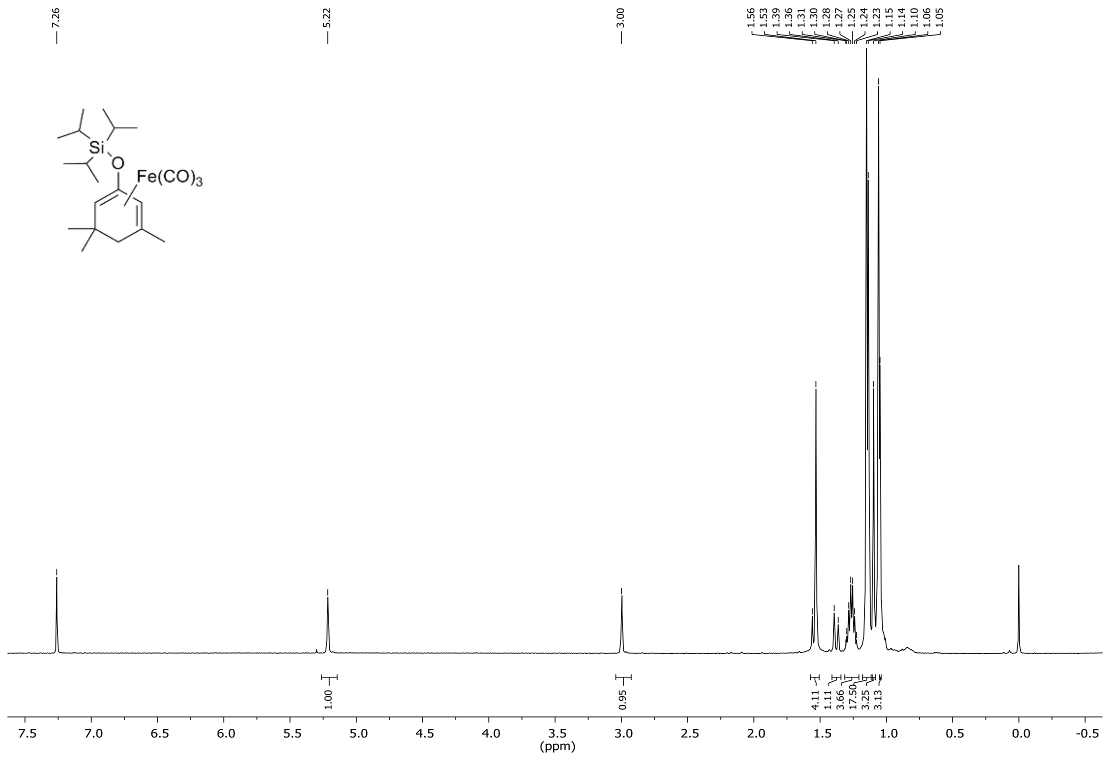

Figure S 37: ^{1}H NMR of 1-Tri*iso*propylsiloxy-3,3,5-trimethyl-1,5-cyclohexadiene-(Fe(CO)$_3$) (rac-**12-A**) (500 MHz, $CDCl_3$)

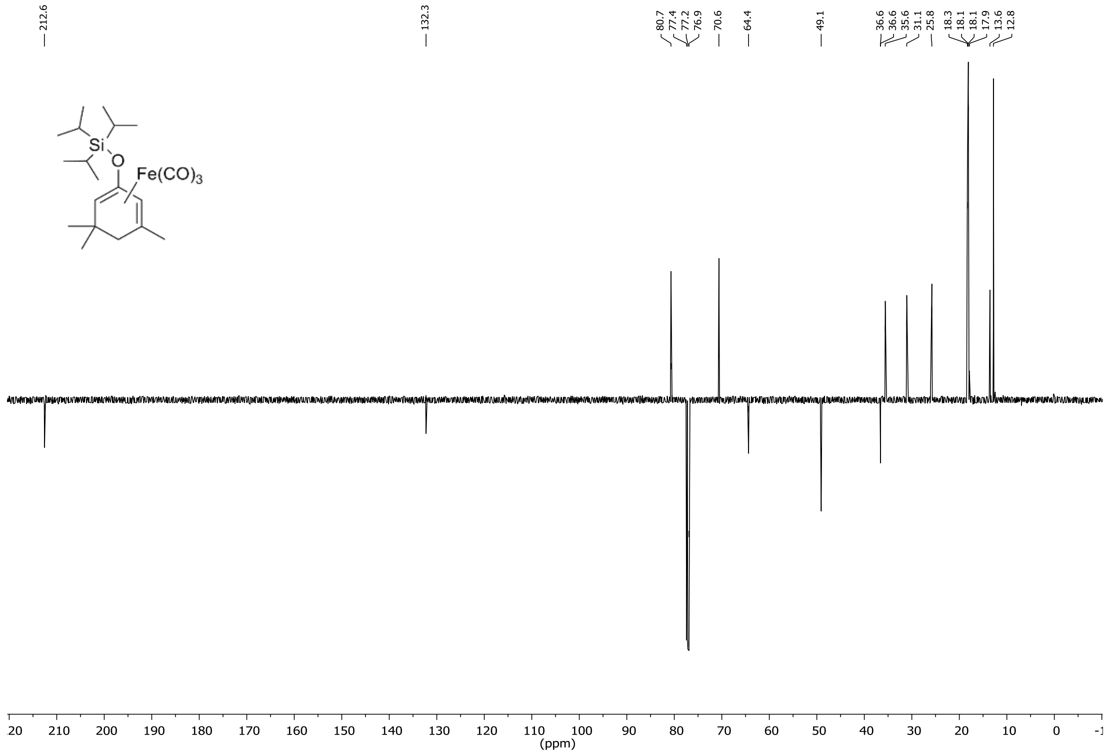

Figure S 38: ^{13}C NMR of 1-Tri*iso*propylsiloxy-3,3,5-trimethyl-1,5-cyclohexadiene-(Fe(CO)$_3$) (rac-**12-A**) (125 MHz, $CDCl_3$).

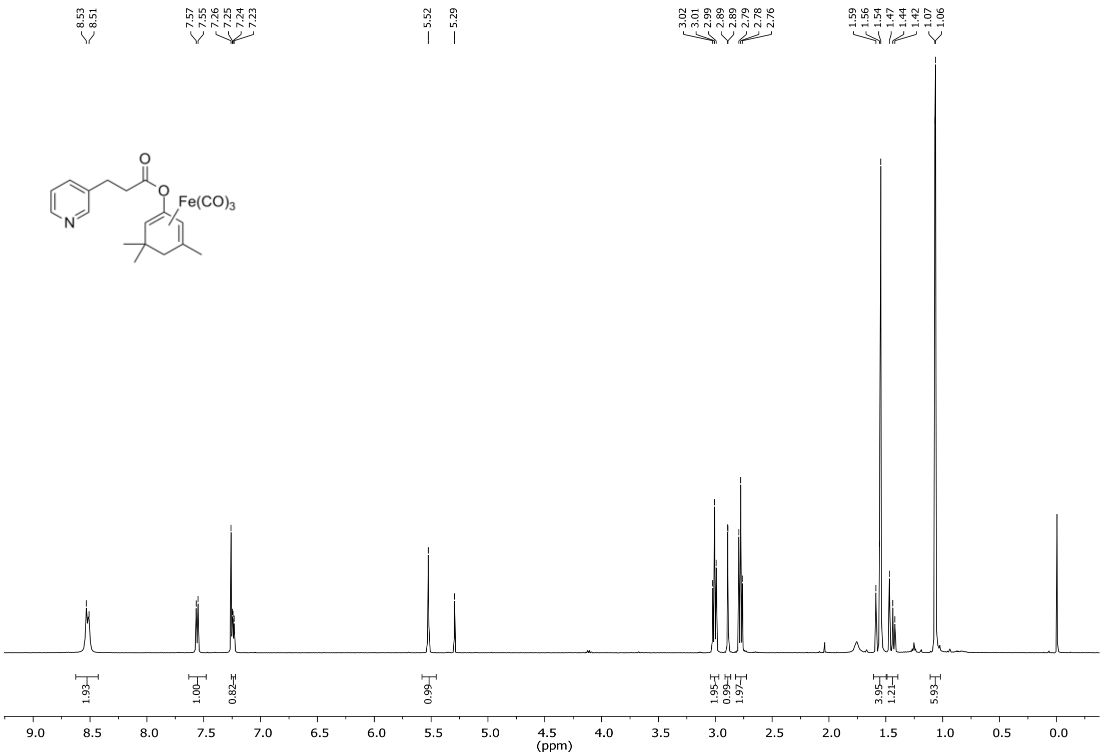

Figure S 39: ^{1}H NMR of (3,3,5-Trimethylcyclohexa-1,5-diene-$Fe(CO)_3$)-1-yl-3-pyridine propionate (rac-**13-A**) (500 MHz, $CDCl_3$)

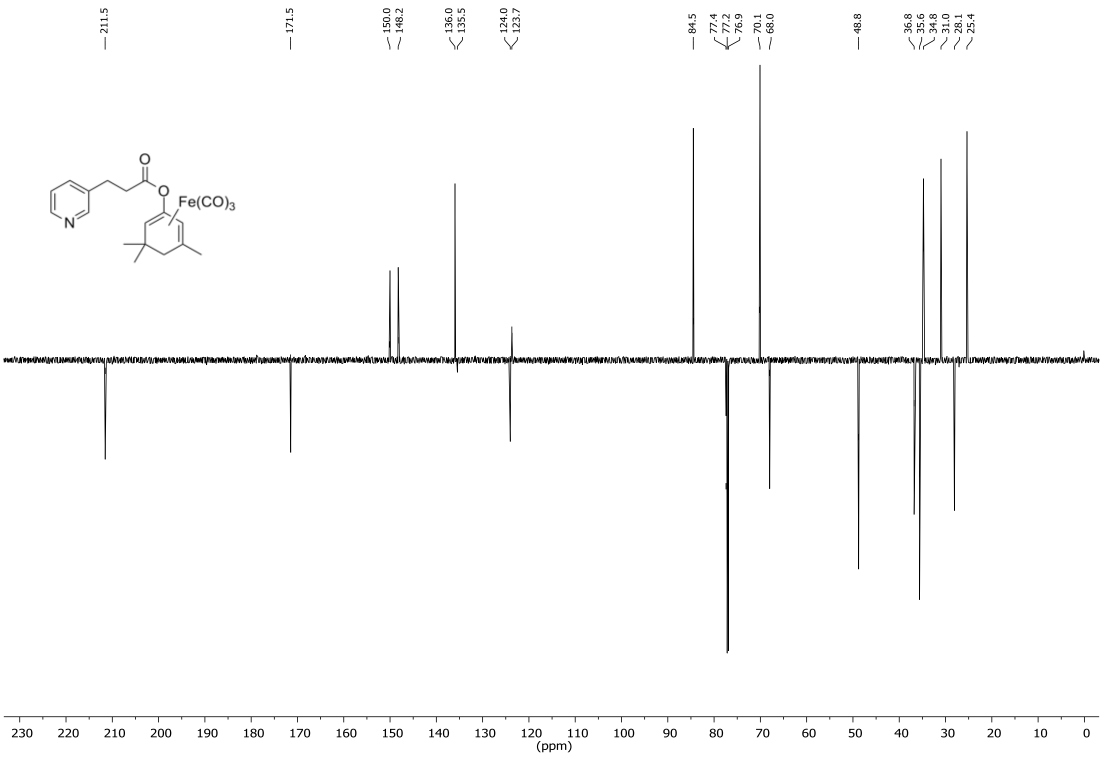

Figure S 40: ^{13}C NMR of (3,3,5-Trimethylcyclohexa-1,5-diene-$Fe(CO)_3$)-1-yl-3-pyridine propionate (rac-**13-A**) (125 MHz, $CDCl_3$).

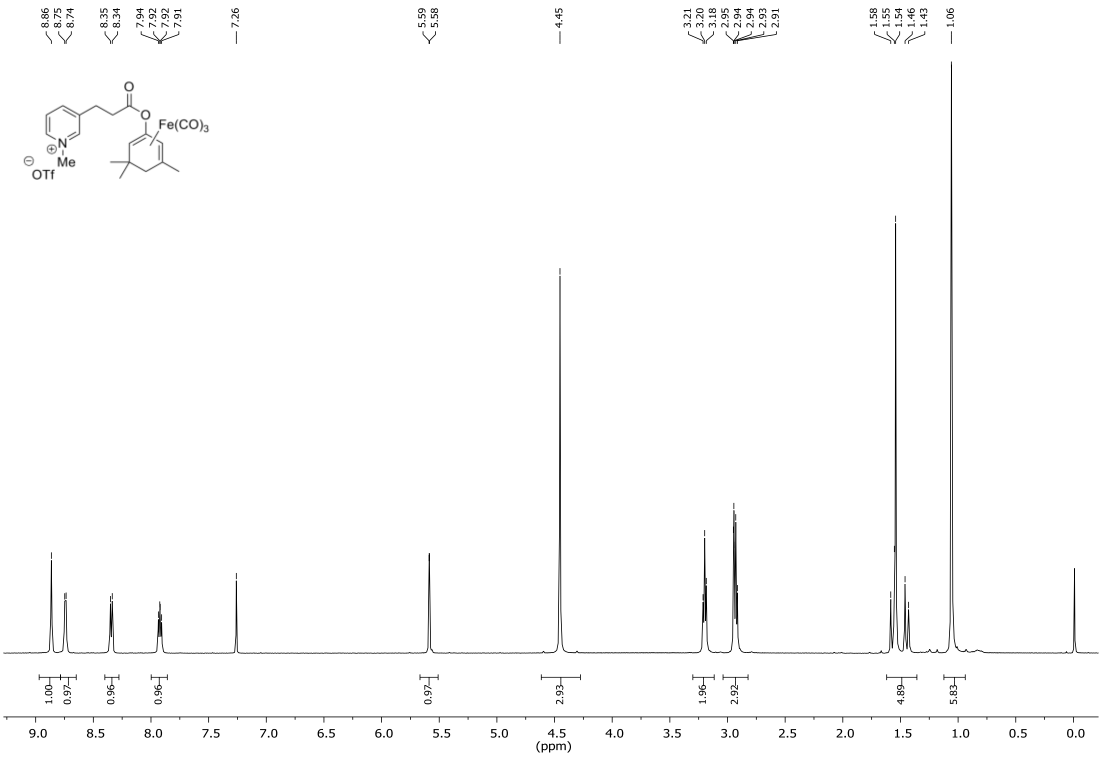

Figure S 41: ^{1}H NMR of **Mito-CORM 2-A'** (500 MHz, $CDCl_3$)

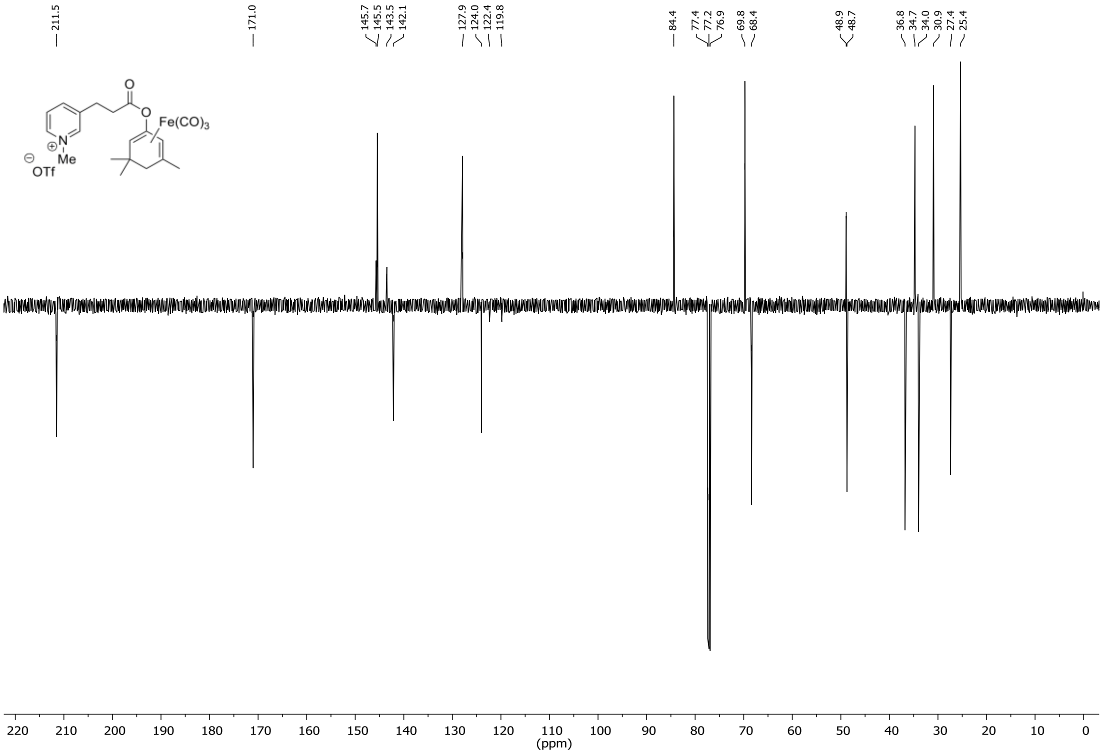

Figure S 42: ^{13}C NMR of **Mito-CORM 2-A'** (125 MHz, $CDCl_3$).

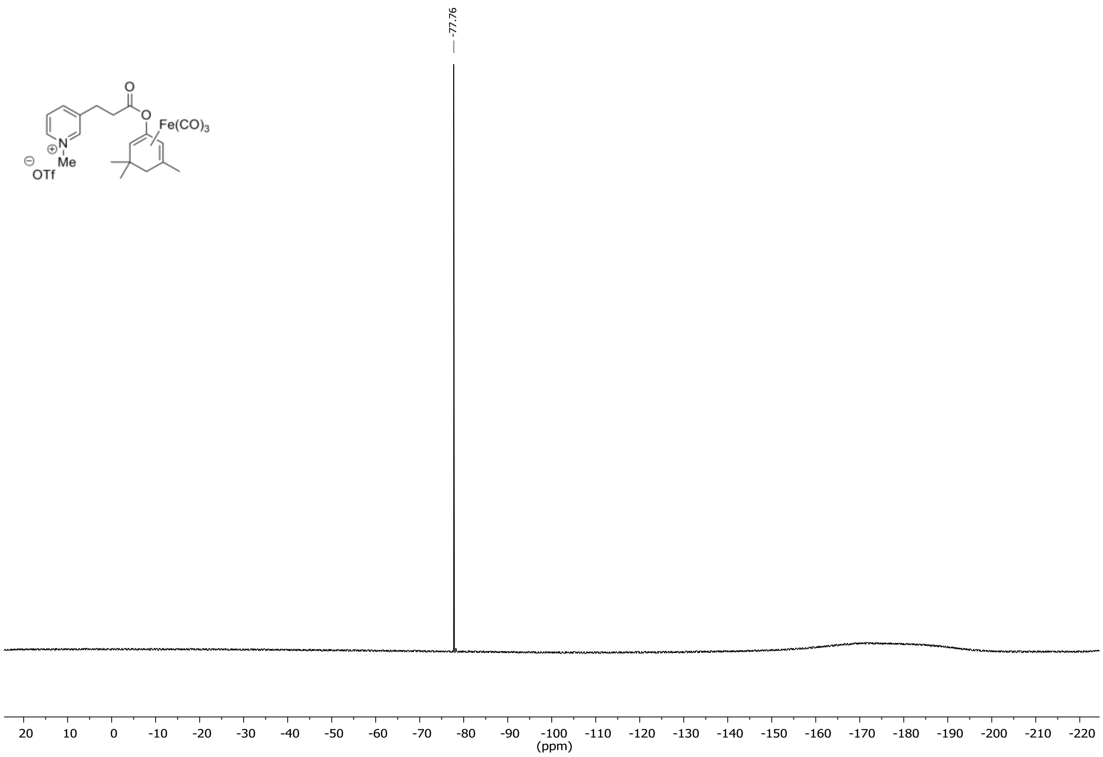

Figure S 43: ^{19}F NMR of **Mito-CORM 2-A’** (471 MHz, DMSO-d_6).

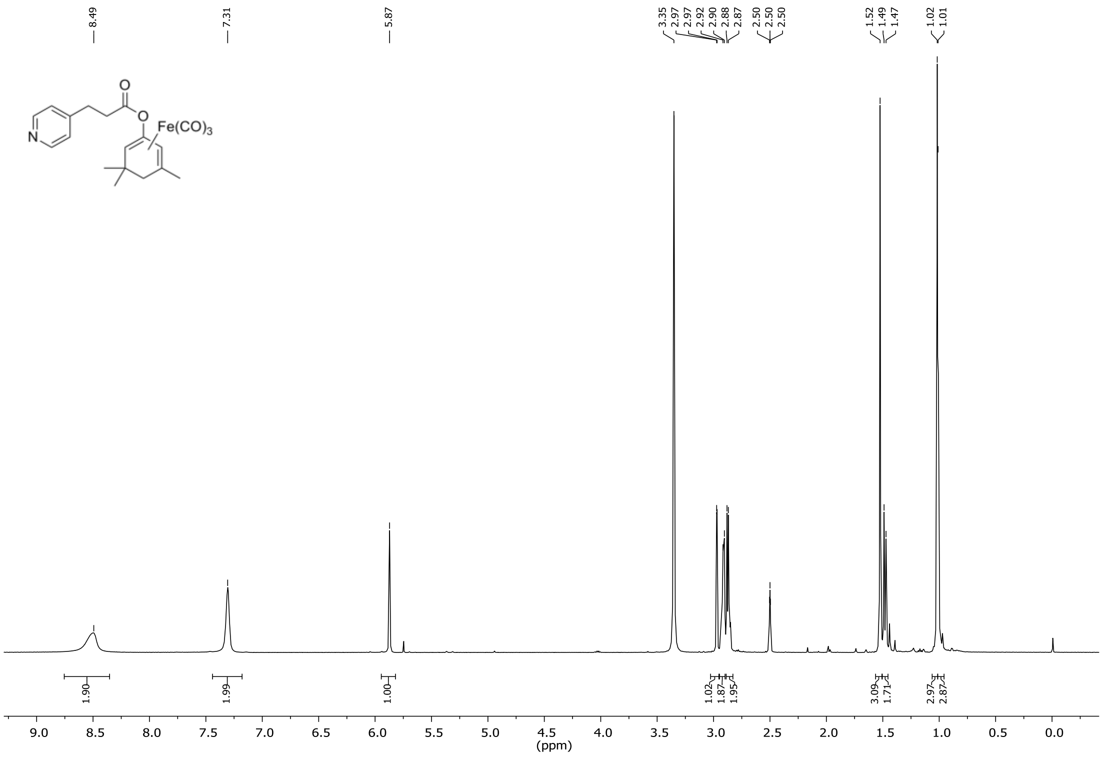

Figure S 44: ^{1}H NMR of (3,3,5-Trimethylcyclohexa-1,5-diene-Fe(CO)$_3$)-1-yl-4-pyridine propionate (rac-**14-A**) (500 MHz, DMSO-d_6).

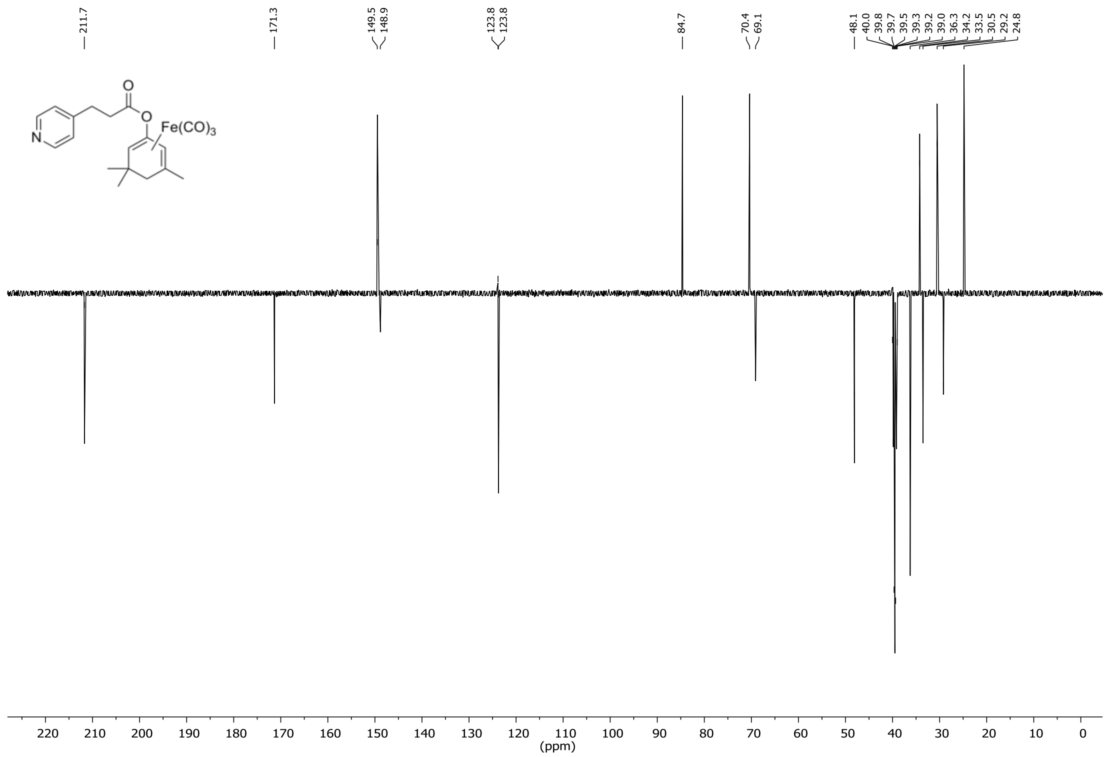

Figure S 45: ^{13}C NMR of (3,3,5-Trimethylcyclohexa-1,5-diene-$Fe(CO)_3$)-1-yl-4-pyridine propionate (rac-**14-A**) (125 MHz, DMSO-d_6).

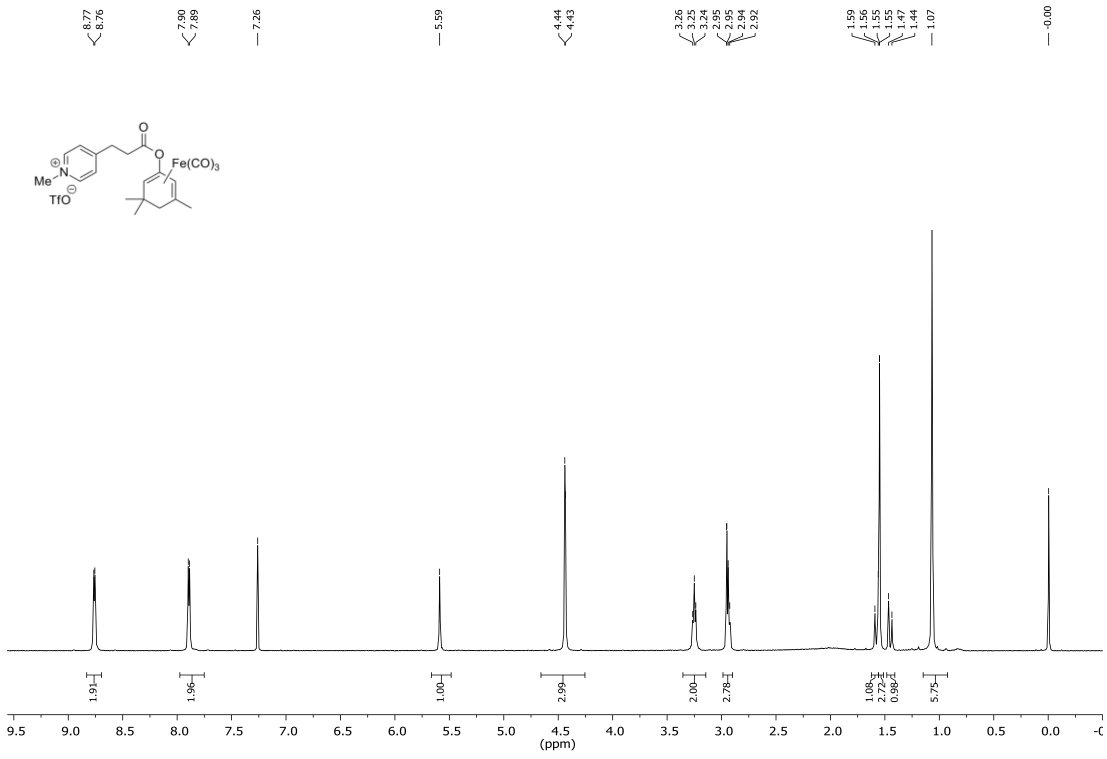

Figure S 46: ^{1}H NMR of **Mito-CORM 3-A’** (500 MHz, $CDCl_3$)

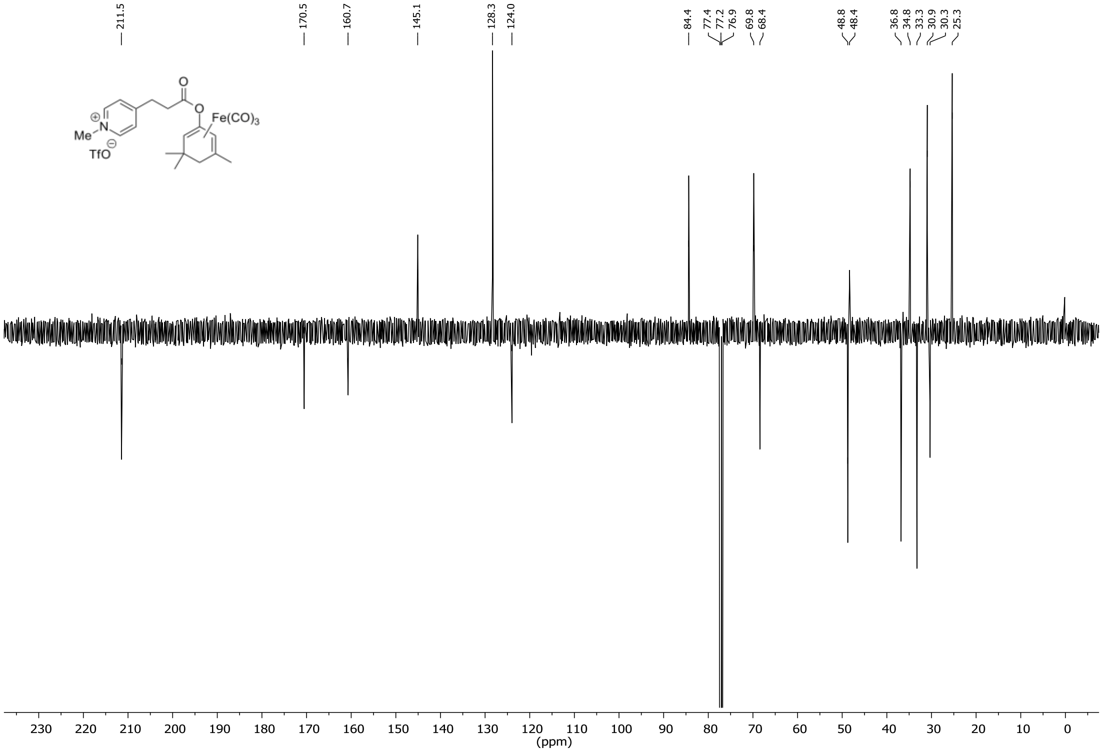

Figure S 47: ^{13}C NMR of **Mito-CORM 3-A’** (125 MHz, $CDCl_3$).

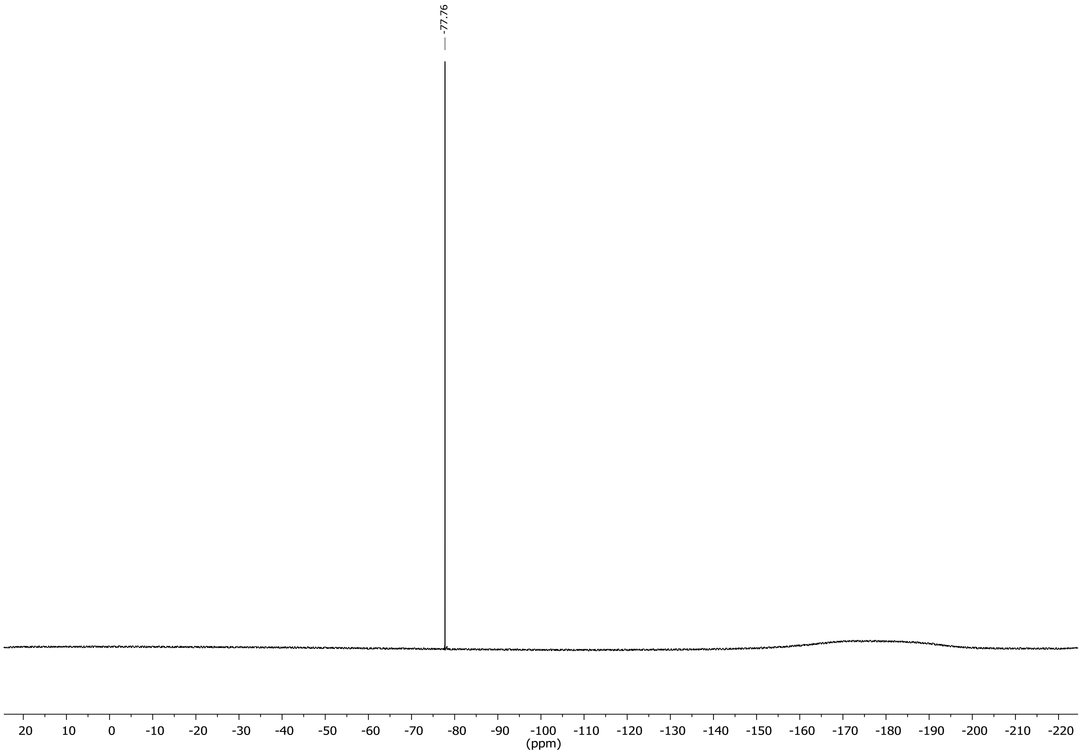

Figure S 48: ^{19}F NMR of **Mito-CORM 3-A’** (471 MHz, DMSO-d_6).

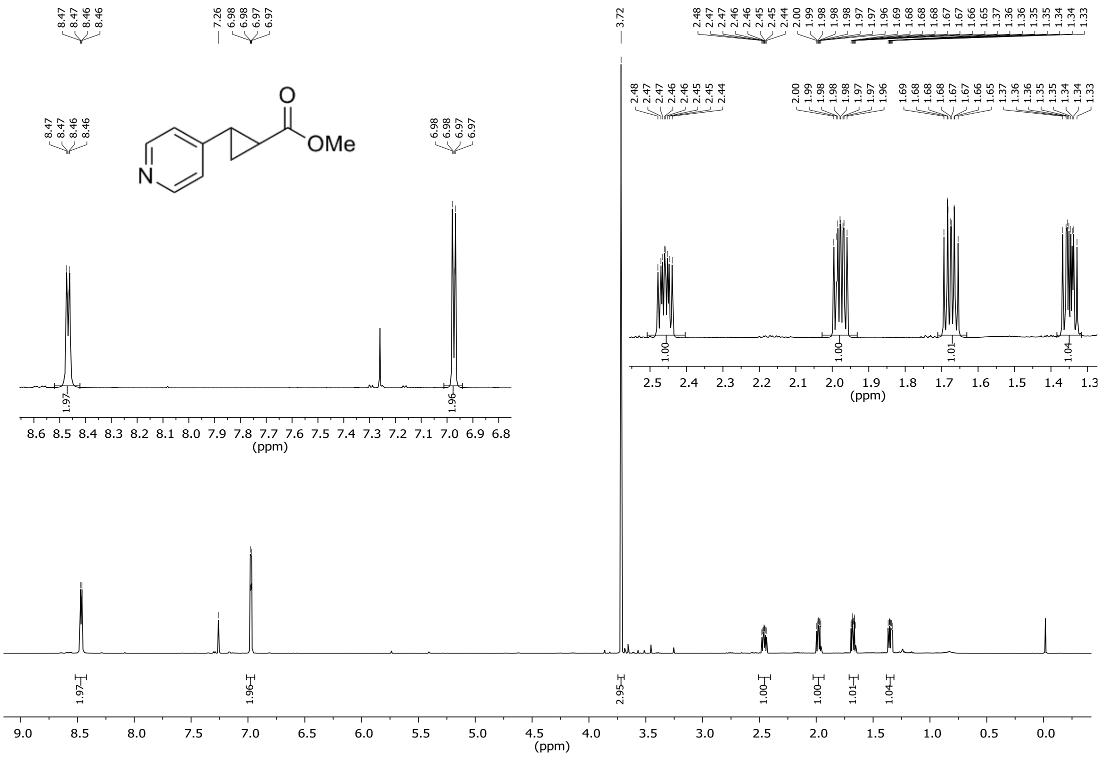

Figure S 49: ^{1}H NMR of (*rac*-**17**) (500 MHz, $CDCl_3$)

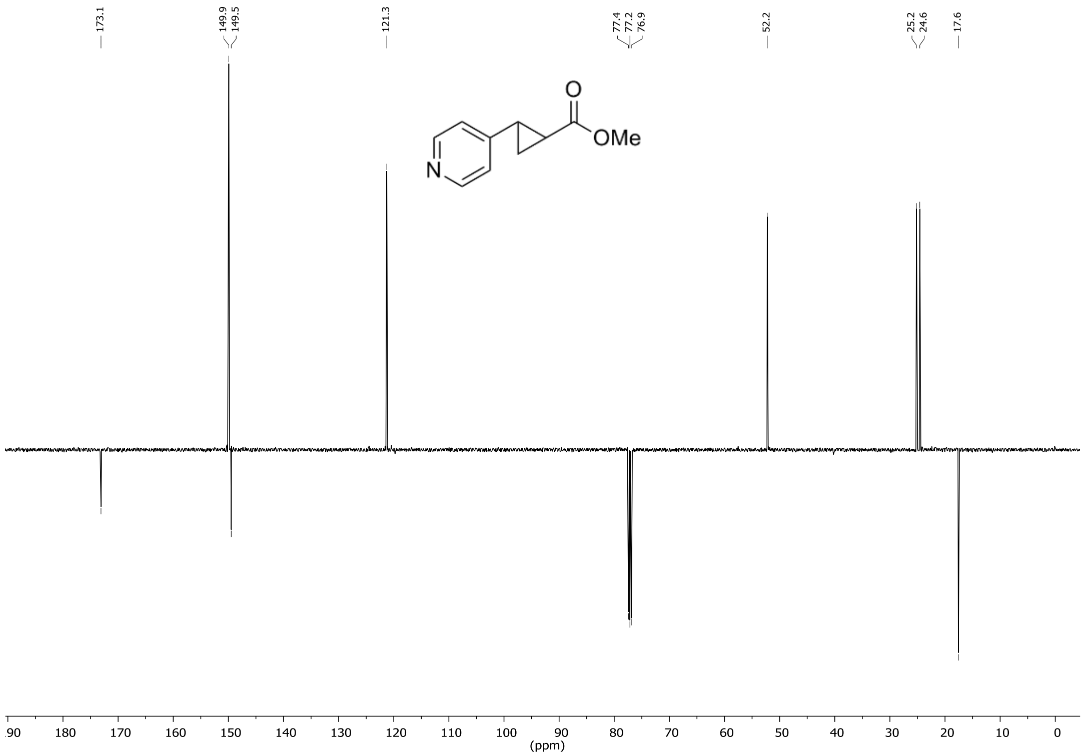

Figure S 50: ^{13}C NMR of (*rac*-**17**) (125 MHz, $CDCl_3$).

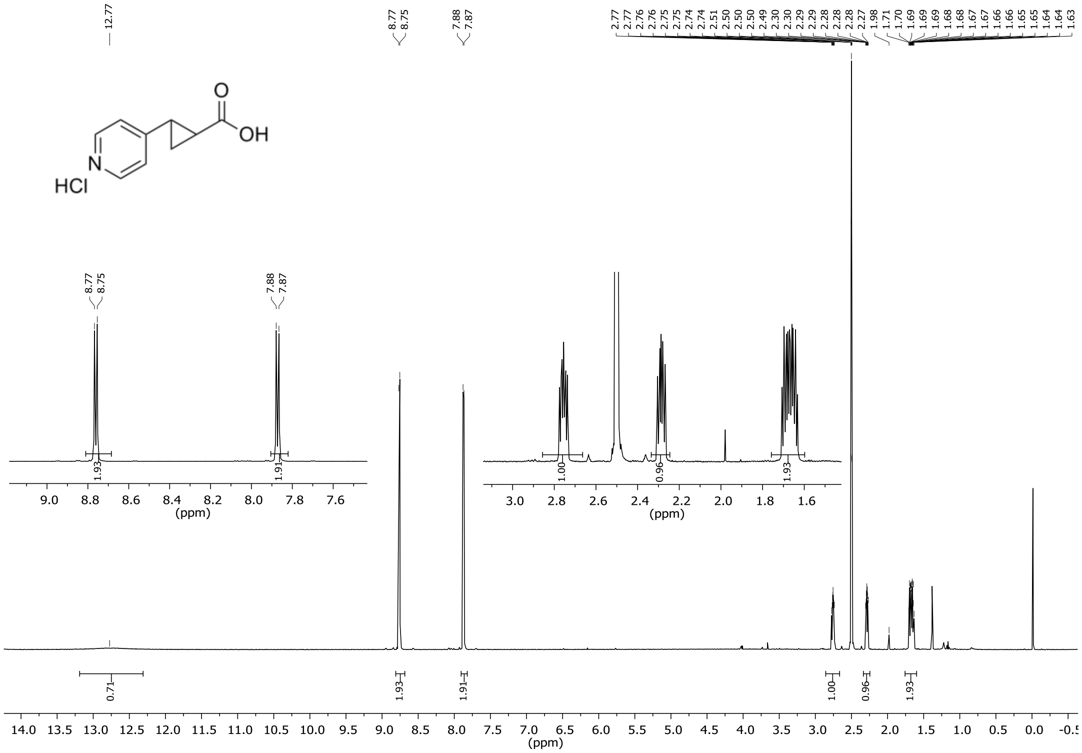

Figure S 51: ^{1}H NMR of (*rac*-**18**) (500 MHz, DMSO-d$_6$).

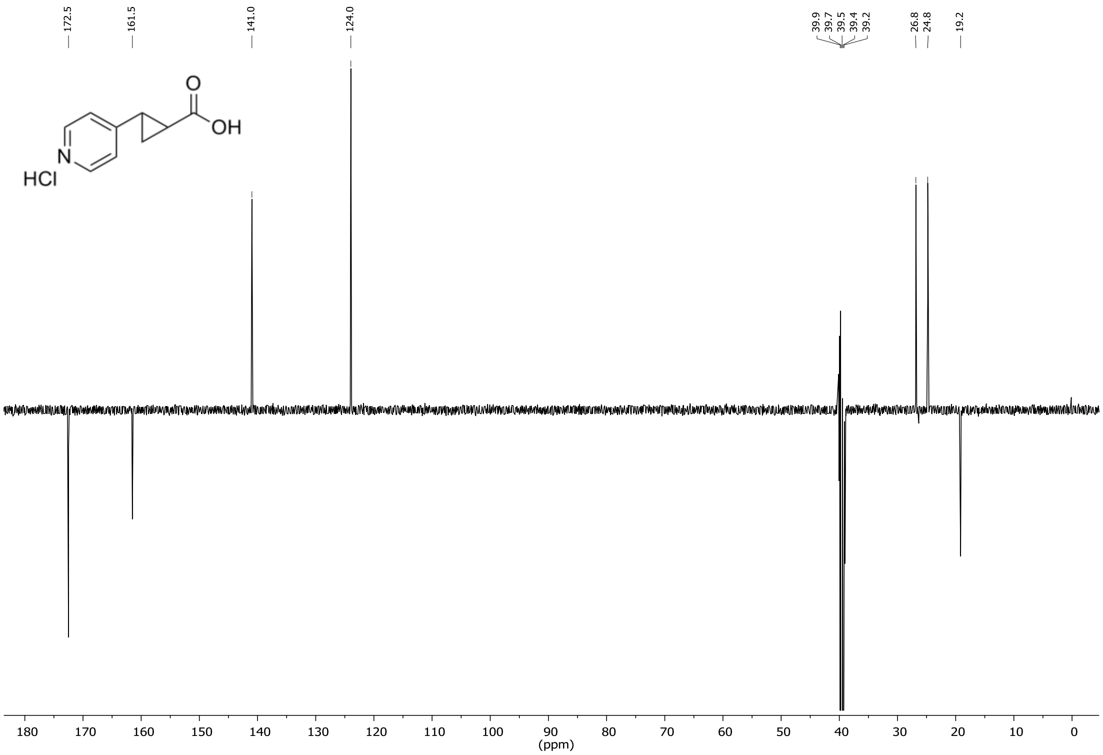

Figure S 52: ^{13}C NMR of (*rac*-**18**) (125 MHz, DMSO-d$_6$).

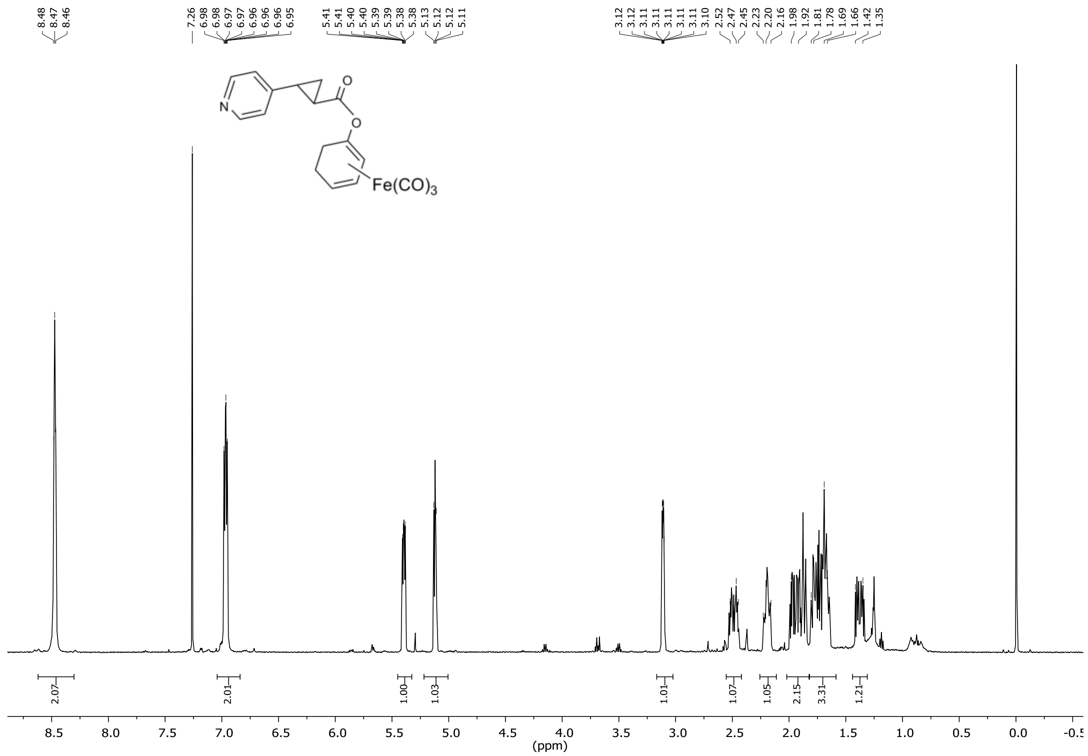

Figure S 53: ^{1}H NMR of (*ambo*-**19**) (500 MHz, $CDCl_3$)

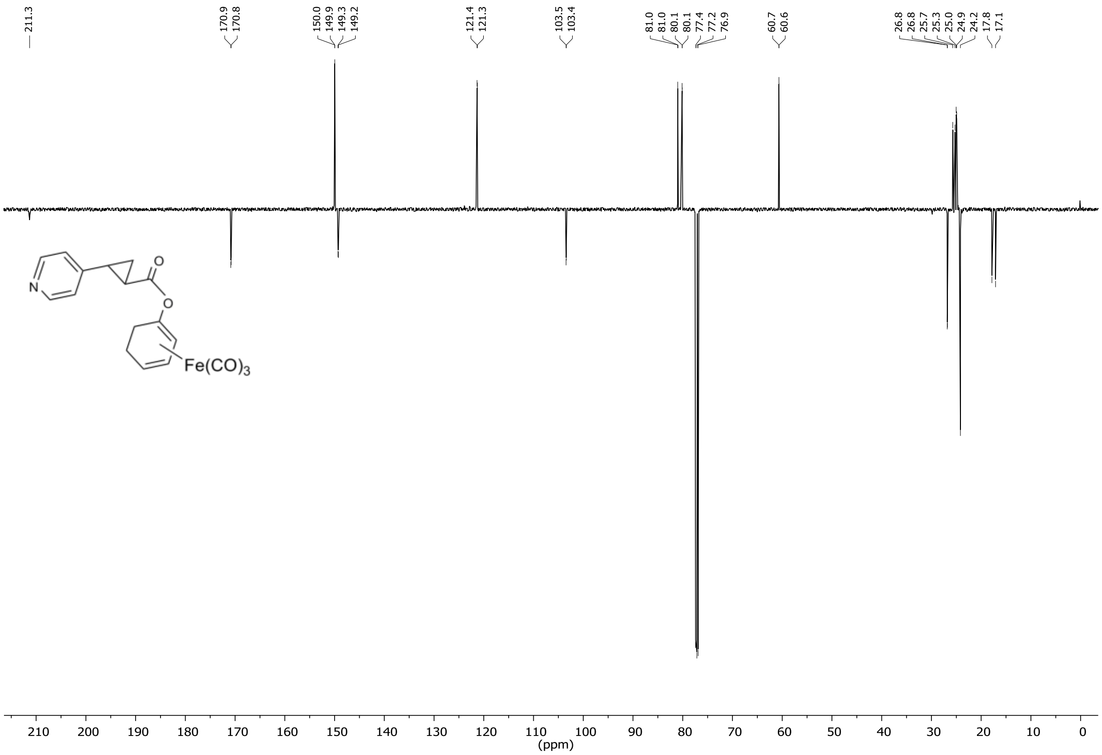

Figure S 54: ^{13}C NMR of (*ambo*-**19**) (125 MHz, $CDCl_3$).

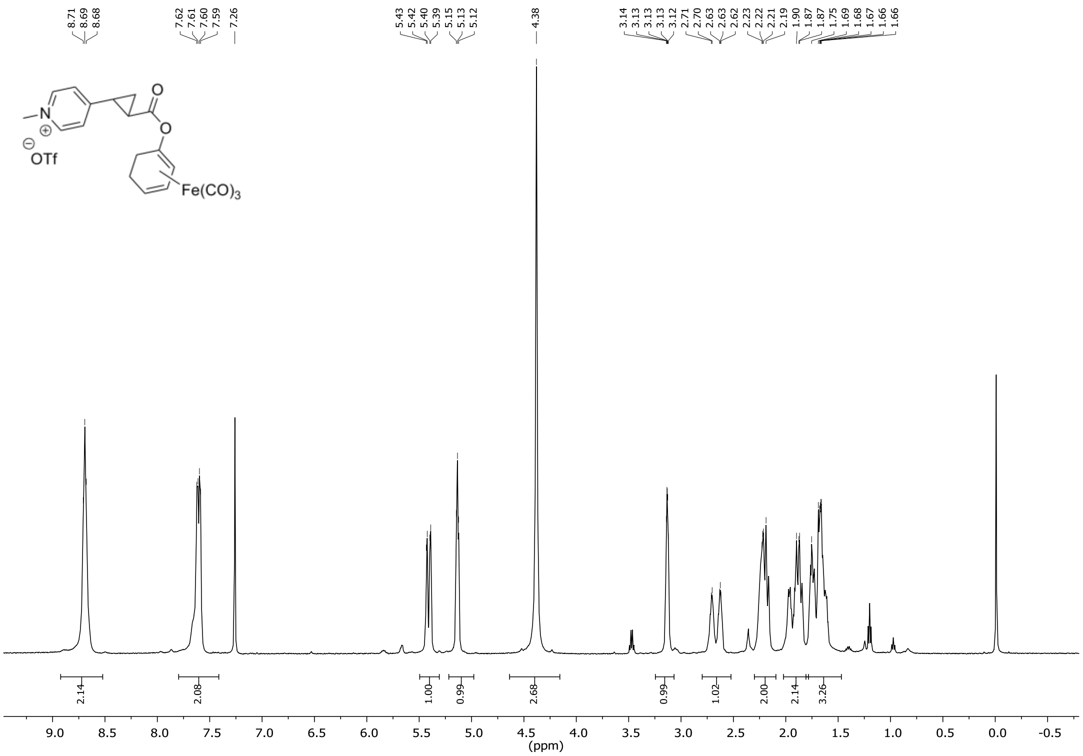

Figure S 55: ^{1}H NMR of **Mito-CORM 4-B** (500 MHz, $CDCl_3$)

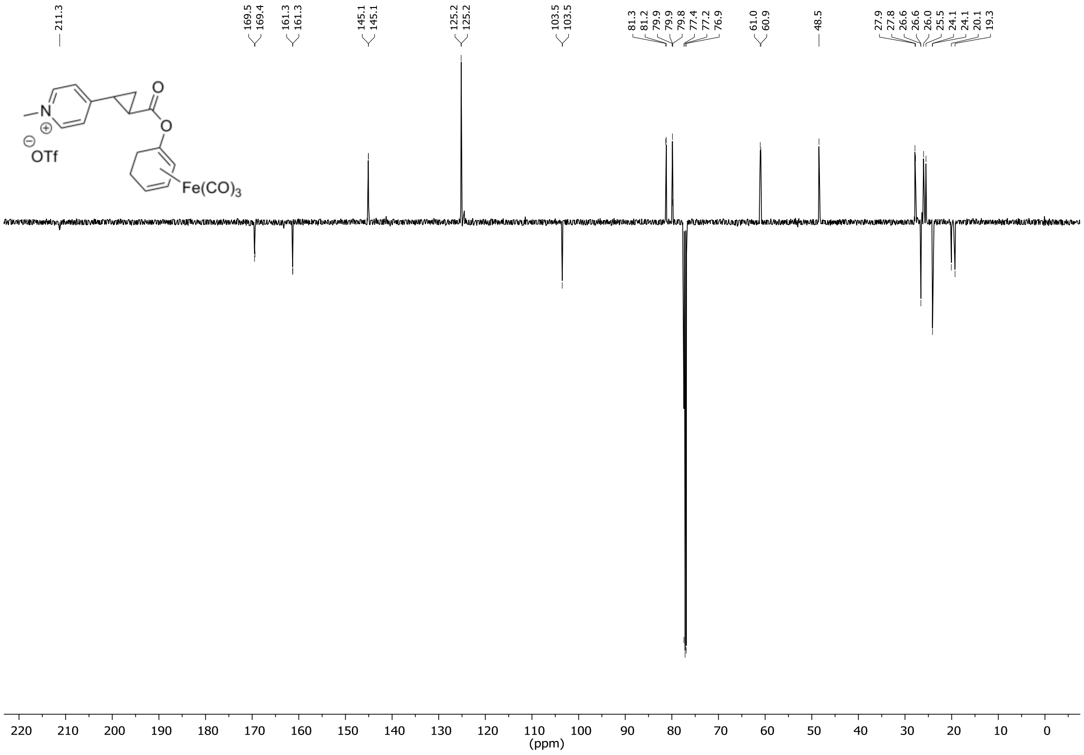

Figure S 56: ^{13}C NMR of **Mito-CORM 4-B** (125 MHz, $CDCl_3$).

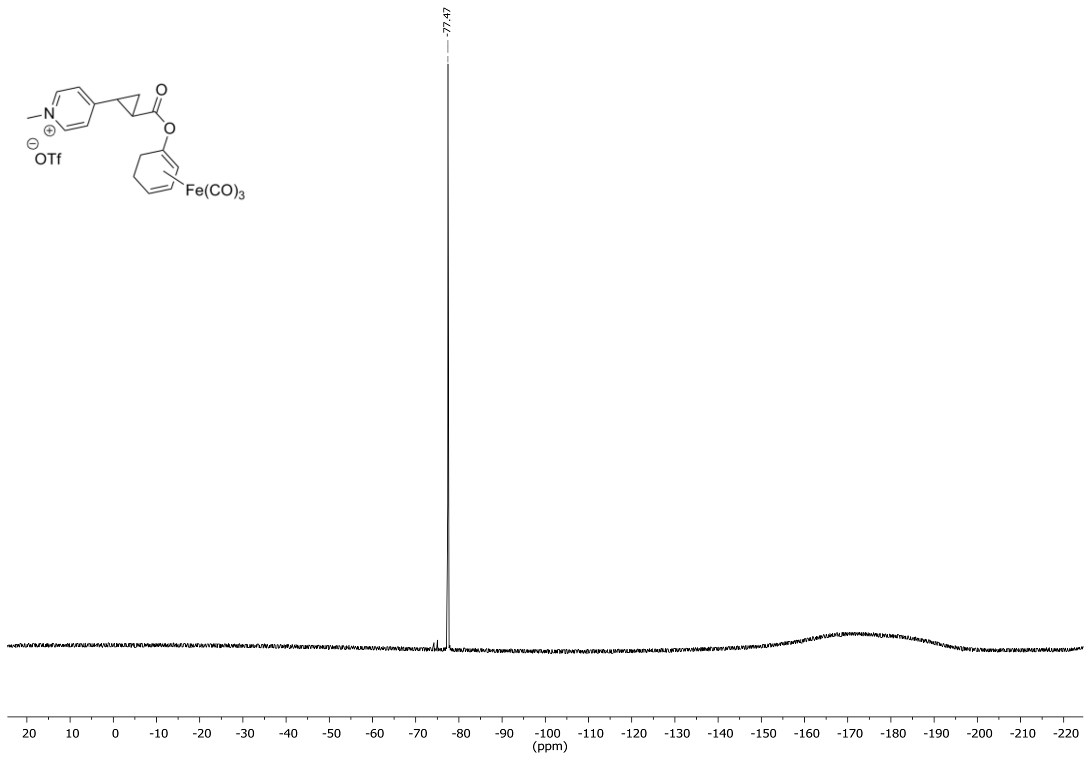

Figure S 57: ^{19}F NMR of **Mito-CORM 4-B** (471 MHz, $CDCl_3$).

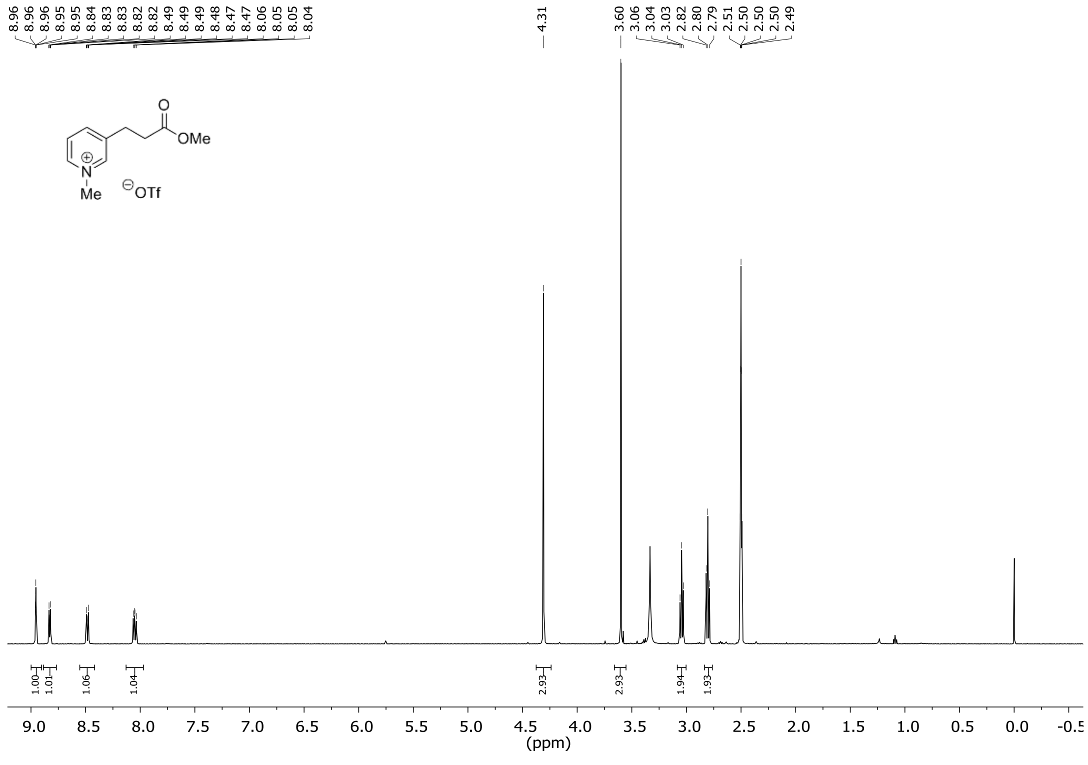

Figure S 58: ^{1}H NMR of **LHP551** (500 MHz, DMSO-d_6).

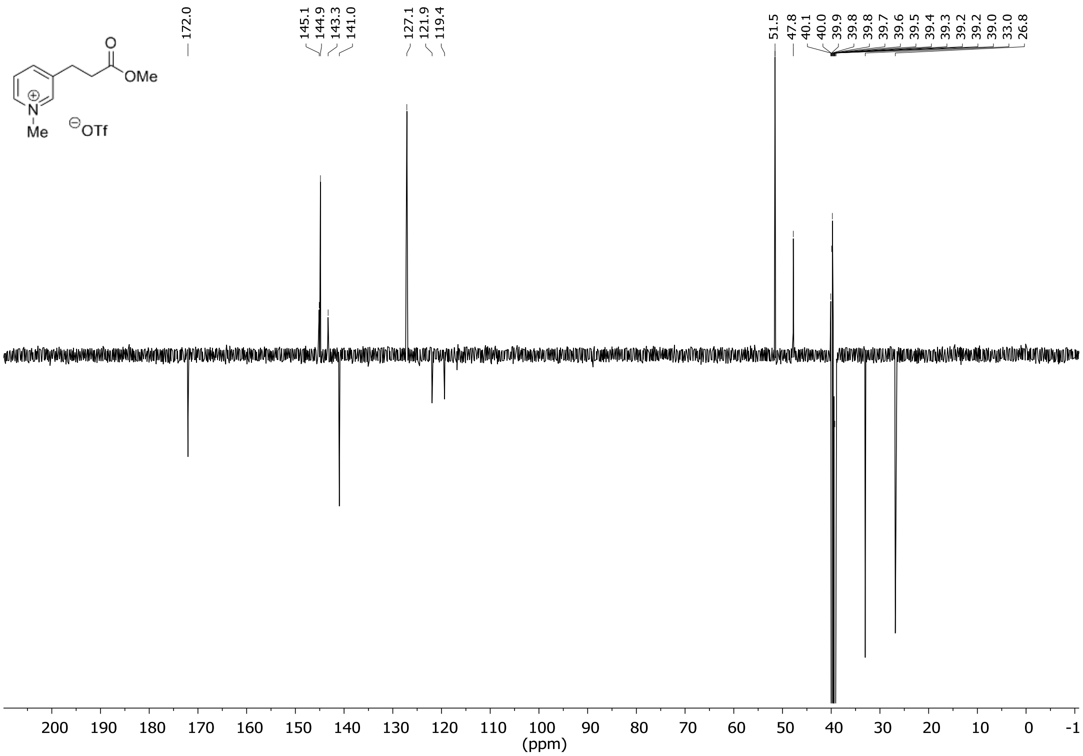

Figure S 59: ^{13}C NMR of **LHP551** (125 MHz, DMSO-d$_6$).

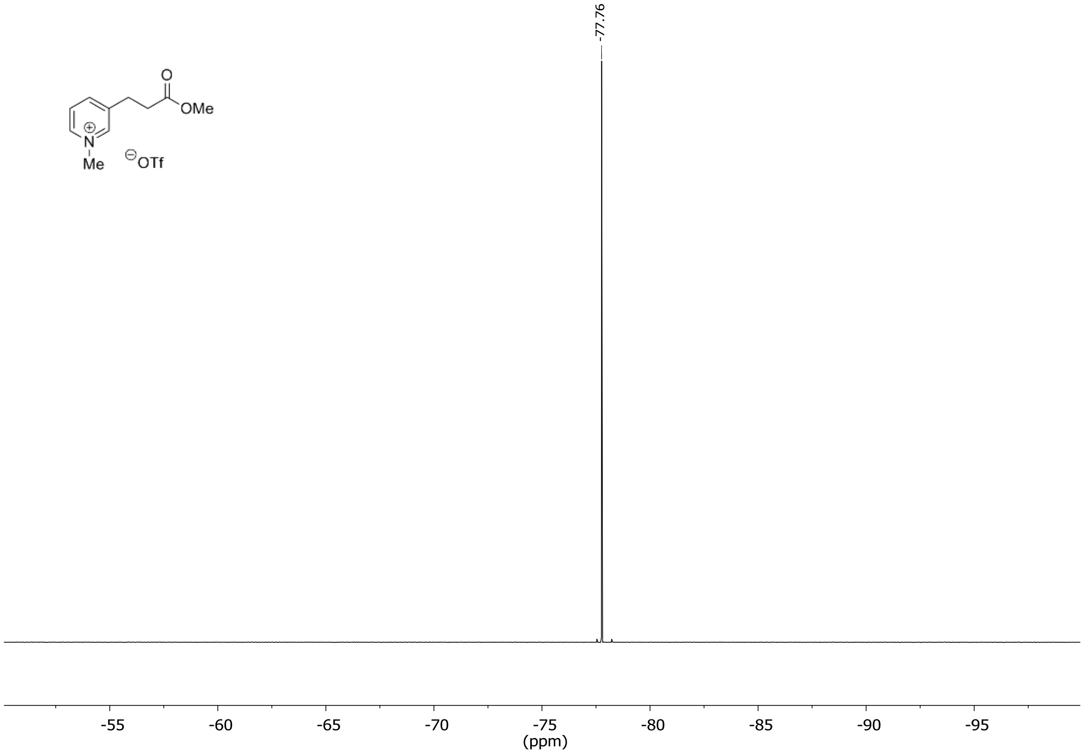

Figure S 60: ^{19}F NMR of **LHP551** (471 MHz, DMSO-d$_6$).

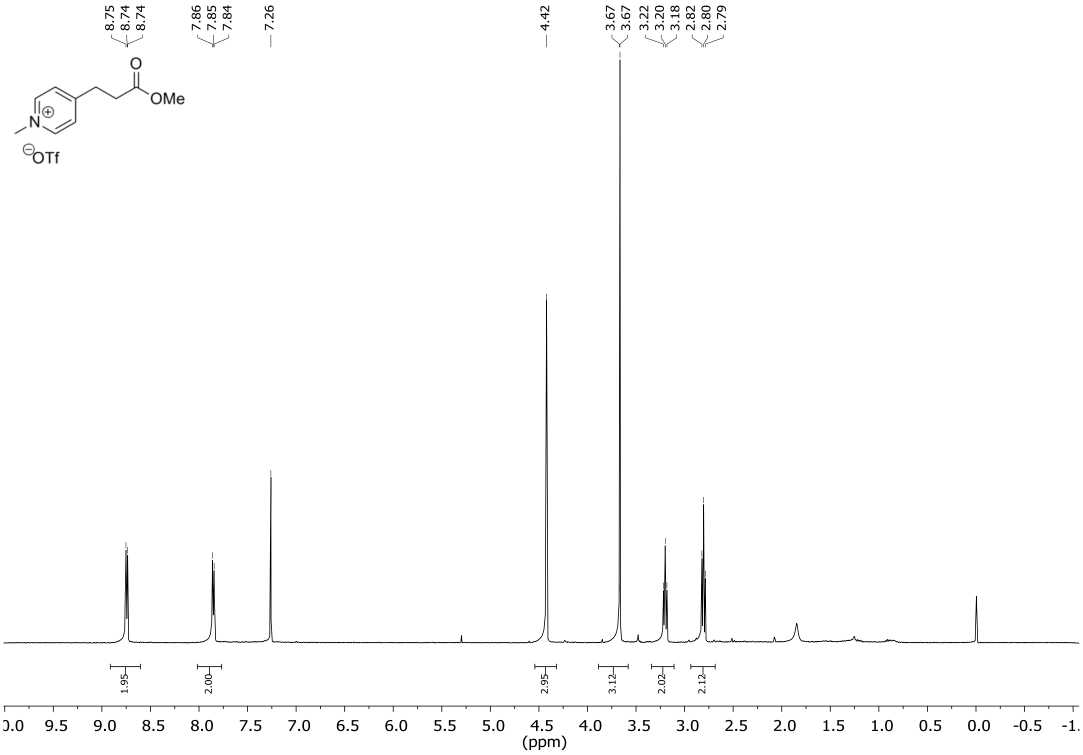

Figure S 61: ^{1}H NMR of **LHP551** (400 MHz, $CDCl_3$).

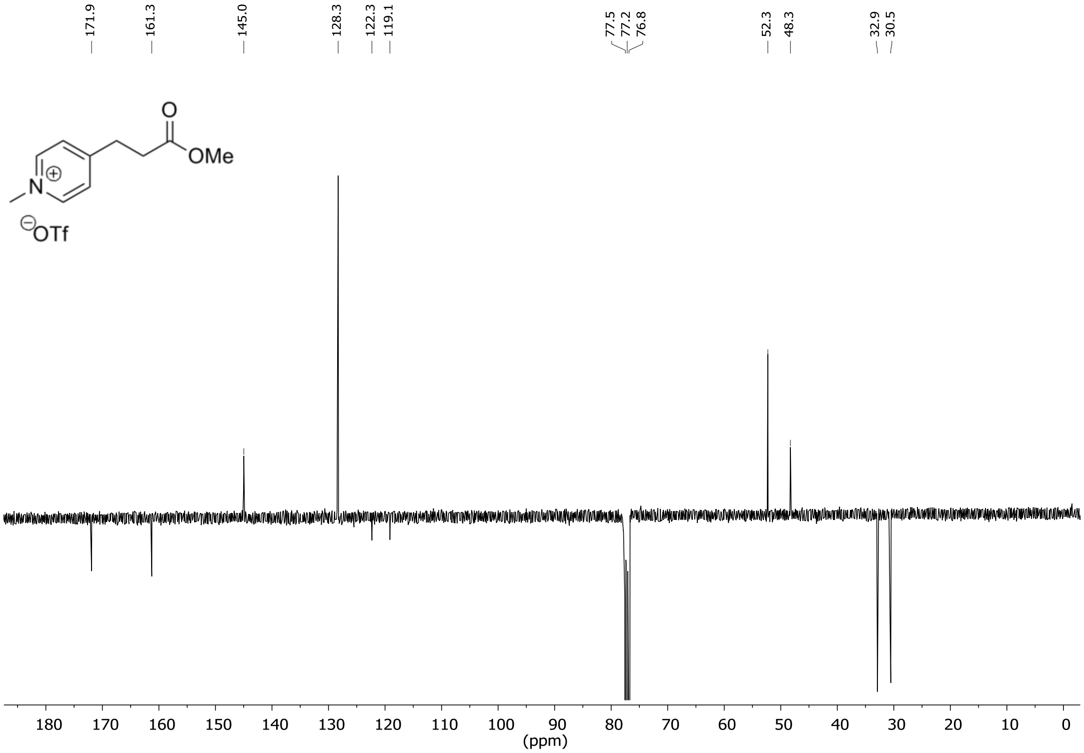

Figure S 62: ^{13}C NMR of **LHP551** (100 MHz, $CDCl_3$).

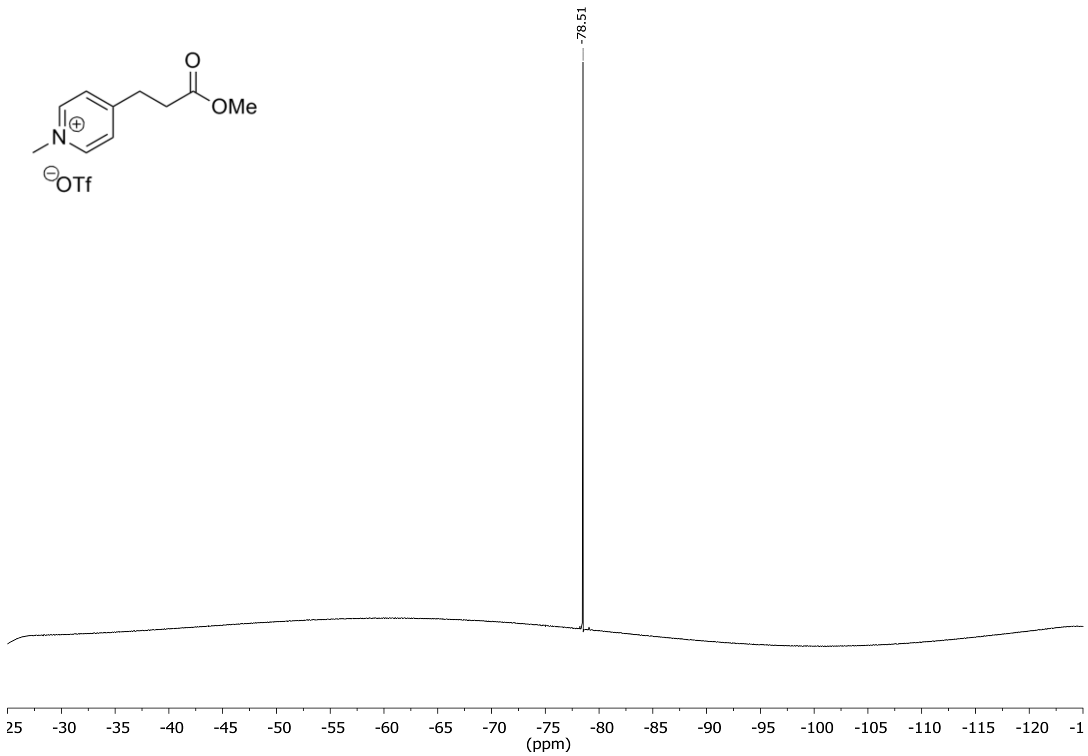

Figure S 63: ^{19}F NMR of **LHP551** (376 MHz, $CDCl_3$).

2. In situ quantification of CO release

Headspace GC system description

A *Thermo Scientific* Trace 1300 headspace gas chromatograph equipped with a *TriPlus* RSH autosampler was used. Detector type: thermal conductivity detector (TCD). Column: *Shin carbon* ST 100/120 1.0 mm × 2 m 1/16" OD silico. Software: Chromeleon® 7 Data System.

Headspace gas chromatography conditions

Gas-carrier: helium; flow: 15 mL/min; injector temperature: 200 °C; split flow: 150 mL/min; split rate: 10; detector temperature: 200 °C.

Method description: 0 – 2.5 min 35 °C, then to 70 °C with 20 °C/min rate, then 1 min at 70 °C, then to 35 °C with 20 °C/min rate, then 1 min at 35 °C. Injection volume: 50 µL.

Calibration

Eight headspace vials (*BGB Analytics*, cat. No. 200410-F, 10 mL) were filled with DMSO (0.2 mL) and phosphate buffer (0.1 M, pH = 7.4, 1.0 mL). Subsequently, the vials were closed with gas-tight silicon/PTFE septa crimp caps (*BGB Analytics*, cat. No. 20030500). Then, a defined gas volume was substituted by CO (0.00 mL, 0.05 mL, 0.10 mL, 0.25 mL, 0.50 mL, 1.00 mL, 1.50 mL, 2.00 mL). After equilibrating the vials for 10 min at 37 °C the composition of the gas phase was determined by headspace GC. These measurements were repeated three times and a calibration curve was generated.

In situ quantification of the CO release properties

The respective complex (36 µmol) was dissolved in DMSO (0.2 mL) and phosphate buffer (0.1 M, pH = 7.4, 1.0 mL) then PLE (15 mg) was added. The mixture was stirred at 37 °C and the amount of released CO assessed through headspace GC using the previously recorded calibration curve. As a control, samples without addition of esterase were analyzed in the same manner.

3. Crystallographic details

3.1. Crystal data and structure refinement for 1,5-Cyclohexadien-Fe(CO)$_3$)-1-yl isonicotinate (*rac*-2-A)

Measurements were carried out using a Bruker D8 Venture with κ geometry and a copper microfocus-source λ = 1.54178 Å). ORTEP drawings show thermal ellipsoids at the 50% probability level.

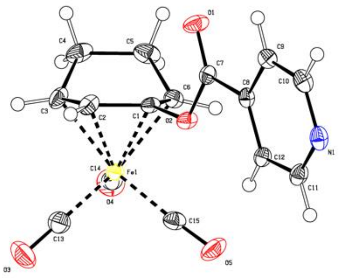

Empirical formula	$C_{15}H_{11}FeNO_5$	
Formula weight	341.10	
Temperature	100(2) K	
Wavelength	1.54178 Å	
Crystal system	Monoclinic	
Space group	P2$_1$/c	
Unit cell dimensions	a = 11.7916(3) Å	
	α= 90°.	
	b = 6.5305(2) Å	β= 106.4720(10)°.
	c = 19.2158(4) Å	γ = 90°.
Volume	1418.98(6) Å^3	
Z	4	

Density (calculated)	1.597 Mg/m^3
Absorption coefficient	8.752 mm^{-1}
F(000)	696
Crystal size	0.200 x 0.150 x 0.020 mm^3
Theta range for data collection	3.909 to 72.066°.
Index ranges	-14<=h<=14, -6<=k<=8, -23<=l<=23
Reflections collected	26229
Independent reflections	2774 [R(int) = 0.0614]
Completeness to theta = 67.679°	100.0 %
Absorption correction	Semi-empirical from equivalents
Max. and min. transmission	0.7536 and 0.3726
Refinement method	Full-matrix least-squares on F^2
Data / restraints / parameters	2774 / 0 / 211
Goodness-of-fit on F^2	1.058
Final R indices [I>2sigma(I)]	R1 = 0.0269, wR2 = 0.0653
R indices (all data)	R1 = 0.0297, wR2 = 0.0668
Largest diff. peak and hole	0.284 and -0.293 e.Å^{-3}

3.2. Crystal data and structure refinement for Mito-CORM 1-A

Measurements were carried out using a Bruker D8 Venture with κ geometry and a copper microfocus-source λ = 1.54178 Å). ORTEP drawings show thermal ellipsoids at the 50% probability level.

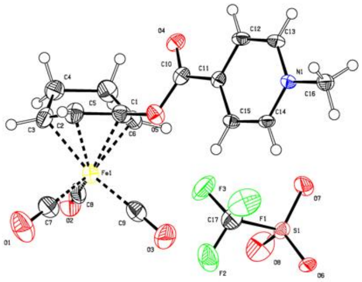

Empirical formula	$C_{17}H_{14}F_3FeNO_8S$	
Formula weight	505.20	
Temperature	100(2) K	
Wavelength	1.54178 Å	
Crystal system	Monoclinic	
Space group	$P2_1/c$	
Unit cell dimensions	a = 10.0557(2) Å	
	α= 90°.	
	b = 10.5115(2) Å	β= 90.208(2)°.
	c = 19.3613(4) Å	γ = 90°.
Volume	2046.49(7) Å^3	
Z	4	

Density (calculated)	1.640 Mg/m^3
Absorption coefficient	7.546 mm^{-1}
F(000)	1024
Crystal size	0.300 x 0.070 x 0.030 mm^3
Theta range for data collection	4.397 to 72.100°.
Index ranges	-10<=h<=12, -12<=k<=12, -23<=l<=23
Reflections collected	49890
Independent reflections	3983 [R(int) = 0.0878]
Completeness to theta = 67.679°	99.3 %
Absorption correction	Semi-empirical from equivalents
Max. and min. transmission	0.7536 and 0.3136
Refinement method	Full-matrix least-squares on F^2
Data / restraints / parameters	3983 / 0 / 281
Goodness-of-fit on F^2	1.065
Final R indices [I>2sigma(I)]	R1 = 0.0844, wR2 = 0.2166
R indices (all data)	R1 = 0.0925, wR2 = 0.2237
Largest diff. peak and hole	4.291 and -0.735 e.Å^{-3}

3.3. Crystal data and structure refinement for Mito-CORM 1-B

Measurements were carried out using a Bruker D8 Venture with κ geometry and a copper microfocus-source λ = 1.54178 Å). ORTEP drawings show thermal ellipsoids at the 50% probability level.

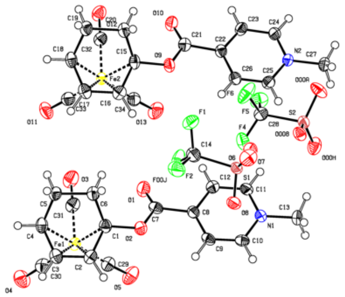

Empirical formula	$C_{17}H_{14}F_3FeNO_8S$	
Formula weight	505.20	
Temperature	100(2) K	
Wavelength	1.54178 Å	
Crystal system	Triclinic	
Space group	P-1	
Unit cell dimensions	a = 8.1924(7) Å	α= 81.760(5)°.
	b = 10.6179(9) Å	β= 86.690(5)°.
	c = 22.915(2) Å	γ = 84.839(5)°.
Volume	1962.7(3) Å^3	
Z	4	
Density (calculated)	1.710 Mg/m^3	

Absorption coefficient	7.868 mm^{-1}
F(000)	1024
Crystal size	0.150 x 0.040 x 0.008 mm^3
Theta range for data collection	1.950 to 72.631°.
Index ranges	-10<=h<=8, -13<=k<=13, -28<=l<=28
Reflections collected	79663
Independent reflections	7528 [R(int) = 0.1218]
Completeness to theta = 67.679°	97.3 %
Absorption correction	Multi-scan
Max. and min. transmission	0.7536 and 0.4534
Refinement method	Full-matrix least-squares on F^2
Data / restraints / parameters	7528 / 0 / 561
Goodness-of-fit on F^2	1.038
Final R indices [I>2sigma(I)]	R1 = 0.0577, wR2 = 0.1306
R indices (all data)	R1 = 0.0757, wR2 = 0.1416
Largest diff. peak and hole	0.785 and -0.433 e.Å^{-3}

3.4. Crystal data and structure refinement for *rac*-18

Measurements were carried out using a Bruker D8 Venture with κ geometry and a copper microfocus-source λ = 1.54178 Å). ORTEP drawings show thermal ellipsoids at the 50% probability level.

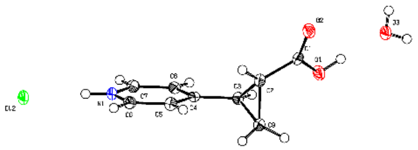

Empirical formula	$C_9H_{12}ClNO_3$	
Formula weight	217.65	
Temperature	100(2) K	
Wavelength	1.54178 Å	
Crystal system	Monoclinic	
Space group	$P2_1/c$	
Unit cell dimensions	a = 13.4602(4) Å	α= 90°.
	b = 8.8202(3) Å	β= 106.619(2)°.
	c = 8.9688(4) Å	γ = 90°.
Volume	1020.31(7) $Å^3$	
Z	4	
Density (calculated)	1.417 Mg/m^3	
Absorption coefficient	3.192 mm^{-1}	

F(000)	456
Crystal size	0.150 x 0.050 x 0.040 mm^3
Theta range for data collection	3.427 to 72.399°.
Index ranges	-16<=h<=16, -10<=k<=10, -11<=l<=10
Reflections collected	41124
Independent reflections	2018 [R(int) = 0.0427]
Completeness to theta = 67.679°	100.0 %
Absorption correction	Semi-empirical from equivalents
Max. and min. transmission	0.7536 and 0.5539
Refinement method	Full-matrix least-squares on F^2
Data / restraints / parameters	2018 / 0 / 143
Goodness-of-fit on F^2	1.074
Final R indices [I>2sigma(I)]	R1 = 0.0253, wR2 = 0.0694
R indices (all data)	R1 = 0.0257, wR2 = 0.0697
Largest diff. peak and hole	0.281 and -0.216 e.Å^{-3}

3.5. Crystal data and structure refinement for Mito-CORM 2-B

Measurements were carried out using a Bruker D8 Venture with κ geometry and a copper microfocus-source λ = 1.54178 Å). ORTEP drawings show thermal ellipsoids at the 50% probability level.

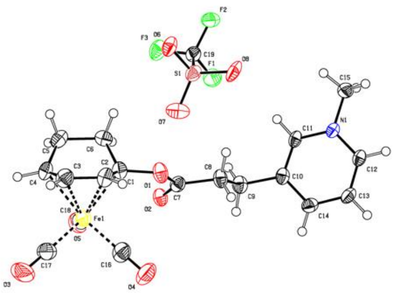

Empirical formula	$C_{19}H_{18}F_3FeNO_8S$	
Formula weight	533.25	
Temperature	100(2) K	
Wavelength	1.54178 Å	
Crystal system	Monoclinic	
Space group	P2₁/c	
Unit cell dimensions	a = 24.2935(13) Å	
	α= 90°.	
	b = 8.5652(4) Å	β= 98.251(3)°.
	c = 10.6980(5) Å	γ = 90°.
Volume	2202.98(19) Å^3	
Z	4	
Density (calculated)	1.608 Mg/m^3	
Absorption coefficient	7.043 mm^{-1}	
F(000)	1088	

Crystal size	0.100 x 0.050 x 0.020 mm^3
Theta range for data collection	1.837 to 72.239°.
Index ranges	-29<=h<=29, -10<=k<=10, -13<=l<=13
Reflections collected	52020
Independent reflections	4129 [R(int) = 0.1350]
Completeness to theta = 67.679°	95.2 %
Absorption correction	Semi-empirical from equivalents
Max. and min. transmission	0.7536 and 0.4834
Refinement method	Full-matrix least-squares on F^2
Data / restraints / parameters	4129 / 0 / 299
Goodness-of-fit on F^2	1.237
Final R indices [I>2sigma(I)]	R1 = 0.0956, wR2 = 0.2038
R indices (all data)	R1 = 0.1148, wR2 = 0.2098
Largest diff. peak and hole	0.713 and -0.511 e.$Å^{-3}$

4. Biological Procedures

4.1. Reagents

DPBS, Penicillin/Streptomycin, TrypLE Select 10X, Stripping Buffer (Thermofisher, MA, Waltham, USA), Endothelial cell growth media advanced, Endothelial cell growth supplement-mix (provitro AG, Berlin, Germany), FBS Gold (PAA laboratories GmbH, Pasching, Austria), Gelatine from porcine skin, Tween® 20, N,N'-Methylenebisacrylamide 29:1, N,N,N',N'-Tetramethylethylendiamine (TEMED), Collagenase CHH, 3-(4,5)-Dimethylthiazol-2-yl-2,5-diphenyltetrazolium bromide (MTT), Dimethyl sulfoxide (DMSO), Sodium pyruvate, L-glutamine, Glucose, 2-desoxyglucose (Sigma-Aldrich, MO, St. Louis, USA), Acetic acid (Honeywell, NJ, Morristown, USA), Coomassie Reagent (Pierce, IL, Rockford, USA), Laemmli Sample Buffer, 0.5 M Tris-HCl Buffer pH = 6.8, 1.5 M Tris-HCl Buffer pH = 8.8, 10X Tris/Glycine/SDS Buffer (Bio-Rad, CA, Hercules, USA), chemiluminescent HRP substrate (Western Lightning® Plus-ECL, PerkinElmer, MA, Waltham, USA), powdered milk (Carl Roth, Karlsruhe, Germany), Trizol (Invitrogen™, CA, Carlsbad, USA), Chloroform (Merck, Darmstadt, Germany), Nuclease-free water, SDS Roti® Stock 20%, Methanol, Ethanol ≥ 99.8% (Carl Roth, Karlsruhe, Germany), Seahorse XF Calibrant, Seahorse XF Base Media, Oligomycin, Carbonyl-cyanide-p-trifluoromethoxyphenyl-hydrazone (FCCP) (Agilent Technologies, Santa Clara, USA).

4.2. Cell culture and viability assays

Umbilical cords were obtained from healthy women (Department of Obstetrics, University Medical Center Mannheim) after written informed consent. Isolation was approved by the local ethics committee (Medizinische Ethikkommission II der Medizinischen Fakultät Mannheim Ruprechts-Karls-University Heidelberg (Approval number: 2015-518-MA)). All research was performed in accordance with the Declaration of Helsinki and in accordance with relevant guidelines/regulations. Cell isolation was performed according to the following protocol: First, residual blood was gently washed out of the umbilical cord using tap water. Then, both the proximal and distal ends of the vein were carefully distended and wrapped around a three-way valve each and tightened to them via zip ties. The vessel was flushed with DPBS once. Thereafter, sterile collagenase IV was filled in the vein, both three way valves were closed one after another to establish a mild intravenous pressure and the umbilical cord was put in a DPBS-filled petri dish and into the incubator (37 °C, 5 Vol.-% CO_2, 10 min.). After incubation, FBS Gold 10% was used to prepare the tube for collection of the cells and to wash out the collagenase IV of the vein to abolish collagenase activity. Collected cells were centrifuged at 1200 rpm for 5 minutes at room temperature. After centrifugation, the supernatant was discarded and the cell pellet was resuspended in 5 mL of completed endothelial cell growth media advanced (7.5% FBS Gold, 1% Penicillin/Streptomycin, endothelial cell supplement-Mix) and transferred to a gelatine-coated T25 cell culture flask (greiner bio-one, Frickenhausen, Germany). HUVECs were cultured at 37 °C and 5 Vol.-% CO_2. Cell media was renewed every four days. Cells were split when reaching confluency. To assess cytotoxicity of Mito-CORMs, cell viability was measured via MTT assays. Cells were seeded in gelatine-coated 96 well plates at a density of 10^5 cells/well and incubated for 24 h. Then, cells were treated with Mito-CORMs at different concentrations for another 24 h. Next, after the supernatant was discarded a 1:10 dilution of MTT (5 mg/ml) in completed cell media was added to the plate and incubated for 4 h. Thereafter, cells were incubated with MTT solvent (4 parts DMSO, 4 parts SDS 10%, 2 parts DBPS and 1.2% acetic acid) overnight. Optical density was determined in triplicate for each concentration at a wavelength for n=560 nm (background n=670 nm) using a TECAN M200 microplate reader. Survival rates were calculated according to the following formula: Survival [%] = (absorbance treated cells – absorbance background treated cells) / (absorbance control – absorbance background control) x 100.

4.3. Protein isolation and Western Blotting

Cells were lysed in 50 µL lysis buffer (20 mM Tris-HCl, 150 mM NaCl, 5 mM EDTA, 1% Triton X-100, 0.5% sodium deoxycholate, 1 mM dithiothreitol (DTT) containing proteinase and phosphatase inhibitors. Protein concentration measurements were performed in triplicate using the Coomassie Protein Assay Kit (Thermofisher, MA, Waltham, USA) according to standard microplate protocol. Samples consisted of 20 µg protein, 4 µL Laemmli Sample Buffer and the appropriate amount of distilled water to reach a final volume of 16 µL. Samples were denatured at 95 °C for 5 min before loading onto 10% SDS-polyacrylamide gels followed by semi-dry blotting on PVDF membranes (Roche Diagnostics, Mannheim, Germany). After blotting, membranes were blocked with milk buffer 5% (dry milk powder in TBS-Tween 0.1%) for 1 h to cover free binding sites followed by incubation with primary antibodies overnight at 4 °C thereafter. Next, membranes were washed three times for 10 minutes each in TBS-Tween 0.1% at room temperature prior to incubation with horseradish peroxidase HRP-conjugated secondary antibodies for 1 h at room temperature. Washing steps were repeated accordingly and the labelled secondary antibodies were detected by adding chemiluminescent HRP substrate based on luminol and detected by a Peqlab Fusion SL fluorescence- and chemiluminescence imaging system (Peqlab, Erlangen, Germany). Equal protein loading was confirmed by equal expression of GAPDH. The following primary and secondary antibodies were used and purchased from Santa Cruz, TX, Dallas, USA: Anti-VCAM (1:1,000), Anti-ICAM (1:1,000), Anti-HO1 (1:1,000), Anti-GAPDH (1:1,000), Anti-mouse (1:4,000) Anti-goat (1:4,000).

4.4. RNA isolation, cDNA synthesis and qPCR

RNA was isolated using Trizol reagent and subsequent DNase treatment and removal was performed using a DNA-free Kit (Invitrogen™, Carlsbad, CA, USA) according to manufacturers´ instructions. RNA purity and concentration were assessed using a TECAN infinite M200 microplate reader (TECAN Group AG, Maeddendorf, Switzerland). For cDNA synthesis, the purified RNA samples were diluted to 1 µg / 10 µL with RNAse free water. Next, the RT master mix was prepared using the High Capacity cDNA Reverse Transcription Kit (Applied Biosystems, MA, Waltham, USA) according to manufacturer´s instructions. cDNA synthesis was conducted using the Applied Biosystems 2720 Thermal Cycler adjusted to the following conditions: 10 min. at 25 °C, 120 min. at 37 °C, 5 min. at 85 °C. TaqMan™ Fast Advanced Master Mix (Applied

Biosystems, MA, Waltham, USA) was prepared according to manufacturer´s instructions and qPCR was performed using a StepOnePlus™ Real-Time PCR System (Thermofisher, MA, Waltham, USA). The following TaqMan assays were used and purchased from Thermofisher Scientific, MA, Waltham, USA: VCAM1 (No. Hs01003372_m1), ICAM1 (No. Hs00164932_m1), HMOX1 (No. Hs01110250_m1), and B2M (No. Hs00187842_m1) was used as a house-keeping gene. Gene expression was determined in triplicate.

Table S1: Influence of Mito-CORMs on ICAM-1 and CXCL1 mRNA expression

	**ICAM-1**		**CXCL1**	
	Fc[a]	P-value[b]	Fc	P-value
**Medium**	1	<0.0001	1	<0.0001
**TNFα**	445 ± 14	-	114 ± 0.6	-
**+ 2-A‘ [25 µM]**	352 ± 16	<0.0001	52 ± 0.2	<0.0001
**+ 3-A‘ [25 µM]**	614 ± 21	<0.0001	54 ± 0.7	<0.0001
**+ 2-B [50 µM]**	474 ± 19	ns	67 ± 0.8	<0.0001
**+ 3-B [50 µM]**	280 ± 4	<0.0001	34 ± 1.9	<0.0001

a: Fold change compared to medium, b: P-value compared to TNFα

4.5. Seahorse metabolic analysis

Energy phenotyping assays were performed using a Seahorse XFe96 Analyzer (Agilent Technologies, Santa Clara, USA). 2.5x104 cells were seeded out in 100 µl of completed HUVEC media per gelatine-coated well and incubated overnight with different concentrations of Mito-CORMs. Prior to measuring, cells were washed with seahorse XF DMEM assay media (Agilent Technology, Santa Clara, USA), supplemented with 2 mM L-glutamine, 1 mM sodium pyruvate and 10 mM glucose and then incubated therein for 1 h (CO_2-free). Energy phenotyping assays were performed upon simultaneous stimulation with oligomycin (1 µM) and FCCP (1 µM). Results were normalized by protein concentration measurements of each wells´ content via Bradford assays once experiments were finished.

5. References

[1] S. Romanski, B. Kraus, M. Guttentag, W. Schlundt, H. Rücker, A. Adler, J.-M. Neudörfl, R. Alberto, S. Amslinger, H.-G. Schmalz, *Dalton Trans*. **2012**, *41*, 13862-13875

[2] S. Romanski, B. Kraus, U. Schatzschneider, J.-M. Neudörfl, S. Amslinger, H.-G. Schmalz, *Angew. Chem. Int. Ed*. **2011**, *50*, 2392-2396.

[3] L. Hemmersbach, S. Romanski, S. Botov, A. Adler, J.-M. Neudörfl, H.-G. Schmalz, *Organometallics* **2021**, *40*, 2909-2914.

[4] L. Li, J. C. Stimac, L. M. Geary, *Tetrahedron Lett*. **2017**, *58*, 1379-1381.

[5] G. A. POE; K. HAROLD (MALLINCKRODT CHEMICAL WORKS), US3478038A, **1969**. [*Chem. Abstr*., *72*, 31630]

[6] G. Sirasani, L. Tong, E. P. Balskus, *Angew. Chem. Int. Ed.* **2014**, *53*, 7785-7788.

[7] D. Leow, Y.-H. Chen, T.-H. Hung, Y. Su, Y.-Z. Lin, *Eur. J. Org. Chem.* **2014**, *2014*, 7347-7352.
